# Supplementary material for: Frustrated flexibility in metal-organic frameworks
Source: Nat Commun. 2021 Jul 2;12:4097. doi: 10.1038/s41467-021-24188-4 (PMC8253802; doi:10.1038/s41467-021-24188-4)
Supplement: Supplementary file 1 — Supplementary Information [file 41467_2021_24188_MOESM1_ESM.pdf]

Supplementary Information

Frustrated flexibility in metal-organic frameworks

Roman Pallach[1], Julian Keupp[2], Kai Terlinden[1], Louis Frentzel-Beyme[1], Marvin Kloß[1], Andrea Machalica[1], Julia Kotschy[3], Suresh K. Vasa[3], Philip A. Chater[4], Christian Sternemann[5], Michael T. Wharmby[6], Rasmus Linser[3], Rochus Schmid[2], Sebastian Henke[1]*

[1]Anorganische Chemie, Fakultät für Chemie und Chemische Biologie, Technische Universität Dortmund, Otto-Hahn-Straße 6, 44227 Dortmund, Germany.

[2]Computational Materials Chemistry Group, Fakultät für Chemie und Biochemie, Ruhr-Universität Bochum, 44801 Bochum, Germany.

[3]Physikalische Chemie, Fakultät für Chemie und Chemische Biologie, Technische Universität Dortmund, Otto-Hahn-Straße 6, 44227 Dortmund, Germany.

[4]Diamond Light Source, Harwell Campus, Didcot, Oxfordshire, OX11 0DE, UK.

[5]Fakultät Physik/DELTA, Technische Universität Dortmund, Maria-Goeppert-Mayer Str. 2, 44221 Dortmund, Germany.

[6]Deutsches Elektronen-Synchrotron (DESY), Notkestraße 85, 22607 Hamburg, Germany.

Email: sebastian.henke@tu-dortmund.de

Contents

Supplementary Methods 1 - Syntheses 2

Supplementary Methods 2 - Powder X-ray diffraction (PXRD) 12

Supplementary Methods 3 - Single crystal X-ray diffraction (SCXRD) 27

Supplementary Methods 4 - Solution ^{1}H NMR spectroscopy of digested MOF samples 35

Supplementary Methods 5 - X-ray pair distribution function analysis 39

Supplementary Methods 6 - IR spectroscopy 45

Supplementary Methods 7 - Thermal analysis 50

Supplementary Methods 8 - Variable temperature PXRD 54

Supplementary Methods 9 - Isothermal gas sorption 58

Supplementary Methods 10 - In situ gas sorption PXRD 64

Supplementary Methods 11 - Molecular dynamics simulations 69

Supplementary Methods 12 - Experimental data on MOF-5-CX with X = 9, 10 84

Supplementary References 90

Supplementary Methods 1 - Syntheses

Supplementary Methods 1.1 – Synthesis of H_2(CX-bdc)

All *alkoxy*-functionalized 2,5-diOC_xH_{x+1}-bdcH_2 linkers were prepared by use of Williamson ether synthesis starting from 2,5-dihydroxy-terephthalic acid dimethylester and the corresponding *n*-bromoalkane according to the literature.[1] A typical synthesis procedure is described below.

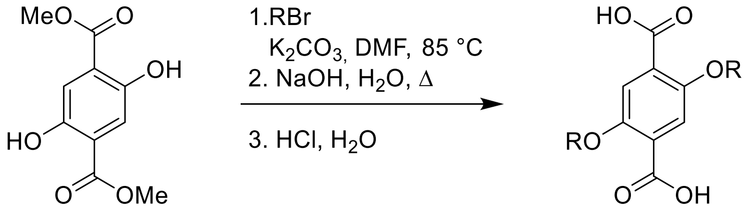

To a suspension of 2,5-dihydroxy-terephthalic acid dimethylester (1.00 g, 4.42 mmol) and K_2CO_3 (3.06 g, 22.11 mmol) in 30 ml DMF, RBr (13.26 mmol) was added dropwise and the mixture was heated at 85 °C for 4 h. The solvent was removed *in vacuo*, 50 mL of aqueous NaOH (50.0 mmol) were added and the reaction mixture was refluxed for 14 h. At room temperature, the solution was acidified by dropwise addition of aqueous HCl (15%). The precipitate was filtered off and thoroughly washed with water. Afterwards, the solid was dried under dynamic vacuum at 80 °C for 16 h. The product was obtained as an off-white powder in yields of ~90%. In rare cases, when the product contained unreacted educts, recrystallisation from EtOAc or DMF afforded pure products.

2,5-Diethoxy-1,4-benzenedicarboxylic acid, H_2(C2-bdc), ^{1}H NMR (500 MHz, DMSO-d_6): δ 12.93 (s, 2 H, COO–H), 7.26 (s, 2 H, Ar–H), 4.04 (q, J = 7.0 Hz, 4 H, OCH_2), 1.29 (t, J = 6.87 Hz, 6 H, CH_3) ppm; ^{13}C NMR (125.75 MHz, DMSO-d_6): δ 166.86, 150.30, 125.56, 115.64, 64.92, 14.70 ppm.

2,5-Dipropoxy-1,4-benzenedicarboxylic acid (H_2(C3-bdc), ^{1}H NMR (500 MHz, DMSO-d_6): δ 12.89 (s, 2 H, COO–H), 7.26 (s, 2 H, Ar–H), 3.94 (t, J = 6.4 Hz, 4 H, OCH_2), 1.73-1.66 (m, 4 H, CH_2), 0.97 (t, J = 7.34 Hz, 6 H, CH_3) ppm; ^{13}C NMR (125.75 MHz, DMSO-d_6): δ 166.89, 150.43, 125.43, 115.49, 70.57, 22.12, 10.39 ppm.

2,5-Dibutoxy-1,4-benzenedicarboxylic acid (H_2(C4-bdc), ^{1}H NMR (500 MHz, DMSO-d_6): δ 12.88 (s, 2 H, COO–H), 7.26 (s, 2 H, Ar–H), 3.98 (t, J = 6.4 Hz, 4 H, OCH_2), 1.69-1.63 (m, 4 H, CH_2), 1.47-1.40 (m, 4 H, CH_2), 0.91 (t, J = 7.3 Hz, 6 H, CH_3) ppm; ^{13}C NMR (125.75 MHz, DMSO-d_6): δ 166.91, 150.41, 125.44, 115.43, 68.80, 30.81, 18.63, 13.71 ppm.

2,5-Dipentoxy-1,4-benzenedicarboxylic acid (H_2(C5-bdc), ^{1}H NMR (600 MHz, DMSO-d_6): δ 12.89 (s, 2 H, COO–H), 7.25 (s, 2 H, Ar–H), 3.97 (t, J = 6.2 Hz, 4 H, OCH_2), 1.70–1.65 (m, 4 H, CH_2), 1.42–1.37 (m, 4 H, CH_2), 1.36–1.29 (m, 4 H, CH_2), 0.88 (t, J = 7.0 Hz, 6 H, CH_3) ppm; ^{13}C NMR (150.94 MHz, DMSO-d_6): δ 166.84, 150.41, 125.48, 115.49, 69.13, 28.36, 27.53, 21.79, 13.91 ppm.

2,5-Dihexyloxy-1,4-benzenedicarboxylic acid (H_2(C6-bdc), ^{1}H NMR (600 MHz, DMSO-d_6): δ 12.91 (s, 2 H, COO–H), 7.25 (s, 2 H, Ar–H), 3.97 (t, J = 6.4 Hz, 4 H, OCH_2), 1.69-1.64 (m, 4 H, CH_2), 1.44-1.39 (m, 4 H, CH_2), 1.33-1.25 (m, 8 H, CH_2), 0.87 (t, J = 7.0 Hz, 6 H, CH_3) ppm; ^{13}C NMR (150.94 MHz, DMSO-d_6): δ 166.83, 150.41, 125.45, 115.49, 69.12, 30.89, 28.63, 24.97, 22.04, 13.87 ppm.

2,5-Diheptyloxy-1,4-benzenedicarboxylic acid (H_2(C7-bdc), ^{1}H NMR (600 MHz, DMSO-*d*$_6$): δ 12.91 (s, 2 H, COO–H), 7.24 (s, 2 H, Ar–H), 3.96 (t, *J* = 6.4 Hz, 4 H, OCH_2), 1.69-1.64 (m, 4 H, CH_2), 1.44-1.38 (m, 4 H, CH_2), 1.33-1.22 (m, 12 H, CH_2), 0.86 (t, *J* = 6.8 Hz, 6 H, CH_3) ppm; ^{13}C NMR (150.94 MHz, DMSO-*d*$_6$): δ 166.85, 150.41, 125.54, 115.49, 69.12, 31.24, 28.69, 28.35, 25.28, 22.01, 13.95 ppm.

2,5-Dioctyloxy-1,4-benzenedicarboxylic acid (H_2(C8-bdc), ^{1}H NMR (600 MHz, DMSO-*d*$_6$): δ 7.11 (s, 2 H, Ar–H), 3.92 (t, *J* = 6.4 Hz, 4 H, OCH_2), 1.67-1.63 (m, 4 H, CH_2), 1.41-1.37 (m, 4 H, CH_2), 1.31-1.22 (m, 16 H, CH_2), 0.85 (t, *J* = 7.0 Hz, 6 H, CH_3) ppm; ^{13}C NMR (150.94 MHz, DMSO-*d*$_6$): δ 167.82, 150.71, 127.14, 115.76, 69.63, 31.72, 29.27, 29.21, 29.17, 25.86, 22.58, 14.43 ppm.

Supplementary Methods 1.2 – Synthesis of 3,3'-Dioctyloxy-(1,1'-Biphenyl)-4,4'-dicarboxylic acid, H_2(C8-bpdc)

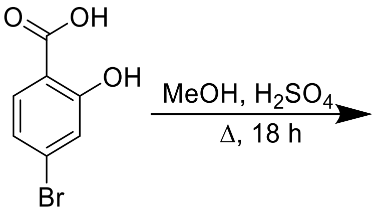

4-bromo-2-hydroxybenzoic acid (23.8 g, 0.109 mol, 1 eq.) was dissolved in methanol (300 mL) and conc. sulfuric acid (8 mL) was added dropwise. The mixture was refluxed overnight. The solvent was evaporated and water (250 mL) was added. The resulting solid was filtered and washed with water multiple times. Recrystallization from methanol gave methyl 4-bromo-2-hydroxybenzoate (13.847 g, 0.059 mmol, 55%) as a white solid.

4-Bromo-2-hydroxybenzoate, 1H NMR (700 MHz, $CDCl_3$): δ 10.82 (s, 1H), 7.68 (d, *J* = 8.5 Hz, 1H), 7.18 (d, *J* = 1.9 Hz, 1H), 7.02 (dd, *J* = 8.5, 1.9 Hz, 1H), 3.95 (s, 3H) ppm; ^{13}C NMR (101 MHz, $CDCl_3$): δ 170.2, 162.1, 131.0, 130.0, 122.8, 120.9, 111.5, 52.6 ppm.

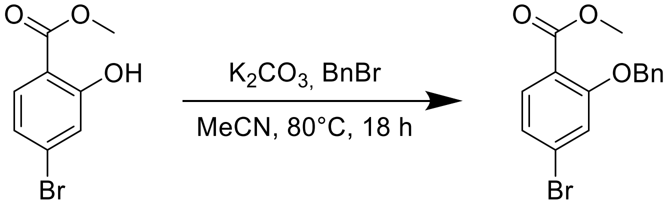

The synthesis was inspired by a procedure from the literature[2]. Methyl 4-bromo-2-hydroxybenzoate (7 g, 30.3 mmol, 1 eq.) and potassium carbonate (8.37 g, 60.59 mmol, 2 eq.) were added to acetonitrile (75 mL). Benzyl bromide (3.6 mL, 30.30 mmol, 1 eq.) were added dropwise and the mixture was refluxed overnight. The solids were filtered, and the

solvents removed from the filtrate to give methyl 2-(benzyloxy)-4-bromobenzoate (9.4 g, 29.2 mmol, 97%) as a clear oil.

Methyl 2-(benzyloxy)-4-bromobenzoate, ^{1}H NMR (400 MHz, $CDCl_3$): δ 7.71 (d, J = 8.3 Hz, 1H), 7.49 (ddt, J = 7.5, 1.4, 0.7 Hz, 2H), 7.40 (ddd, J = 7.6, 6.8, 1.3 Hz, 2H), 7.36 – 7.30 (m, 1H), 7.19 (d, J = 1.8 Hz, 1H), 7.15 (dd, J = 8.3, 1.7 Hz, 1H), 5.17 (s, 2H), 3.89 (s, 3H) ppm. ^{13}C NMR (101 MHz, $CDCl_3$): δ 166.1, 158.8, 136.1, 133.1, 128.7, 128.1, 127.6, 126.9, 123.9, 119.7, 117.4, 71.0, 52.3 ppm.

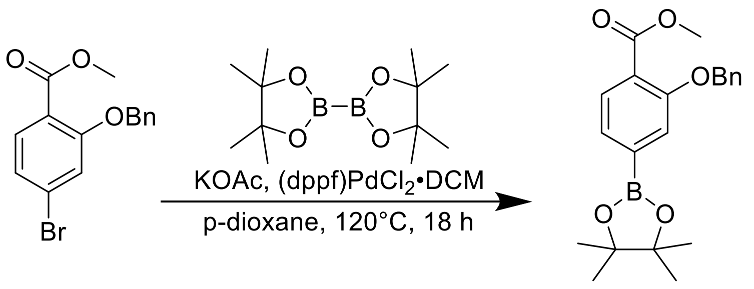

The synthesis was inspired by a procedure from the literature[2]. Under argon atmosphere methyl 2-(benzyloxy)-4-bromobenzoate (9.4 g, 29.2 mmol, 1 eq.), Bis(pinacolato)diboron (8.15 g, 32.1 mmol, 1.1 eq.) and potassium acetate (8.6 g, 87.6 mmol, 3 eq.) were suspended in *p*-dioxane and degassed with argon for 15 minutes. Then [1,1′- bis(diphenylphosphino)ferrocene]dichloropalladium(II) · dichloromethane ((dppf)$PdCl_2$ · DCM, 0.48 g, 0.58 mmol, 0.02 eq.) was added and the mixture was heated to 120 °C overnight. After cooling to room temperature, the mixture was filtered over a plug of Cellite® and the solids were washed with EtOAc. After removing the solvents under reduced pressure 100 mL EtOAc and 2 spoons charcoal were added. The suspension was refluxed for 15 minutes before removing the solids and evaporating the solvents. The resulting solid was recrystallized from hexane giving methyl 2-(benzyloxy)-4-(4,4,5,5-tetramethyl-1,3,2-dioxaborolan-2-yl)benzoate (6.3 g, 17 mmol, 59%) as a light brown solid.

^{1}H NMR (400 MHz, $CDCl_3$): δ 7.73 (d, J = 7.6 Hz, 1H), 7.48 – 7.44 (m, 2H), 7.42 – 7.29 (m, 4H), 7.27 – 7.21 (m, 1H), 5.15 (s, 2H), 3.83 (s, 3H), 1.29 (s, 12H) ppm.^{13}C NMR (101 MHz, $CDCl_3$): δ 167.0, 157.5, 137.0, 131.0, 128.6, 127.8, 127.1, 127.0, 123.3, 119.5, 84.3, 70.8, 52.1, 25.0 ppm.

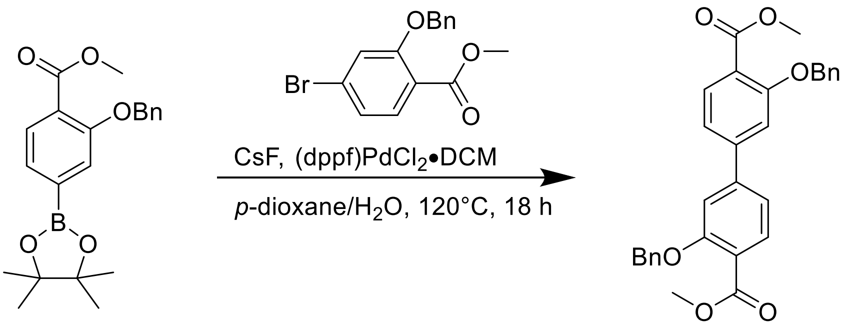

The synthesis was inspired by a procedure from the literature[2]. Under argon atmosphere, methyl 2-(benzyloxy)-4-bromobenzoate (5 g, 15.6 mmol, 1 eq.), methyl 2-(benzyloxy)-4-(4,4,5,5-tetramethyl-1,3,2-dioxaborolan-2-yl)benzoate (6.3 g, 17.1 mmol, 1.1 eq.) and CsF (7.1 g, 46.7 mmol, 3 eq.) were dissolved in a *p*-dioxane/water mixture (2:1, 120 mL). The mixture was degassed with argon for overall 15 minutes, whereas after 10 minutes of degassing (dppf)$PdCl_2$ · DCM (1 g, 1.3 mmol, 0.05 eq.) was added. The mixture was stirred at 80 °C overnight. Water (100 mL) was added and the mixture subsequently cooled to 4 °C. The resulting grey solid was filtered off over an approx. 1 cm thick layer of Cellite® and then washed with cold distilled water. The wash solution was disposed off and the Cellite® filter subsequently washed with DCM dissolving the grey solid. The solvents were then removed under reduced pressure, resulting in a grey-brown residue. The residue was washed with cold EtOAc and dried under dynamic vacuum. Dimethyl 3,3'-bis(benzyloxy)-[1,1'-biphenyl]-4,4'-dicarboxylate (6.2 g, 12.8 mmol, 82%) was obtained as a grey solid.

Dimethyl 3,3'-bis(benzyloxy)-[1,1'-biphenyl]-4,4'-dicarboxylate, ^1H NMR (400 MHz, $CDCl_3$): δ 7.91 (d, J = 8.1 Hz, 2H), 7.55 – 7.50 (m, 4H), 7.44 – 7.38 (m, 4H), 7.37 – 7.30 (m, 2H), 7.16 (dd, J = 8.0, 1.6 Hz, 2H), 7.09 (d, J = 1.6 Hz, 2H), 5.23 (s, 4H), 3.93 (s, 6H) ppm. ^{13}C NMR (101 MHz, $CDCl_3$): δ 166.5, 158.6, 145.4, 136.7, 132.5, 128.7, 128.0, 127.0, 120.3, 119.6, 113.1, 70.9, 52.2 ppm.

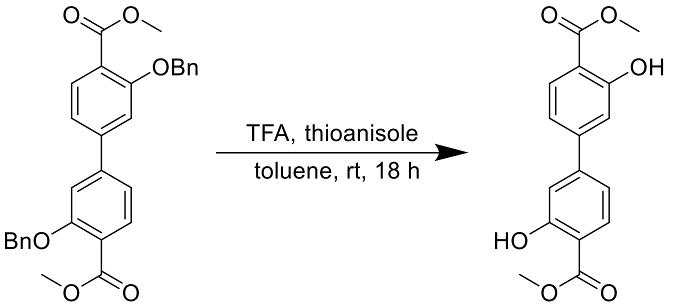

The synthesis was inspired by a procedure from the literature[3]. Under argon atmosphere, dimethyl 3,3'-dihydroxy-[1,1'-biphenyl]-4,4'-dicarboxylate (5.67 g, 11.8 mmol, 1 eq.), trifluoroacetic acid (136.6 ml, 1.76 mol, 150 eq.) and thioanisole (8.3 mL, 70.6 mmol, 6 eq.) were dissolved in toluene (500 mL) and stirred at room temperature overnight. The solution was transferred to a separation funnel and distilled water (800 mL) was added. The aqueous phase was washed with DCM (3 x 200 mL). The combined organic phases were dried with $MgSO_4$ and the solvents removed under reduced pressure, upon which a grey suspension

was obtained. The solids were filtered off and washed with Et_2O. Drying under dynamic vacuum yielded dimethyl 3,3'-dihydroxy-[1,1'-biphenyl]-4,4'-dicarboxylate (2.53 g, 8.3 mmol, 71%).

Dimethyl 3,3'-dihydroxy-[1,1'-biphenyl]-4,4'-dicarboxylate, ^1H NMR (500 MHz, $CDCl_3$): δ 10.8 (s, 2H), 7.90 (d, *J* = 8.3 Hz, 2H), 7.22 (d, *J* = 1.8 Hz, 2H), 7.13 (dd, *J* = 8.3, 1.8 Hz, 2H), 3.98 (s, 6H) ppm. ^{13}C NMR (126 MHz, $CDCl_3$): δ 170.4, 161.9, 146.9, 130.5, 118.3, 116.2, 112.2, 52.5 ppm.

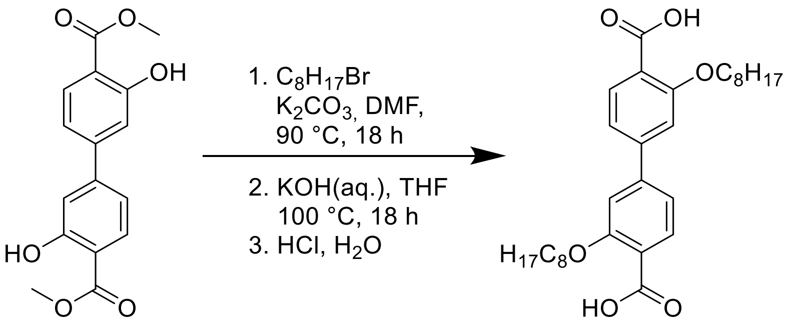

The synthesis was inspired by a procedure from the literature[4]. Dimethyl 3,3'-dihydroxy-[1,1'-biphenyl]-4,4'-dicarboxylate (300 mg, 0.99 mmol, 1 eq.) and K_2CO_3 (686 mg, 4.96 mmol, 5 eq.) were suspended in DMF (20 mL). *n*-Octylbromide (0.98 g, 4.96 mmol, 5 eq.) was added slowly and the mixture was stirred at 90 °C overnight. After cooling to room temperature, the solids were filtered off and the solvents were removed under reduced pressure. For saponification of the ester groups an aqueous solution of KOH (10 wt%, 30 mL) and 15 mL THF was added. The mixture was stirred at 100 °C overnight. After cooling down, an aqueous solution of HCl (10%) was added dropwise, until a solid precipitated (pH = 1). The solid was filtered off and washed with distilled water. Drying under dynamic vacuum gave 3,3'-dioctyloxy-(1,1'-biphenyl)-4,4'-dicarboxylic acid as a white powder in quantitative yield (99%).

3,3'-Dioctyloxy-(1,1'-Biphenyl)-4,4'-dicarboxylic acid, ^1H NMR (400 MHz, DMSO-d_6): δ 12.64 (s, 2H), 7.70 (d, *J* = 7.9 Hz, 2H), 7.36 – 7.29 (m, 4H), 4.15 (t, *J* = 6.4 Hz, 4H), 1.79 – 1.66 (m, 4H), 1.46 (dq, *J* = 9.4, 6.7 Hz, 4H), 1.35 – 1.22 (m, 16H), 0.89 – 0.83 (m, 6H) ppm. ^{13}C NMR (101 MHz, DMSO-d_6): δ 167.2, 157.8, 143.7, 131.1, 121.4, 118.7, 112.0, 68.3, 31.2, 28.7, 28.7, 28.6, 25.4, 22.1, 13.9 ppm.

Supplementary Methods 1.3 – Synthesis of 1,3,5-tris(3'-octyloxy-4'-carboxyphenyl)benzene, H_3(C8-btb)

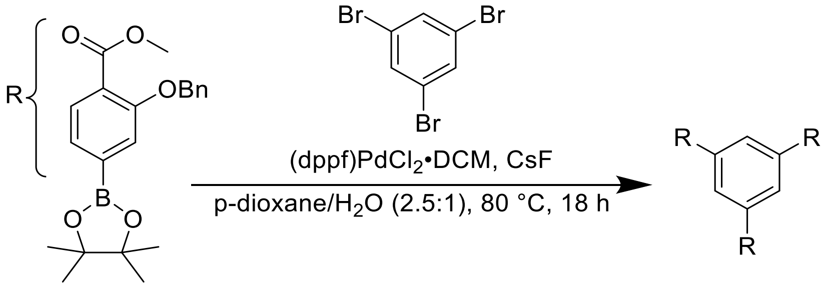

Under argon atmosphere 1,3,5-tribromobenzene (1.66 g, 5.26 mmol, 1 eq.), methyl 2-(benzyloxy)-4-(4,4,5,5-tetramethyl-1,3,2-dioxaborolan-2-yl)benzoate (6.2 g, 16.84 mmol, 3.2 eq.) and caesium fluoride (5 g, 33.15 mmol, 6.3 eq.) were suspended in a *p*-dioxane/water-mixture (120 mL/48 mL) and degassed with argon for 15 minutes. Then [1,1′- bis(diphenylphosphino)ferrocene]dichloropalladium(II) · DCM (0.34 g, 0.42 mmol, 0.1 eq.) was added and the mixture was heated to 80 °C overnight. After cooling to room temperature water (100 mL) was added and the mixture was filtered over a plug of Cellite®. The solids were washed with water and cold EtOAc. Then, the filter was washed with DCM and collected separately from the previous washing solvents. The solvents were removed under reduced pressure to give Dimethyl 3,3''-bis(benzyloxy)-5'-(3-(benzyloxy)-4-(methoxycarbonyl)phenyl)-[1,1':3',1''-terphenyl]-4,4''-dicarboxylate (3.32 g, 4.16 mmol, 79%) as a light grey solid.

Dimethyl 3,3''-bis(benzyloxy)-5'-(3-(benzyloxy)-4-(methoxycarbonyl)phenyl)-[1,1':3',1''-terphenyl]-4,4''-dicarboxylate, ^{1}H NMR (400 MHz, DMSO-d_6) δ 8.02 (s, 3H), 7.86 (d, J = 8.0 Hz, 3H), 7.67 (d, J = 1.7 Hz, 3H), 7.55 (ddd, J = 7.7, 6.2, 1.6 Hz, 9H), 7.44 – 7.38 (m, 6H), 7.34 – 7.29 (m, 3H), 5.42 (s, 6H), 3.85 (s, 9H). ^{13}C NMR (101 MHz, DMSO-d_6) δ 168.5, 165.8, 161.0, 157.8, 144.9, 143.0, 140.7, 137.0, 131.5, 128.3, 127.6, 126.9, 119.4, 69.8, 51.9.

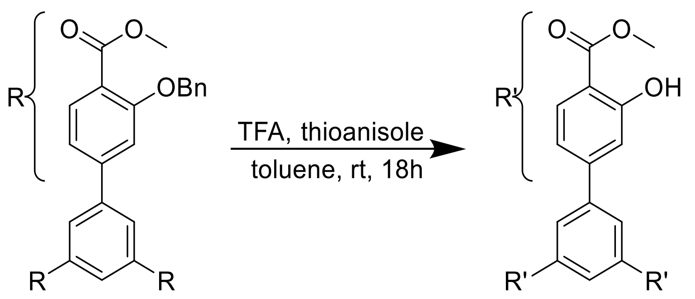

The synthesis was inspired by a procedure from the literature[3]. Under argon atmosphere dimethyl 3,3''-bis(benzyloxy)-5'-(3-(benzyloxy)-4-(methoxycarbonyl)phenyl)-[1,1':3',1''-terphenyl]-4,4''-dicarboxylate (2.6 g, 3.29 mmol, 1 eq.), thioanisole (3.5 mL, 29.6 mmol, 9 eq.)

and trifluoroacetic acid (38 mL, 493.74 mmol, 150 equiv.) were dissolved in toluene (180 mL) and stirred at room temperature overnight. Then, water (100 mL) was added and the solution was extracted with DCM (3 x 50 mL). The combined organic phases were washed with brine (1x 50 mL) and dried over $MgSO_4$. The solvents were removed under reduced pressure as far as possible. The resulting suspension was heated to 110 °C and toluene was added slowly until everything was dissolved. The solution was cooled to 4 °C for 3 days before filtering off and drying the product Dimethyl 3,3''-dihydroxy-5'-(3-hydroxy-4-(methoxycarbonyl)phenyl)-[1,1':3',1''-terphenyl]-4,4''-dicarboxylate (1.17 g, 2.22 mmol, 67%) as a white solid.

Dimethyl 3,3''-dihydroxy-5'-(3-hydroxy-4-(methoxycarbonyl)phenyl)-[1,1':3',1''-terphenyl]-4,4''-dicarboxylate, ^1H NMR (500 MHz, $CDCl_3$): δ 10.84 (s, 3H), 7.91 (d, J = 8.2 Hz, 3H), 7.80 (s, 3H), 7.27 (d, J = 1.7 Hz, 3H), 7.17 (dd, J = 8.3, 1.8 Hz, 3H), 3.99 (s, 9H) ppm. ^{13}C NMR (126 MHz, $CDCl_3$): δ 170.4, 161.9, 141.1, 130.6, 130.5, 129.0, 126.1, 118.2, 116.0, 52.4 ppm.

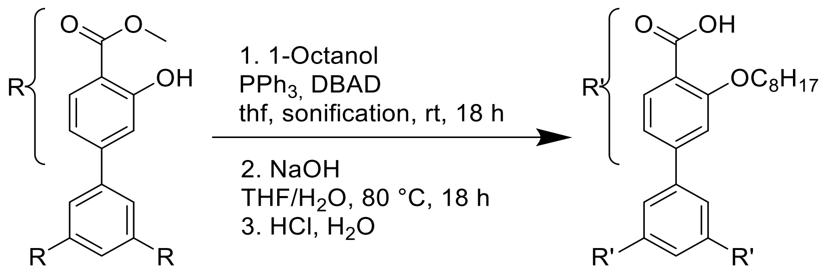

The synthesis was inspired by a procedure from the literature[5]. Under argon atmosphere the starting compound dimethyl 3,3''-dihydroxy-5'-(3-hydroxy-4-(methoxycarbonyl)phenyl)-[1,1':3',1''-terphenyl]-4,4''-dicarboxylate (300 mg, 0.57 mmol, 1 eq.), PPh_3 (522 mg, 1.99 mmol, 3.5 eq.) and di-*tert*-butyl azodiformate (DBAD) (458 mg, 1.99 mmol, 3.5 eq.) were dissolved in THF (8 mL) under sonification. The aliphatic alcohol (1.99 mmol, 3.5 eq.) was added and the mixture sonificated at room temperature for 3 hours. Then sodium hydroxide (40 mL, 10 wt% in water) was added and the solution refluxed overnight. The majority of the organic solvents were removed under reduced pressure giving a clear colorless solution. The solution was extracted with EtOAc (3x 50 mL) and the solvent was removed from the combined organic phases. Sodium hydroxide (20 mL, 10 wt% in water) and THF (20 mL) were added and the mixture was refluxed overnight. The resulting phases were separated, and hydrochloric acid was added to the organic phase while stirring vigorously. The resulting solid was washed with water and dried under vacuum giving the product 1,3,5-tris(3'-octyloxy-4'-carboxyphenyl)benzene (251 mg, 304 mmol, 60%) as a white solid.

1,3,5-tris(3'-octyloxy-4'-carboxyphenyl)benzene, ^1H NMR (400 MHz, $CDCl_3$): δ 10.95 (s, 3H), 8.31 (d, *J* = 8.3 Hz, 3H), 7.81 (s, 3H), 7.46 – 7.40 (m, 3H), 7.30 – 7.28 (m, *J* = 1.5 Hz, 3H), 4.36 (t, *J* = 6.4 Hz, 6H), 1.97 (tt, *J* = 7.3 Hz, 6.4 Hz, 6H), 1.52 (tt, *J* = 7.3 Hz, 6.9 Hz, 6H), 1.44 – 1.24 (m, 24H), 0.89 (t, *J* = 6.9 Hz, 9H) ppm. ^{13}C NMR (101 MHz, $CDCl_3$): δ 165.0, 158.0, 147.2, 141.6, 134.5, 126.6, 121.2, 117.1, 111.6, 70.7, 31.7, 29.2, 29.1, 29.0, 25.9, 22.6, 14.1 ppm.

Supplementary Methods 1.4 – MOF-5-CX – Synthesis and activation

All MOF-5-**CX** materials were prepared under the same reaction conditions. In a typical synthesis 0.756 g $Zn(NO_3)_2 \cdot 4\ H_2O$ (2.89 mmol) and the appropriate H_2(CX-bdc) linker precursor (0.666 mmol) were weighted in an 100 ml screw cap vial and 50 ml DMF were added. The reaction mixture was sonicated until the components were completely dissolved, giving a clear solution, which was heated at 100 °C for 48 h. After cooling to room temperature, the mother liquor was decanted and the colourless cubic crystals were immersed in fresh DMF (3 × 50 ml) over several hours. Analytics referred to as *'as-synthesized'* were undertaken at this point. For activation of the MOFs, the DMF was decanted and replaced by DCM (50 ml) and the crystals were kept in DCM overnight. This procedure was repeated two times. Following, the solvent was decanted and the crystals dried in dynamic vacuum (10^{-3} mbar) at 100 °C for 20 h. The dried MOFs were stored in a Schlenk tube under argon atmosphere prior to sample characterization of the *'dried'* materials.

The amount of incorporated DMF molecules in the pores of the *as*-MOF-5-CX was determined by solution ^1H NMR spectroscopy. In order to do so, DMF-washed crystals of *as*-MOF-5-**CX** were shortly put on a filter paper to remove excess DMF from the surface of the crystals. These crystals were then digested in a mixture of DMSO-*d6* and DCl/D_2O for ^1H NMR spectroscopy. Resulting amounts of incorporated DMF molecules per repeating unit are given below.

Supplementary Table 1: Chemical composition (including contained DMF) of as-MOF-5-CX as obtained from ^1H NMR spectroscopy of digested samples of the as-MOF-5-CX.

**CX**	Chemical composition
C2	$[Zn_4O(C2\text{-bdc})_3]\cdot 7.5DMF$
C3	$[Zn_4O(C3\text{-bdc})_3]\cdot 6.6DMF$
C4	$[Zn_4O(C4\text{-bdc})_3]\cdot 5.1DMF$
C5	$[Zn_4O(C5\text{-bdc})_3]\cdot 3.9DMF$
C6	$[Zn_4O(C6\text{-bdc})_3]\cdot 3.6DMF$
C7	$[Zn_4O(C7\text{-bdc})_3]\cdot 2.4DMF$
C8	$[Zn_4O(C8\text{-bdc})_3]\cdot 1.5DMF$

Supplementary Methods 1.5 - MOF-5-CX reinfiltration

For the reinfiltration of a MOF utilizing DMF, a glass weight boat was loaded with a powdered sample of the dried MOF of choice and another weight boat with a small amount of DMF (~ 1 ml). Both weight boats were placed in a Schlenk tube. The Schlenk tube was evacuated for a short time, sealed under reduced pressure and heated at 65 °C for 16 h.

Re-infiltration experiments using n-octane were performed by immersing dried crystals of the MOF in ~2 ml *n*-octane in a 12 ml screw cap vial, followed by heating at 65 °C for 16 h. Analytics were performed immediately after cooling to room temperature.

Supplementary Methods 1.6 - MOF-5-CX activation from *n*-hexane

Activation from *n*-hexane was performed according to the procedure described under **Supplementary Methods 1.4** to the point where the DCM exchange is completed. Following the DCM was exchanged for *n*-hexane (3 × 50 ml) and kept overnight. The *n*-hexane was removed under dynamic vacuum (10^{-2} mbar) at 100 °C for 24 h. Dried MOFs were kept in a Schlenk tube filled with Argon.

Supplementary Methods 1.7 - IRMOF-10-C8 – Synthesis and activation

Based on a published procedure[6], in a typical synthesis 0.115 g $Zn(NO_3)_2 \cdot 4\ H_2O$ (0.44 mmol) and 0.04 g 3,3'-dioctyloxy-(1,1'-biphenyl)-4,4'-dicarboxylic acid (0.08 mmol) were dissolved in 12 ml DEF and heated at 100 °C for 48 h. After cooling to room temperature, the mother liquor was washed with DMF (3 × 15 ml). The activation procedure was inspired by a published freeze drying method using benzene[7]. The DMF was exchanged for benzene (3 × 15 ml), kept overnight and exchanged once again. Following this, the benzene was frozen at 0 °C and sublimed at a pressure of 10^{-3} mbar for 2 h. Afterwards the sample was heated at 80 °C in vacuo for 15 h and then heated at 110 °C for 6 h. After cooling to room temperature, the sample was stored in a Schlenk flask under Argon atmosphere prior to sample analysis of the *'dried'* material.

Supplementary Methods 1.8 - MOF-177-C8 – Synthesis and activation

Based on a published procedure[8], the compound was prepared from a solution of 0.088 g $Zn(NO_3)_2 \cdot 4\ H_2O$ (0.34 mmol) and 0.037 g 1,3,5-tris(3'-octyloxy-4'-carboxyphenyl)benzene (0.05 mmol) in 3.75/3.75/0.75 ml DMF/NMP/EtOH. This solution was heated at 100 °C for 48 h. The resulting crystals were washed with DMF (3 × 10 ml). The dried material was obtained via the same procedure as IRMOF-10-C8.

Supplementary Methods 1.9 - IRMOF-10-C8/MOF-177-C8 reinfiltration

A suspension of a ground sample of the corresponding MOF in a small amount of DEF (< 0.5 ml) was prepared. From this suspension, samples for PXRD and IR spectroscopy were prepared.

Supplementary Methods 1.10 - MOF-5 – Synthesis and activation

Based on a published procedure[9], the compound was prepared from a solution of 1.80 g $Zn(NO_3)_2{\cdot}6H_2O$ (6.1 mmol) and 0.33 g 1,4-benzenedicarboxylic acid (2.0 mmol) in 50 ml

freshly distilled DEF, which was heated at 80 °C for 3 d. The resulting crystals were washed with dry DMF (3 × 50 ml, dried over 4 Å molecular sieve) and the solvent was exchanged for DCM (3 × 50 ml) with 5 h between each exchanging step. The crystals were filtered off and dried in vacuo (10^{-2} mbar) at 100 °C for 20 h. The dried samples were kept in a Schlenk tube under argon atmosphere

Supplementary Methods 2 - Powder X-ray diffraction (PXRD)

Supplementary Methods 2.1 Profile fitting

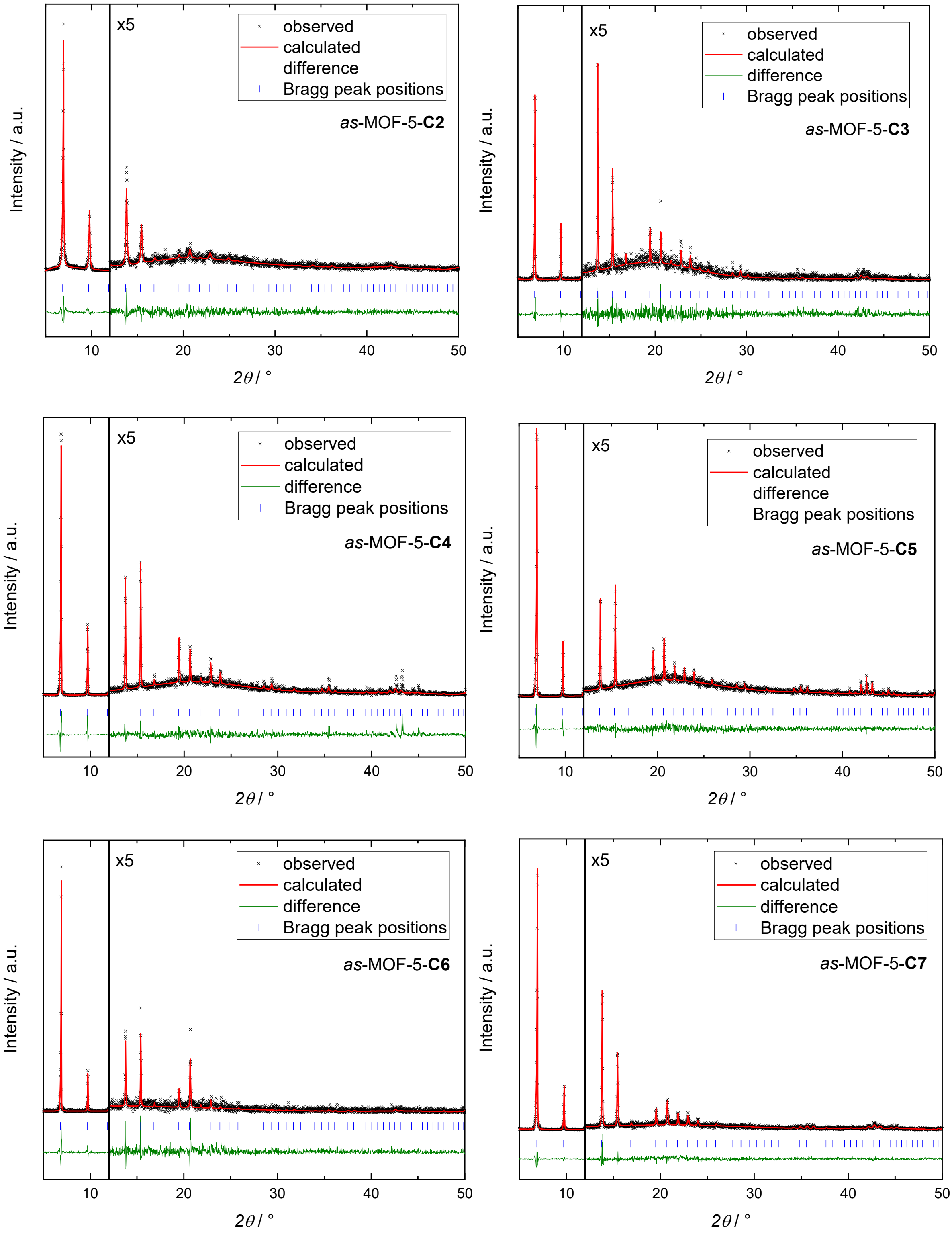

Supplementary Figure 2.1: PXRD patterns with profile fits (Pawley method) of as-synthesized MOF-5-C2 to MOF-5-C7.

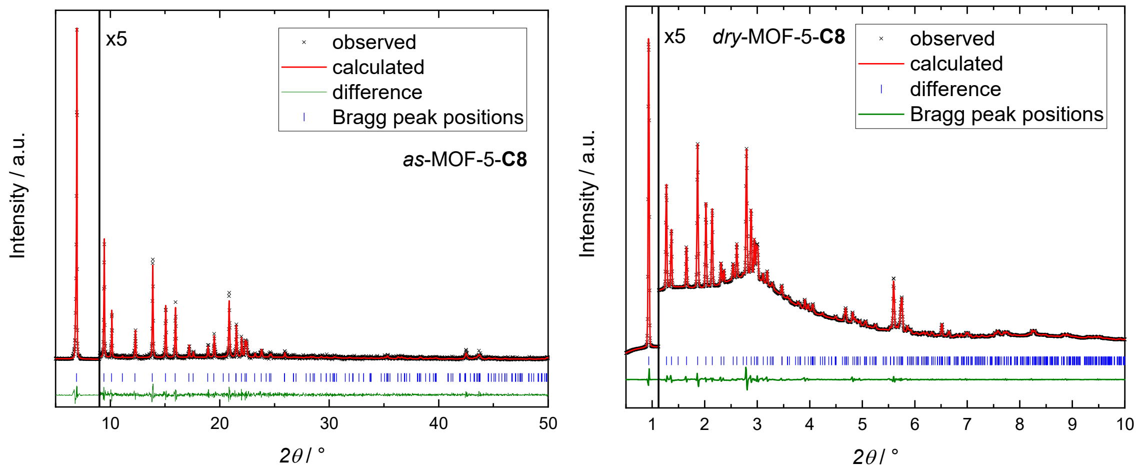

Supplementary Figure 2.2: PXRD patterns with profile fits (Pawley method) of *as-synthesized* and dry-MOF-5-C8. The pattern of dry-MOF-5-C8 was recorded at beamline P02.1 at DESY (Deutsches Elektronen-Synchrotron, Hamburg, Germany) with a monochromatic X-ray beam (λ = 0.207 Å).

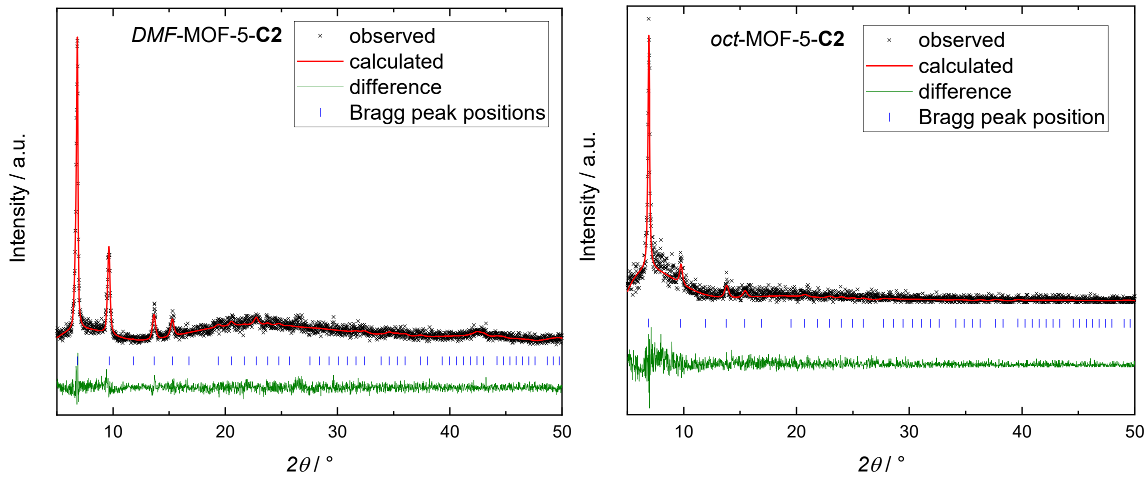

Supplementary Figure 2.3: PXRD patterns with profile fits (Pawley method) of MOF-5-C2 reinfiltrated with DMF (left) or n-octane (right).

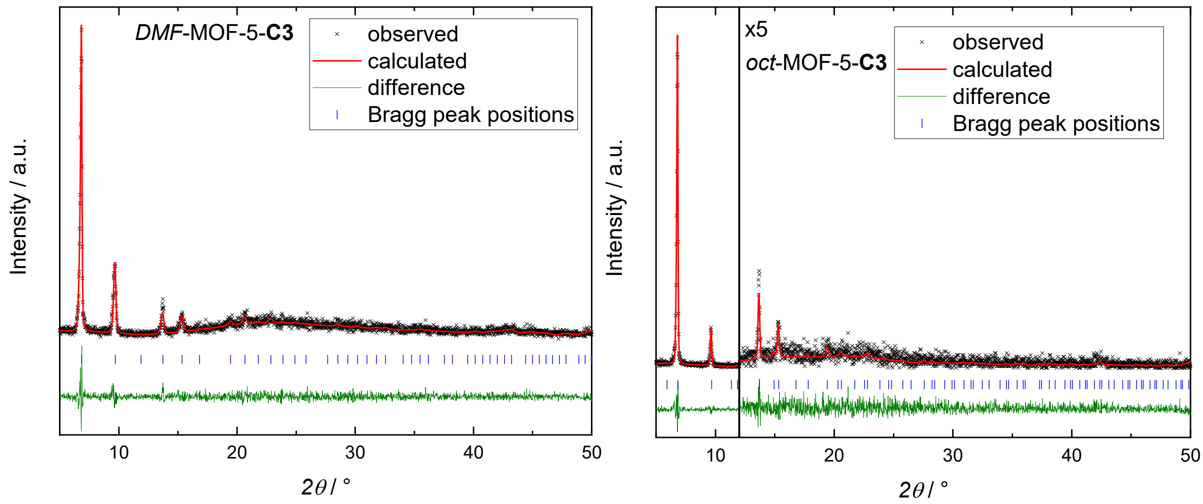

Supplementary Figure 2.4: PXRD patterns with profile fits (Pawley method) of MOF-5-C3 reinfiltrated with DMF (left) or n-octane (right).

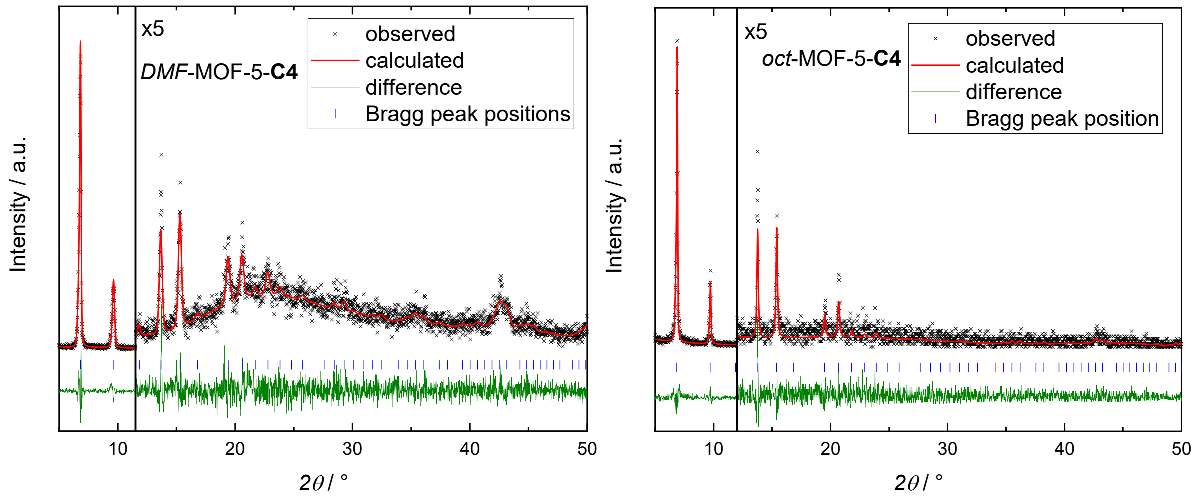

Supplementary Figure 2.5: PXRD patterns with profile fits (Pawley method) of MOF-5-C4 reinfiltrated with DMF (left) or n-octane (right).

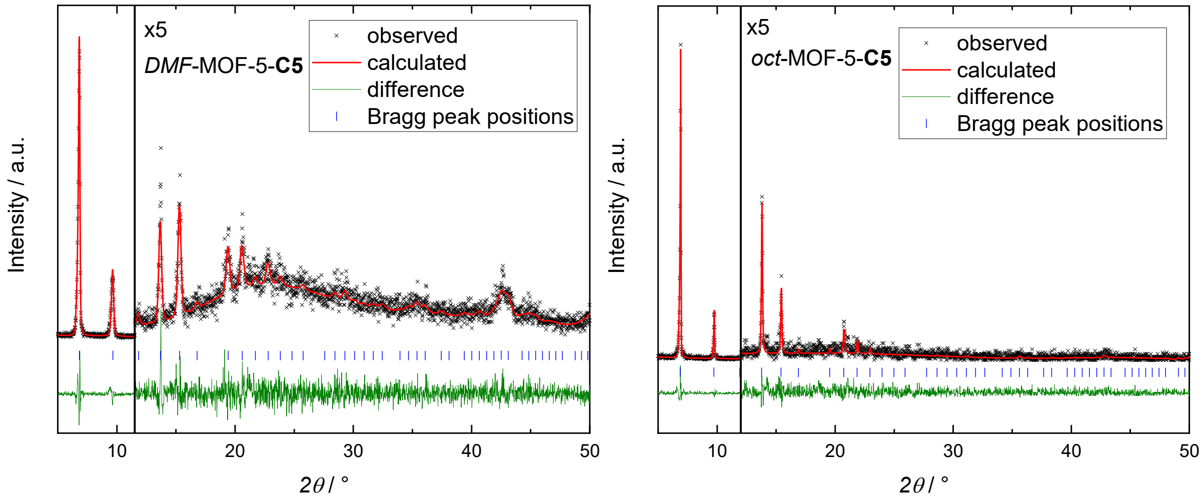

Supplementary Figure 2.6: PXRD patterns with profile fits (Pawley method) of MOF-5-C5 reinfiltrated with DMF (left) or n-octane (right).

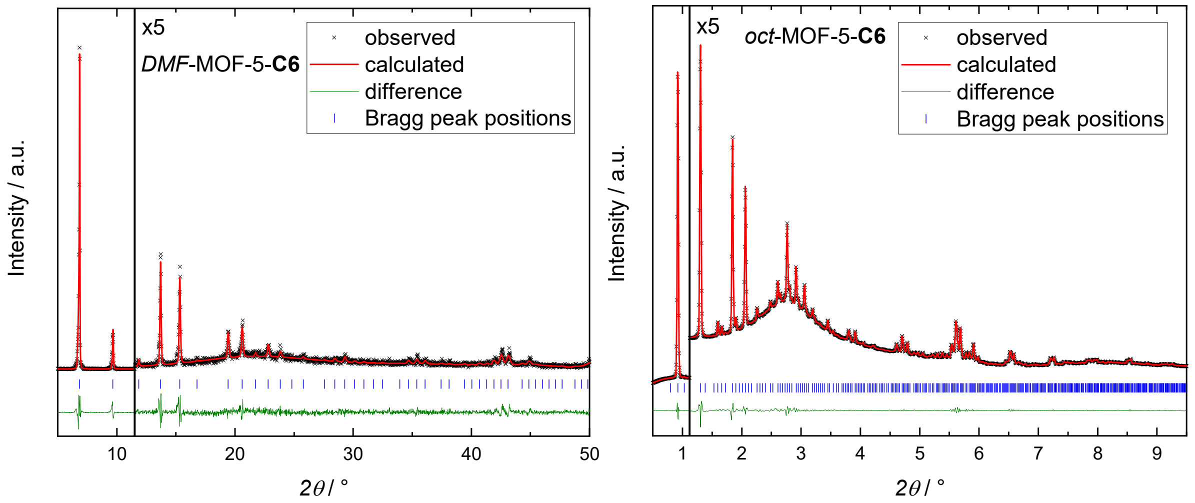

Supplementary Figure 2.7: PXRD patterns with profile fits (Pawley method) of MOF-5-C6 reinfiltrated with DMF (left) or n-octane (right, data collected at P02.1 of DESY, λ = 0.207 Å). In contrast to as- or DMF-MOF-5-C6, which crystallize in a cubic unit cell with a ~ 12.9 Å and $Pm\bar{3}m$ symmetry, oct-MOF-5-C6 was found to crystallize in a cubic unit cell with *a* = 25.7145 Å space group $Pa\bar{3}$ (see Supplementary Methods 3.1, Supplementary Table 4.3).

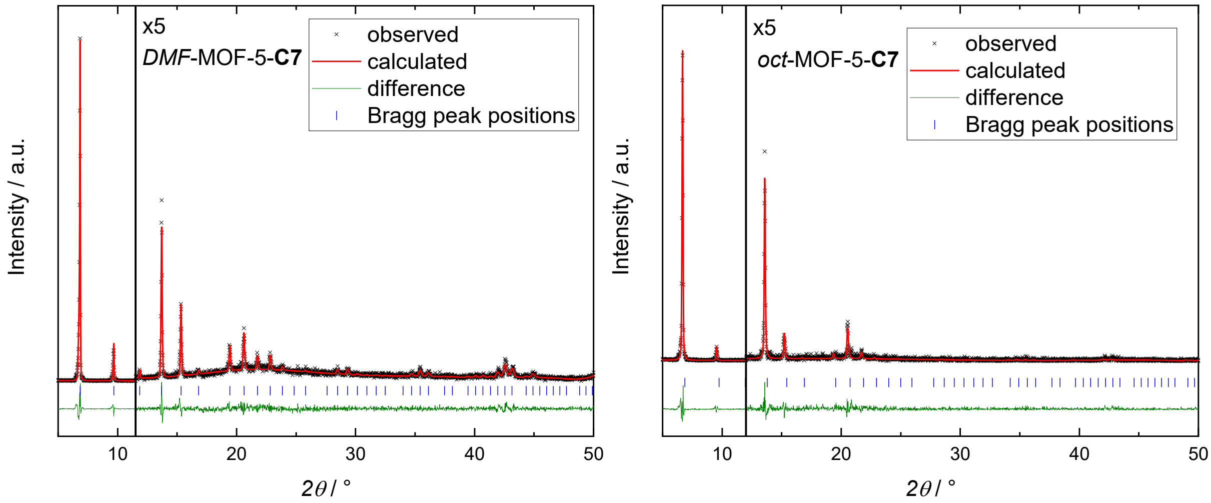

Supplementary Figure 2.8: PXRD patterns with profile fits (Pawley method) of MOF-5-C7 reinfiltrated with DMF (left) or n-octane (right).

Supplementary Table 2.1: Unit cell parameters *a*, *b*, *c* and cell volume *V* determined by profile fitting (Pawley method) of the PXRD patterns and the corresponding R_{wp}, R_{exp} and χ^2 values for the MOF-5-CX in various solvated states.

Compound	as-MOF-5-C2	DMF resolvated MOF-5-C2	oct-MOF-5-C2
crystal system	cubic	cubic	cubic
space group	$Pm\bar{3}m$	$Pm\bar{3}m$	$Pm\bar{3}m$
a / Å	12.913(3)	12.952(10)	12.8604(2)
b / Å	12.913(3)	12.952(10)	12.860(2)
c / Å	12.913(3)	12.952(10)	12.860(2)
α / °	90	90	90
β / °	90	90	90
γ / °	90	90	90
V / Å^3	2153.6(18)	2173(5)	2127.0(12)
R_{wp}	20. 55	18.62	39.84
R_{exp}	16.19	17.12	38.75
χ^2	1.61	1.18	1.06

Compound	as-MOF-5-C3	DMF resolvated MOF-5-C3	oct-MOF-5-C3
crystal system	cubic	cubic	cubic
space group	$Pm\bar{3}m$	$Pm\bar{3}m$	$Fm\bar{3}m$
a / Å	12.934(2)	12.895(7)	25.852(13)
b / Å	12.934(2)	12.895(7)	25.852 (13)
c / Å	12.934(2)	12.895(7)	25.852(13)
α / °	90	90	90
β / °	90	90	90
γ / °	90	90	90
V / Å^3	2163.8(10)	2144(4)	17277(27)
R_{wp}	29.92	21.55	36.65
R_{exp}	26.56	19.77	32.75
χ^2	1.27	1.19	1.25

Compound	as-MOF-5-C4	DMF resolvated MOF-5-C4	oct-MOF-5-C4
crystal system	cubic	cubic	cubic
space group	$Pm\bar{3}m$	$Pm\bar{3}m$	$Pm\bar{3}m$
a / Å	12.9395(13)	12.933(5)	12.889(5)
b / Å	12.9395(13)	12.933(5)	12.889(5)
c / Å	12.9395(13)	12.933(5)	12.889(5)
α / °	90	90	90
β / °	90	90	90
γ / °	90	90	90
V / Å^3	2166.5(7)	2163(2)	2141(2)
R_{wp}	22.72	18.46	40.18
R_{exp}	15.29	16.72	37.00
χ^2	2.21	1.22	1.18

Compound	as-MOF-5-C5	DMF resolvated MOF-5-C5	oct-MOF-5-C5
crystal system	cubic	cubic	cubic
space group	$Pm\bar{3}m$	$Pm\bar{3}m$	$Pm\bar{3}m$
a / Å	12.9202(6)	12.900(2)	12.853(3)
b / Å	12.9202(6)	12.900(2)	12.853(3)
c / Å	12.9202(6)	12.900(2)	12.853(3)
α / °	90	90	90
β / °	90	90	90
γ / °	90	90	90
V / Å^3	2156.8(3)	2146.8(9)	2123(2)
R_{wp}	18.03	19.27	38.40
R_{exp}	14.75	15.38	35.96
χ^2	1.49	1.57	1.14

Compound	as-MOF-5-C6	DMF resolvated MOF-5-C6	oct-MOF-5-C6
crystal system	cubic	cubic	cubic
space group	$Pm\bar{3}m$	$Pm\bar{3}m$	$Pa\bar{3}$
a / Å	12.924(2)	12.9294(14)	25.7329(2)
b / Å	12.924(2)	12.929414)	25.7329(2)
c / Å	12.924(2)	12.9294(14)	25.7329(2)
α / °	90	90	90
β / °	90	90	90
γ / °	90	90	90
V / Å^3	2158.5(11)	2161.4(7)	17039.9(9)
R_{wp}	38.75	19.76	1.74
R_{exp}	33.97	13.62	0.55
χ^2	1.30	2.10	1.52

Compound	as-MOF-5-C7	DMF resolvated MOF-5-C7	oct-MOF-5-C7
crystal system	cubic	cubic	cubic
space group	$Pm\bar{3}m$	$Pm\bar{3}m$	$Pm\bar{3}m$
a / Å	12.8660(12)	12.9182(12)	12.843(2)
b / Å	12.8660(12)	12.9182(12)	12.843(2)
c / Å	12.8660(12)	12.9182(12)	12.843(2)
α / °	90	90	90
β / °	90	90	90
γ / °	90	90	90
V / Å^3	2129.8(6)	2155.8(6)	2118.5(11)
R_{wp}	21.02	18.51	31.62
R_{exp}	16.58	13.74	26.93
χ^2	1.61	1.81	1.38

Compound	as-MOF-5-C8		dry-MOF-5-C8	
crystal system	trigonal	rhombohedral	trigonal	rhombohedral
space group	$R\bar{3}$		$R\bar{3}$	
a / Å	17.485(2)	12.862(2)	17.4115(9)	12.8204(5)
b / Å	17.485(2)	12.862(2)	17.4115(9)	12.8204(5)
c / Å	23.913(2)	12.862(2)	23.870(2)	12.8204(5)
α / °	90	85.639 (8)	90	85.539(5)
β / °	90	85.639 (8)	90	85. 539(5)
γ / °	120	85.639 (8)	120	85. 539(5)
V / Å^3	6331.0(16)	2110.3(5)	6267.1(9)	2089.0(3)
R_{wp}	23.74		1.41	1.41
R_{exp}	18.85		1.09	1.09
χ^2	1.59		1.68	1.68

Supplementary Methods 2.2 - Rietveld refinement of the PXRD pattern of dry-MOF-5-C7

Rietveld refinement was performed using the routines implemented in the TOPAS-*Academic v6* software package[10], peak shapes were fitted using the Thompson-Cox-Hastings profile.[11]

The starting structural model was based on the single crystal structure of rhombohedral as-MOF-5-C8, which was modified by reducing the linker carbon chain lengths from C8 to C7. A profile fit (Pawley method) gave unit cell and background parameters, which were taken over into the refinement using the Rietveld method.

The obtained model was refined against synchrotron powder diffraction data (Deutsches Elektronen Synchrotron, DESY, beamline P02.1, λ = 0.207 Å) in a 2θ range from 0.5° to 12.3°. In initial refinement steps, only zinc and oxygen atom positions were refined applying bond distance (Zn–O, Zn–Zn) and angle (Zn–O–Zn, O–Zn–O) restraints according to values of the crystal structure of as-MOF-5-C8. The organic linker (except for the carboxylate-oxygen atoms) of the framework was described as a rigid body (using a Z-matrix expression) of which the carbon atom positions (except for those belonging to the alkyl groups) were refined previously using distance and angle restraints as before. Additionally, a dummy atom was placed on the centre of inversion located at the centre of the linker's phenyl ring to further restrain the associated carbon atom positions using distance restraints. Within later cycles, all alkyl carbon atom positions were refined by variation of their corresponding Z-matrix parameters. At this, C–C bond distances and angles were restrained to an interval of 1.51-1.55 Å and 108-112°, respectively, while the O(ether)–C distance was limited to range from 1.28 to 1.36 Å. Further, translational and torsional movement of the rigid body was enabled during this stage of the refinement. Displacement parameters (b_{eq}) were refined as three groups of equal values ((1) zinc atoms, (2) all oxygen and phenyl- and carboxylate carbon atoms, (3) alkyl chain carbon atoms. The final Rietveld plot and resulting crystallographic data are given below.

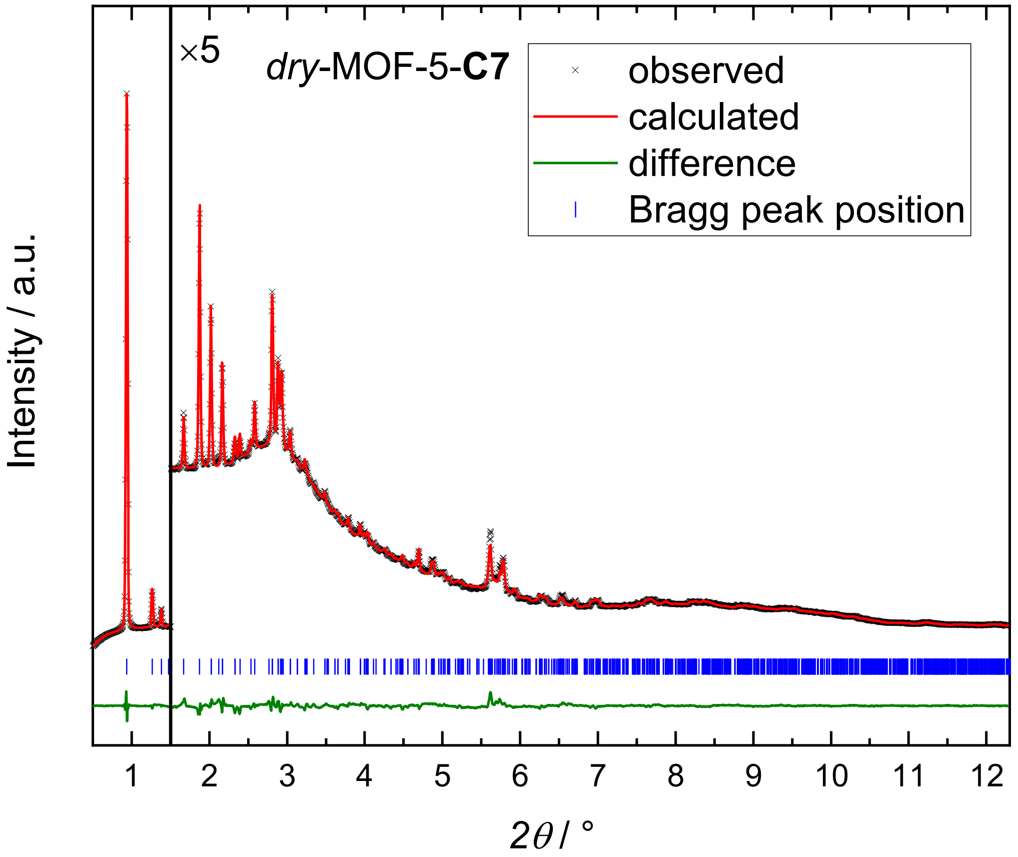

Supplementary Figure 2.9: Final Rietveld fit of PXRD data of dry-MOF-5-C7 (λ = 0.207 Å).

Supplementary Table 2.2: Crystallographic data for the Rietveld refinement of dry-MOF-5-C7 (CCDC deposition number 2040923).

Compound	dry-MOF-5-C7	
Empirical formula	$C_{66}H_{96}O_{19}Zn_4$	
Formula weight	1454.90	
Calclulated density	1.093	
space group	$R\bar{3}$	
Crystal system	trigonal	rhombohedral
a / Å	17.1950(6)	12.7860(5)
b / Å	17.1950(6)	12.7860(5)
c / Å	24.1731(18)	12.7860(5)
α / °	90	84.507(4)
β / °	90	84.507(4)
γ / °	120	84.507(4)
V / Å^3	6189.7(6)	2063.2
Z	3	1
Diffractometer	P02.1, Deutsches Elektronen Synchrotron, Hamburg, Germany	
Temperature / K	300	
Wavelength / Å	0.2073	
No. Reflections	1791	
No. Atoms	19	
No. Restraints	59	
R_p	0.930	
R_{exp}	2.398	
R_{wp}	1.356	
χ^2	0.320	
R_{Bragg}	0.591	

Supplementary Methods 2.3 - Analysis of X-ray scattering data

The first scattering peak (FSP) found for the materials dry-MOF-5-C2 to dry-MOF-5-C6 was fitted using split pseudo-Voigt (C3-C6) or PearsonVII (C2) peak profile functions as implemented in the TOPAS-*academic v6* software package. In this process only the first scattering peak and the corresponding background function were refined. The obtained parameters are given below.

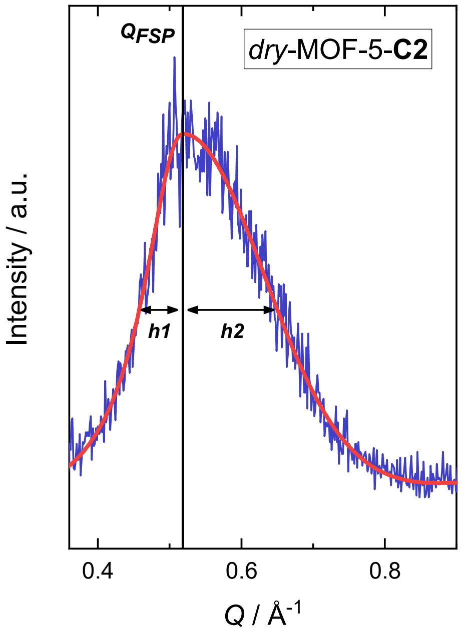

Supplementary Figure 2.10: Legend for the extracted fit parameters. Q_{FSP} is the peak maximum, h1 and h2 are the width at half mean of the left and right composite peak, respectively. The sum of h1 and h2 is the full width half mean (FWHM) of the full peak.

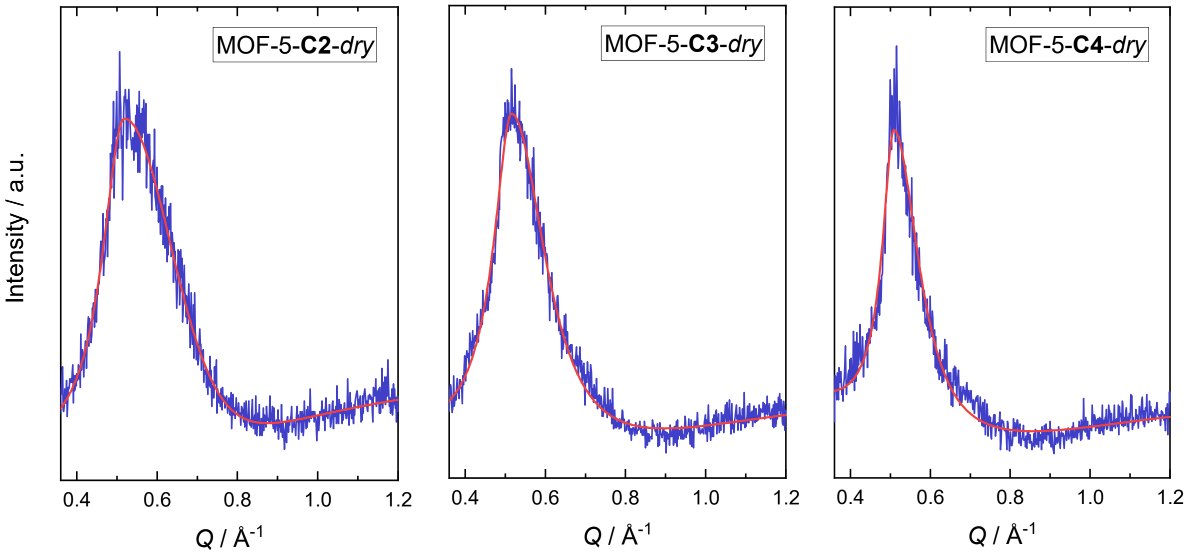

Supplementary Figure 2.11: PXRD pattern with fit of the FSP of dry-MOF-5-C2 to dry-MOF-5-C4.

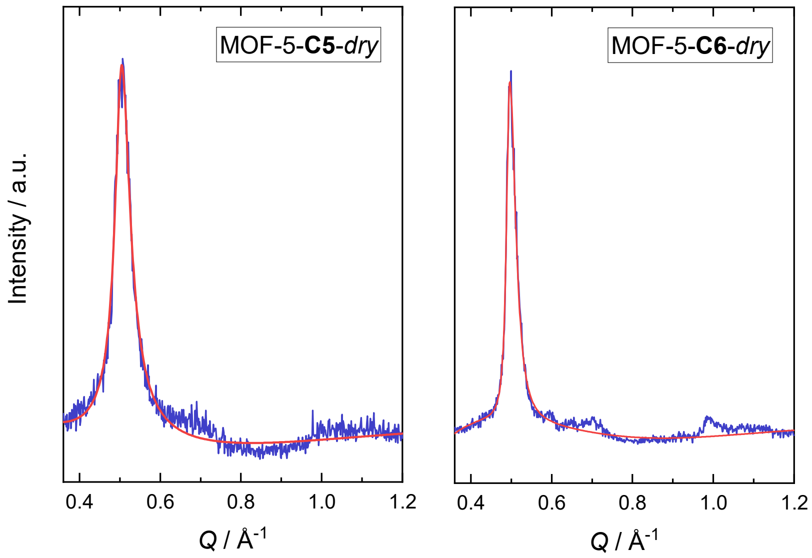

Supplementary Figure 2.12: PXRD pattern with fit of the FSP of dry-MOF-5-C5 and dry-MOF-5-C6.

From the peak maximum Q_{FSP} the corresponding real space distance d_{FSP} is calculated (Bragg equation). d_{FSP} is corresponding to the average Zn_4O–bdc–Zn_4O distance in the non-crystalline/nano-crystalline frameworks and thus also corresponding to the average edge length of the MOF-5-CX cages, we calculated $V_{contracted,dry} = d_{FSP}^3$, the mean volume of one formula unit ($Zn_4O(CX\text{-}bdc)_3$) in the non-crystalline/nano-crystalline contracted phase. This was related to the corresponding unit cell volume of the guest-filled cubic phase to estimate the average volume change upon distortion to dry-MOF-5-CX. Resulting values are given below.

Supplementary Table 2.3: Parameters extracted from fits of the first scattering peak of dry-MOF-5-CX.

**X**	*reciprocal space*				*real space*
	Q_{FSP} / Å^{-1}	*h1* / Å^{-1}	*h2* / Å^{-1}	*FWHM* / Å^{-1}	d_{FSP} / Å
**C2**	0.518	0.0393	0.1338	0.1731	12.12
**C3**	0.515	0.0565	0.0925	0.1490	12.20
**C4**	0.510	0.0333	0.0685	0.1018	12.32
**C5**	0.505	0.0192	0.0262	0.0454	12.43
**C6**	0.496	0.0071	0.0177	0.0248	12.66

Supplementary Table 2.4: Estimated volumes of dry-MOF-5-CX.

**X**	d_{FSP} / Å	$V_{contracted,dry}$ / Å^3	$V_{cubic,as}$ / Å^3	%change
C2	12.12	1780.4	2153.6	-17
C3	12.20	1815.9	2164.3	-16
C4	12.32	1870.0	2168.3	-14
C5	12.43	1920.5	2157.6	-11
C6	12.66	2029.1	2158.3	-6

Supplementary Methods 2.4 - PXRD pattern of dry-MOF-5-C3 activated from n-hexane.

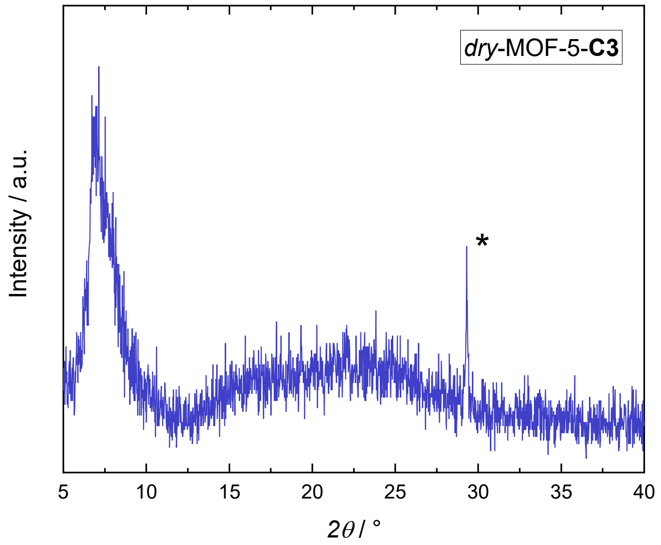

Supplementary Figure 2.13: PXRD pattern of dry-MOF-5-C3 activated from n-hexane (a low surface tension liquid, $\sigma(20°C)$ = 18.35 mN m^{-1})[12]. Even upon activation with the low surface tension liquid, the framework transforms to a non-crystalline distorted structure identical to the same material activated from dichloromethane (a higher surface tension liquid, $\sigma(20°C)$ = 26.50 mN m^{-1})[13]. The influence of surface tension effects on the framework distortion can thus be excluded. The marked reflection (*) stems from the material of the plastic sample holder used to record the pattern.

Supplementary Methods 2.5 - PXRD patterns of IRMOF-10-C8 and MOF-177-C8

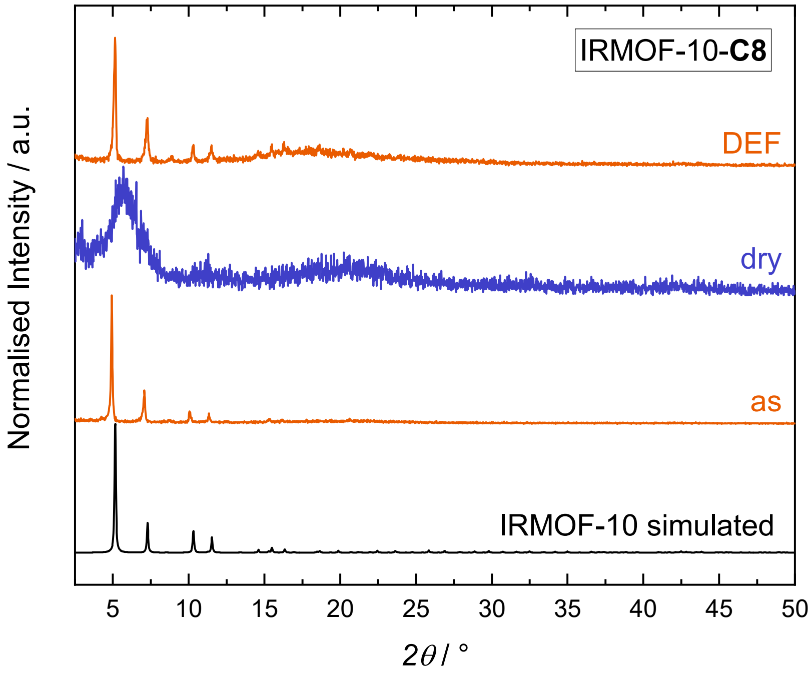

Supplementary Figure 2.14: PXRD patterns of as-IRMOF-10-C8, dry-IRMOF-10-C8 and DEF-IRMOF-10-C8 in comparison with a simulated XRPD pattern of IRMOF-10.[6]

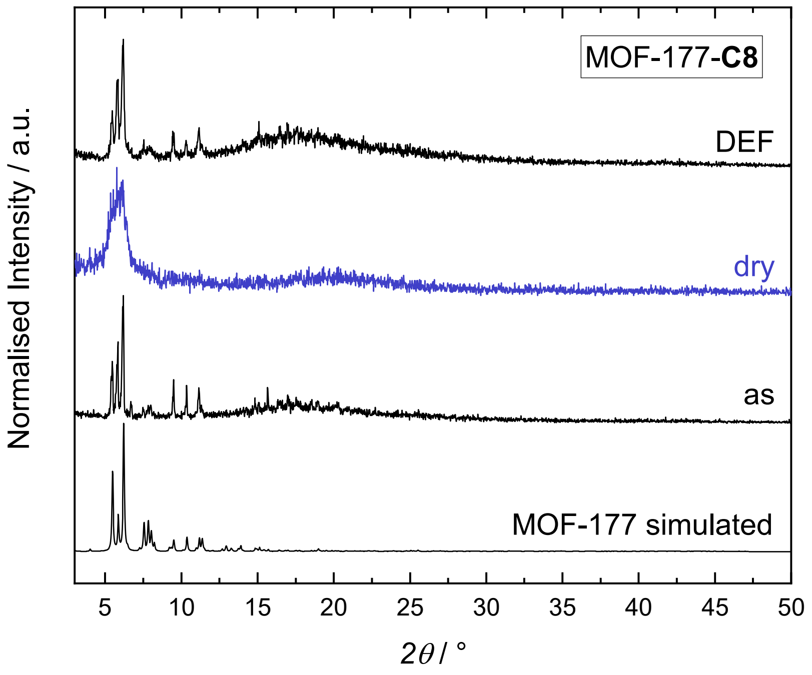

Supplementary Figure 2.15: PXRD patterns of as-MOF-177-C8, dry-MOF177-C8 and DEF-MOF-177-C8 in comparison with a simulated XRPD pattern of MOF-177.[14]

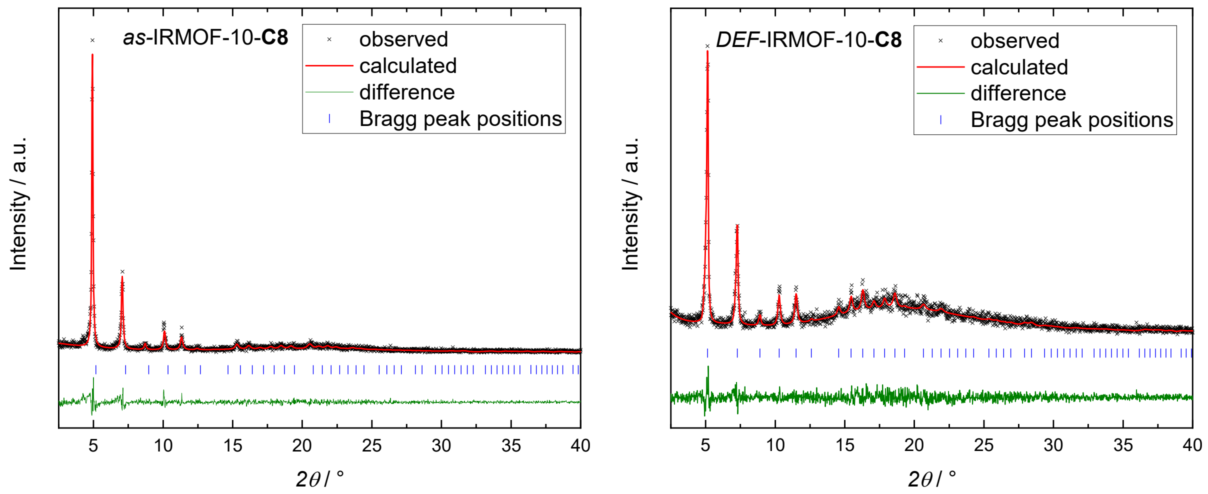

Supplementary Figure 2.16: PXRD pattern with profile fits (Pawley method) of as-IRMOF-10-C8 and DEF-IRMOF-10-C8.

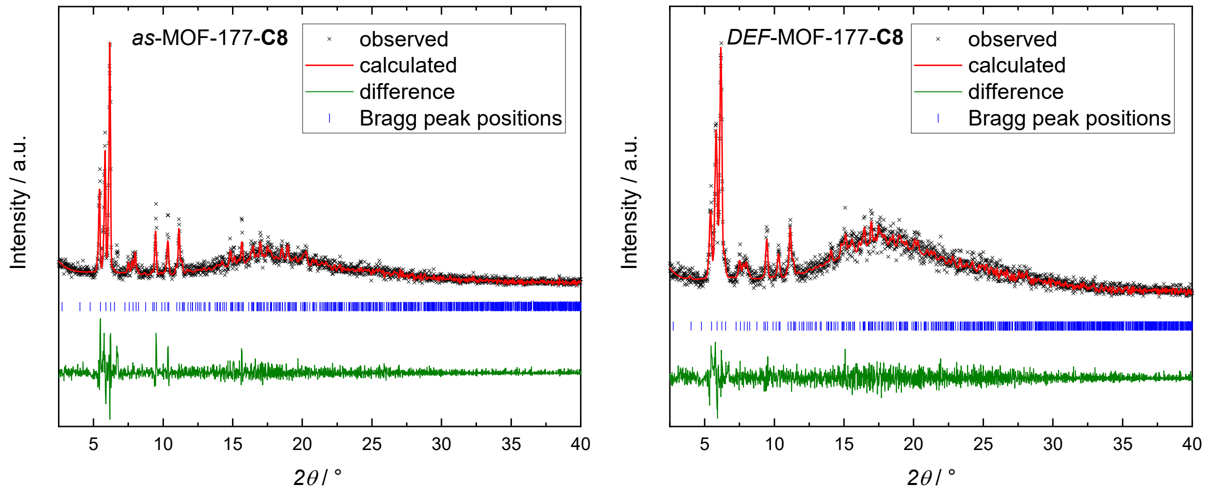

Supplementary Figure 2.17: PXRD pattern with profile fits (Pawley method) of as-MOF-177-C8 and DEF-MOF-177-C8.

Supplementary Table 2.5: Unit cell parameters *a*, *b*, *c* and cell volume *V* determined by Pawley refinement of the PXRD patterns and the corresponding R_{wp}, R_{exp} and χ values for the IRMOF-10-C8 and MOF-177-C8 derivatives. The PXRD data of as- and DEF-MOF-177-C8 were refined using unit cell parameters that were taken from the literature.[8]

Compound	as-*IR*MOF-10-C8	DEF-IRMOF-10-C8	as-MOF-177-C8	DEF-MOF-177-C8
crystal system	cubic	cubic	trigonal	trigonal
space group	$Pm\bar{3}m$	$Pm\bar{3}m$	$P\bar{3}1c$	$P\bar{3}1c$
a / Å	17.083(19)	17.198(9)	37.17(4	37.16(4)
b / Å	17.083(19)	17.198(9)	37.17(4)	37.16(4)
c / Å	17.083(19)	17.198(9)	30.00(4)	30.00(4)
α / °	90	90	90	90
β / °	90	90	90	90
γ / °	90	90	120	120
V / Å^3	4985(17)	5087(8)	35912(86)	35892(99)
R_{wp}	28.77	21.30	21.56	16.99
R_{exp}	23.425	19.36	6.29	5.33
χ^2	1.51	1.21	11.76	0.18

Supplementary Methods 3 - Single crystal X-ray diffraction (SCXRD)

Supplementary Methods 3.1 - Single crystal structure determination

All cubic as-synthesized MOF-5-CX (X = 2-7) crystallize in the cubic space group $Pm\bar{3}m$ as a result of disorder of both the zinc-oxo-clusters and the phenyl ring of the bdc units. Therefore, the Zn and carboxylate O atoms are equally disordered by symmetry over two sites as well as the phenyl rings. Additionally, all ether O atoms are equally disordered over 4 sites. Solvent molecules or the alkyl chains could not be located due to severe disorder, consequently, if present, the corresponding electron density within the pores was subtracted from the experimental data by use of solvent masking as implemented in Olex2[1] (the number of masked electrons per unit cell are given in Supplementary Tables 3.1 to 3.3).

The crystal structure of as-MOF-5-CX (X = 2 – 7) in the space group $Pm\bar{3}m$ feature a unit cell with lattice parameter $a \approx 12.8$ Å. Hence, the unit cell of these structures is about 8-times smaller than the unit cell of conventional MOF-5 ($a \approx 25.7$ Å, space group symmetry $Fm\bar{3}m$). The resulting two-fold static disorder of the Zn_4O clusters and the organic linkers about the 4-fold rotation axis oriented along the $\langle 100 \rangle$ direction, has previously been observed for several other MOF-5 derivatives[5], including IRMOF-5[6], which is identical to as-MOF-5-C5. The static disorder potentially allows for the presence of a twisted configuration of the carboxylate groups of the bdc-type linkers, which generates a translational symmetry instead of inversion symmetry between neighboring $[Zn_4O]^{6+}$ clusters. Nevertheless, previous theoretical calculations indicated that the twisted bdc-confirmation of MOF-5 is significantly higher in energy, even for symmetrically substituted bdc-type linkers[15]. This finding is corroborated by the fact that the as-MOF-5-CX crystal structures (with X = 2 – 7) show very weak and diffuse superstructure reflections on the {hk½} planes. These reflections can be indexed in the larger $Fm\bar{3}m$ unit cell (with $a \approx 25.7$ Å), thus strongly suggesting that the structures of as-MOF-5-CX *locally* feature the same inversion symmetry between neighboring $[Zn_4O]^{6+}$ clusters and no twisted configuration. Note that an all-twisted bdc-configuration exhibiting only translational symmetry between the $[Zn_4O]^{6+}$ clusters would crystallize in a small primitive unit cell (with $a \approx 12.8$ Å) in the polar $P4\bar{3}m$ space group. Thus, the diffuse superstructure reflections are a clear indication of a locally higher symmetry commensurate with a non-twisted structure. Nevertheless, inclusion of the weak and diffuse superstructure reflections in the structure refinement by applying the large unit cell with $Fm\bar{3}m$ symmetry yields the same disordered structural model as in the smaller $Pm\bar{3}m$ cell. Thus, the simpler models of $Pm\bar{3}m$ symmetry are more appropriate to describe the global crystal structures of as-MOF-5-CX (X = 2 –7) and are reported here.

Rhombohedral as-MOF-5-C8 crystallizes in space group $R\bar{3}$ displaying a likewise disordered inorganic building unit. The phenyl ring of the linker is not disordered. The alkyl chains could be resolved and were refined applying a number of bond distance and angle restraints. All carbon atoms of the alkyl chain, except for the ones named C8 and C9, are disordered over two sites and their occupancies were fixed to 0.5 during the refinement. Solvent molecules could not be resolved, and their corresponding electron density was likewise subtracted by masking.

oct-MOF-5-C3 was found to crystallize in the face centered cubic space group $Fm\bar{3}m$ as a consequence of the disorder of the Zn and carboxylate O atoms over two sites with fractional

occupancies of 0.73/0.27 (instead of the fractional occupancies of 0.5/0.5 found for the other materials crystallizing in the smaller $Pm\overline{3}m$ cell), similar as initially found by M. Eddaoudi *et al.*[6] The fractional occupancies of 0.73/0.27 are also a strong indication that neighboring $[Zn_4O]^{6+}$ clusters are related by inversion symmetry on a local scale. The phenyl ring carbon atoms and ether O atoms of the linker are disordered by symmetry over two and four sites, respectively. Solvent molecules or alkyl chains could not be located due to severe disorder and were therefore subtracted from the experimental data by use of the solvent masking routines of Olex2.

oct-MOF-5-C6 was found to crystallize in the primitive cubic space group $Pa\overline{3}$. The crystal used for data collection was twinned, therefore the experimental data were refined applying the merohedral twin law (-1 0 0 0 0 1 0 1 0) and the twinning factor of 0.482(3), which were determined using the TwinRotMat implementation of the PLATON[16] software. Structurally, the Zn and carboxylate O atoms of the inorganic units are disordered over two sites, similar to all the other structures reported above. Free refinement of the occupancies of these sites gave values very close to 0.5, consequently the occupancies were set to fix values of 0.5. Interestingly, and similar to the rhombohedral structure of as-MOF-5-C8, the phenyl rings of the bdc type linker is not disordered here. The alkyl chains could be resolved (with disorder over two positions, except for carbon atoms C9, C15 and C16) and were refined applying a number of bond distance and angle restraints. Occupancies of the disordered carbon atoms of the alkyl chains could not be refined freely and were set to fixed values of 0.5/0.5 or 0.35/0.65. Solvent molecules could not be located. Further details to all of the SCXRD structures can be found in the provided crystallographic information files (CCDC deposition numbers 2040916 to 2040922, 2040924 and 2040925).

Supplementary Table 3.1: Crystallographic data of as-synthesized MOF-5-C2 to MOF-5-C4.

Compound	as-MOF-5-C2	as-MOF-5-C3	as-MOF-5-C4
Empirical formula	$C_{36}H_{36}O_{19}Zn_4$	$C_{42}H_{48}O_{19}Zn_4$	$C_{48}H_{60}O_{19}Zn_4$
Formula weight / g·mol^{-1}	1034.13	1118.28	1202.44
Temperature / K	250.0(2)	254(2)	293(2)
Crystal system	cubic	cubic	cubic
Space group	$Pm\bar{3}m$	$Pm\bar{3}m$	$Pm\bar{3}m$
a / Å	12.813(2)	12.8791(4)	12.8018(12)
b / Å	12.813(2)	12.8791(4)	12.8018(12)
c / Å	12.813(2)	12.8791(4)	12.8018(12)
α / °	90	90	90
β / °	90	90	90
γ / °	90	90	90
Volume / Å^3	2103.7(10)	2136.3(2)	2098.0(6)
Z	1	1	1
ρ_{calc} g/cm^3	0.816	0.876	0.952
μ / mm^{-1}	1.164	1.159	1.175
F(000)	524.0	577.0	620.0
Crystal size / mm^3	0.24 × 0.236 × 0.222	0.437 × 0.427 × 0.396	0.479 × 0.444 × 0.284
Radiation	MoKα (λ = 0.71073)	MoKα (λ = 0.71073)	MoKα (λ = 0.71073)
2Θ range for data collection / °	5.506 to 54.98	4.472 to 54.994	5.512 to 54.924
Index ranges	-16 ≤ h ≤ 16, -16 ≤ k ≤ 16, -16 ≤ l ≤ 16	-16 ≤ h ≤ 16, -16 ≤ k ≤ 16, -16 ≤ l ≤ 16	-4 ≤ h ≤ 16, -16 ≤ k ≤ 16, -11 ≤ l ≤ 16
Reflections collected	25791	50399	4002
Independent reflections	544 [R_{int}= 0.1614, R_{sigma}= 0.0726]	548 [R_{int}= 0.0714, R_{sigma}= 0.0142]	541 [R_{int}= 0.0772, R_{sigma}= 0.0432]
Data/restraints/parameters	544/13/29	548/13/32	541/20/26
Goodness-of-fit on F^2	1.209	1.220	1.176
Final R indexes [I ≥ 2σ(I)]	R_1= 0.0974, wR_2= 0.2799	R_1= 0.0947, wR_2= 0.2836	R_1= 0.1200, wR_2= 0.3425
Final R indexes [all data]	R_1= 0.1275, wR_2= 0.3612	R_1= 0.1119, wR_2= 0.3160	R_1= 0.1511, wR_2= 0.3885
Largest diff. peak/hole / e·Å^{-3}	0.46/-0.92	0.53/-0.58	0.47/-0.60
Number of masked electrons	246.0	0.0	108.6
CCDC deposition number	2040917	2040918	2040919

Supplementary Table 3.2: Crystallographic data of as-synthesized MOF-5-C5 to MOF-5-C7.

Compound	as-MOF-5-C5	as-MOF-5-C6	as-MOF-5-C7
Empirical formula	$C_{54}H_{72}O_{19}Zn_4$	$C_{60}H_{84}O_{19}Zn_4$	$C_{66}H_{96}O_{19}Zn_4$
Formula weight / g·mol^{-1}	1286.59	1370.75	1454.90
Temperature / K	284.78(2)	250.0(2)	110.02(2)
Crystal system	cubic	cubic	cubic
Space group	$Pm\bar{3}m$	$Pm\bar{3}m$	$Pm\bar{3}m$
a / Å	12.8898(15)	12.9040(12)	12.8526(5)
b / Å	12.8898(15)	12.9040(12)	12.8526(5)
c / Å	12.8898(15)	12.9040(12)	12.8526(5)
α / °	90	90	90
β / °	90	90	90
γ / °	90	90	90
Volume / Å^3	2141.6(7)	2148.7(6)	2123.1(2)
Z	1	1	1
ρ_{calc} g/cm^3	0.998	1.068	1.138
μ / mm^{-1}	1.154	1.163	1.740
F(000)	668.0	722.0	764.0
Crystal size / mm^3	0.23 × 0.18 × 0.124	0.41 × 0.336 × 0.29	0.223 × 0.16 × 0.119
Radiation	MoKα (λ = 0.71073)	MoKα (λ = 0.71073)	CuKα (λ = 1.54178)
2Θ range for data collection / °	4.468 to 54.09	5.468 to 57.392	9.732 to 148.906
Index ranges	-16 ≤ h ≤ 15, -15 ≤ k ≤ 16, -16 ≤ l ≤ 11	-17 ≤ h ≤ 16, -16 ≤ k ≤ 17, -17 ≤ l ≤ 9	-13 ≤ h ≤ 12, -15 ≤ k ≤ 15, -16 ≤ l ≤ 10
Reflections collected	7925	8092	10658
Independent reflections	532 [R_{int}= 0.0462, R_{sigma}= 0.0204]	611 [R_{int}= 0.0565, R_{sigma}= 0.0249]	490 [R_{int}= 0.0264, R_{sigma}= 0.0112]
Data/restraints/parameters	532/13/29	611/1/32	490/7/32
Goodness-of-fit on F^2	1.584	1.119	1.137
Final R indexes [I ≥ 2σ(I)]	R_1= 0.0972, wR_2= 0.3011	R_1= 0.0819, wR_2= 0.2352	R_1= 0.0616, wR_2= 0.1780
Final R indexes [all data]	R_1= 0.1258, wR_2= 0.3729	R_1= 0.0982, wR_2= 0.2717	R_1= 0.0632, wR_2= 0.1835
Largest diff. peak/hole / e·Å^{-3}	0.57/-0.78	0.34/-0.72	0.29/-0.35
Number of masked electrons	0.0	328.0	84.8
CCDC deposition number	2040920	2040921	2040922

Supplementary Table 3.3: Crystallographic data of as-synthesized MOF-5-C8, *n*-octane resolvated MOF-5-C3 and n-octane resolvated MOF-5-C6.

Compound	as-MOF-5-C8	oct-MOF-5-C3	oct-MOF-5-C6
Empirical formula	$C_{72}H_{108}O_{19}Zn_4$	$C_{42}H_{48}O_{19}Zn_4$	$C_{60}H_{84}O_{19}Zn_4$
Formula weight / g·mol^{-1}	1539.06	1118.28	1370.75
Temperature / K	293(2)	250(2)	250(2)
Crystal system	trigonal	cubic	cubic
Space group	$R\bar{3}$	$Fm\bar{3}m$	$Pa\bar{3}$
a / Å	17.7158(10)	25.7952(9)	25.7145(6)
b / Å	17.7158(10)	25.7952(9)	25.7145(6)
c / Å	23.4128(11)	25.7952(9)	25.7145(6)
α / °	90	90	90
β / °	90	90	90
γ / °	120	90	90
Volume / Å^3	6363.6(8)	17163.9(18)	17003.3(12)
Z	3	8	8
ρ_{calc} g/cm^3	1.606	0.866	1.071
μ / mm^{-1}	1.568	1.145	1.712
F(000)	3248.0	4576.0	5728.0
Crystal size / mm^3	0.208 × 0.196 × 0.162	0.208 × 0.205 × 0.133	0.187 × 0.145 × 0.076
Radiation	MoKα (λ = 0.71073)	MoKα (λ = 0.71073)	CuKα (λ = 1.54178)
2Θ range for data collection / °	4.376 to 64.906	4.466 to 60.968	3.436 to 144.808
Index ranges	-26 ≤ h ≤ 25, -26 ≤ k ≤ 25, -35 ≤ l ≤ 35	-36 ≤ h ≤ 23, -35 ≤ k ≤ 35, -34 ≤ l ≤ 33	-21 ≤ h ≤ 31, -29 ≤ k ≤ 31, -31 ≤ l ≤ 23
Reflections collected	17446	19056	90399
Independent reflections	4735 [R_{int}= 0.0578, R_{sigma}= 0.0615]	1356 [R_{int}= 0.0588, R_{sigma}= 0.0234]	5804 [R_{int} = 0.1027, R_{sigma} = 0.0390]
Data/restraints/parameters	4735/53/149	1356/6/46	5804/60/275
Goodness-of-fit on F^2	1.031	1.129	1.123
Final R indexes [I ≥ 2σ(I)]	R_1= 0.0923, wR_2= 0.2707	R_1= 0.0777, wR_2= 0.2353	R_1 = 0.1031, wR_2 = 0.2738
Final R indexes [all data]	R_1= 0.0923, wR_2= 0.2707	R_1= 0.1017, wR_2= 0.2860	R_1 = 0.1160, wR_2 = 0.2873
Largest diff. peak/hole / e·Å^{-3}	0.79/-0.45	0.59/-1.15	0.48/-0.41
Number of masked electrons	36.1	0.0	-
CCDC deposition number	2040916	2040924	2040925

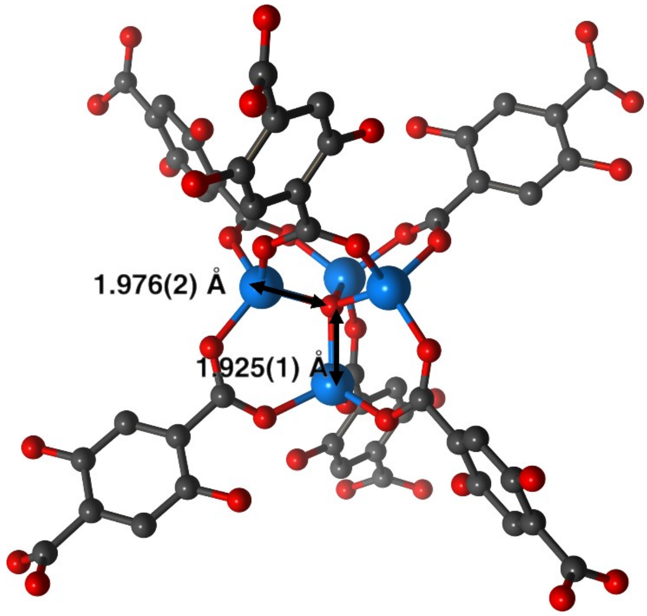

Supplementary Figure 3.1: Representation of the SCXRD structure of as-MOF-5-C8 (alkyl-groups and hydrogen atoms are omitted for clarity) showing the rhombohedrally distorted Zn_4O node with marked Zn-O bond lengths. The Zn atoms and carboxylate groups are statically disordered by symmetry. The second part has been removed for clarity. Note that the corresponding Zn-O bond lengths in MOF-5 are given as 1.935(2) Å (CCDC 256965)[17].

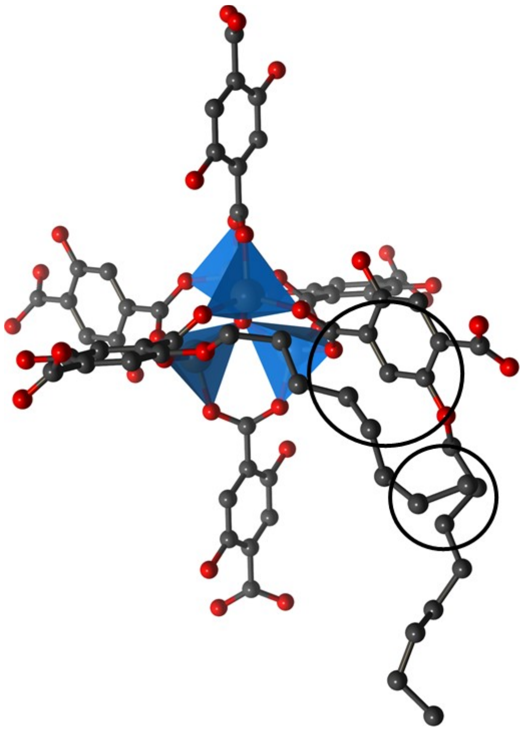

Supplementary Figure 3.2: Representation of the SCXRD structure of as-MOF-5-C8 (hydrogen atoms are omitted for clarity) showing the close contact between the alky-chains and the aromatic ring of the framework backbone (circles). Disorder has been removed for clarity.

Supplementary Methods 3.3 - Precession images

All displayed precession images were reconstructed from processed experimental diffraction data using the APEX3 software. Diffraction images of dry-MOF-5-C3 were collected using CuKα (λ = 1.54178) radiation. Precession images of dry-MOF-5-C6 were calculated from diffraction images collected at 100 K during a 180° φ scan with an angular step size of 1° and 1 s exposure time using CuKα (λ = 1.54178) radiation.

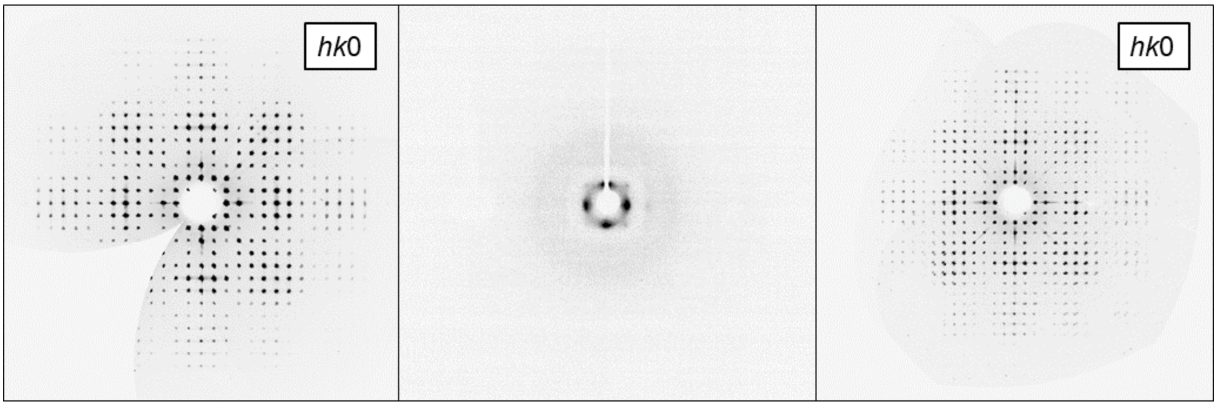

Supplementary Figure 3.3: Reconstructed precession images of as-MOF-5-C3 (left) and oct-MOF-5-C3 (right) showing the *hk*0 layers of reciprocal space. In the middle, a single diffraction pattern of dry-MOF-5-C3 is shown. The four-fold symmetry of the non-crystalline structure is still visible in the diffuse scattering peaks of dry-MOF-5-C3. Note that as-MOF-5-C3 crystallizes in the space group $Pm\bar{3}m$ with *a* = 12.8791 Å while oct-MOF-5-C3 crystallizes in the space group $Fm\bar{3}m$ with *a* = 25.7952 Å.

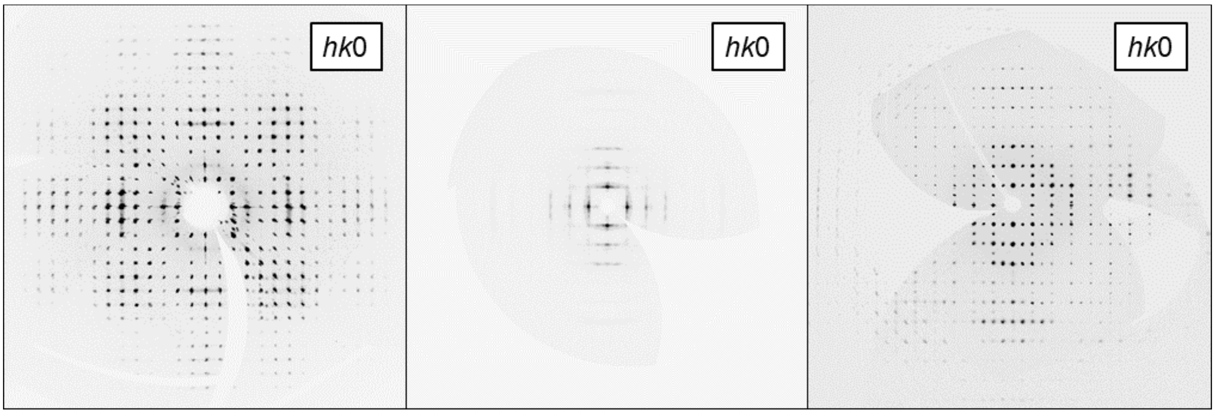

Supplementary Figure 3.4: Reconstructed precession images of as-, dry- and oct-MOF-5-C6 (from left to right) showing the *hk*0 layers of reciprocal space. Note that as-MOF-5-C6 crystallizes in the space group $Pm\bar{3}m$ with *a* = 12.9040 Å while oct-MOF-5-C6 crystallizes in the space group $Pa\bar{3}$ with *a* = 25.7145 Å.

Supplementary Methods 3.4 - Crystal mosaicities

Supplementary Table 3.4: Mosaicities of selected MOF-5-CX single crystals**.**

Compound	Mosaicity / °
as-MOF-5-C3	0.89
oct-MOF-5-C3	0.67
as-MOF-5-C6	0.86
oct-MOF-5-C6	0.98

Supplementary Methods 4 - Solution ^{1}H NMR spectroscopy of digested MOF samples

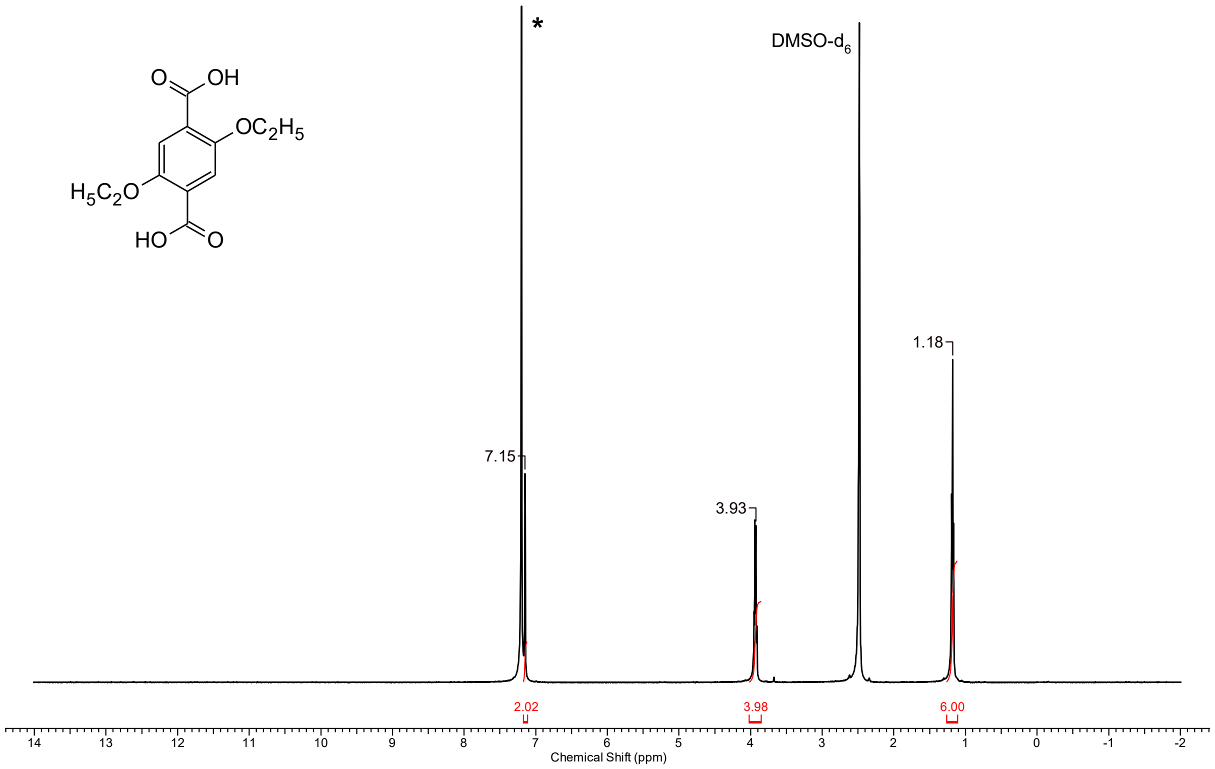

Supplementary Figure 4.1: ^{1}H NMR spectrum of a digested sample of dry-MOF-5-C2. The D_2O signal is marked with an asterisk (*).

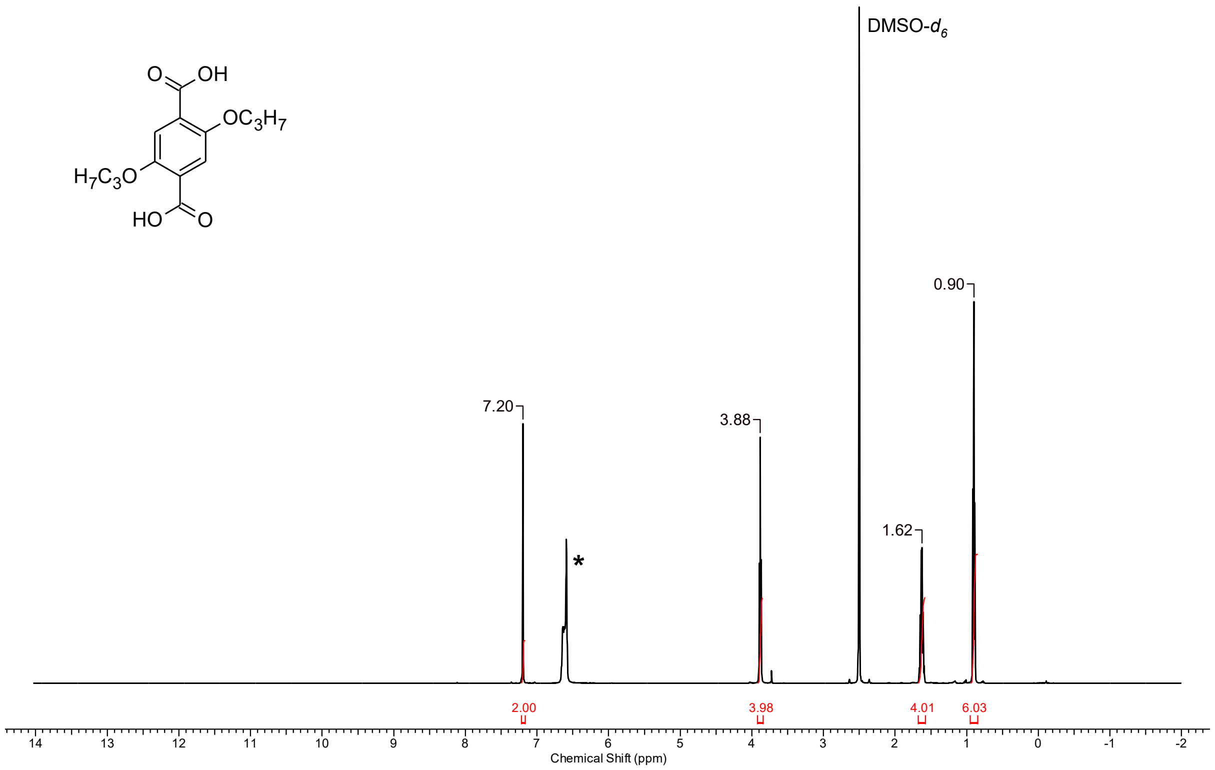

Supplementary Figure 4.2: ^{1}H NMR spectrum of a digested sample of dry-MOF-5-C3. The D_2O signal is marked with an asterisk (*).

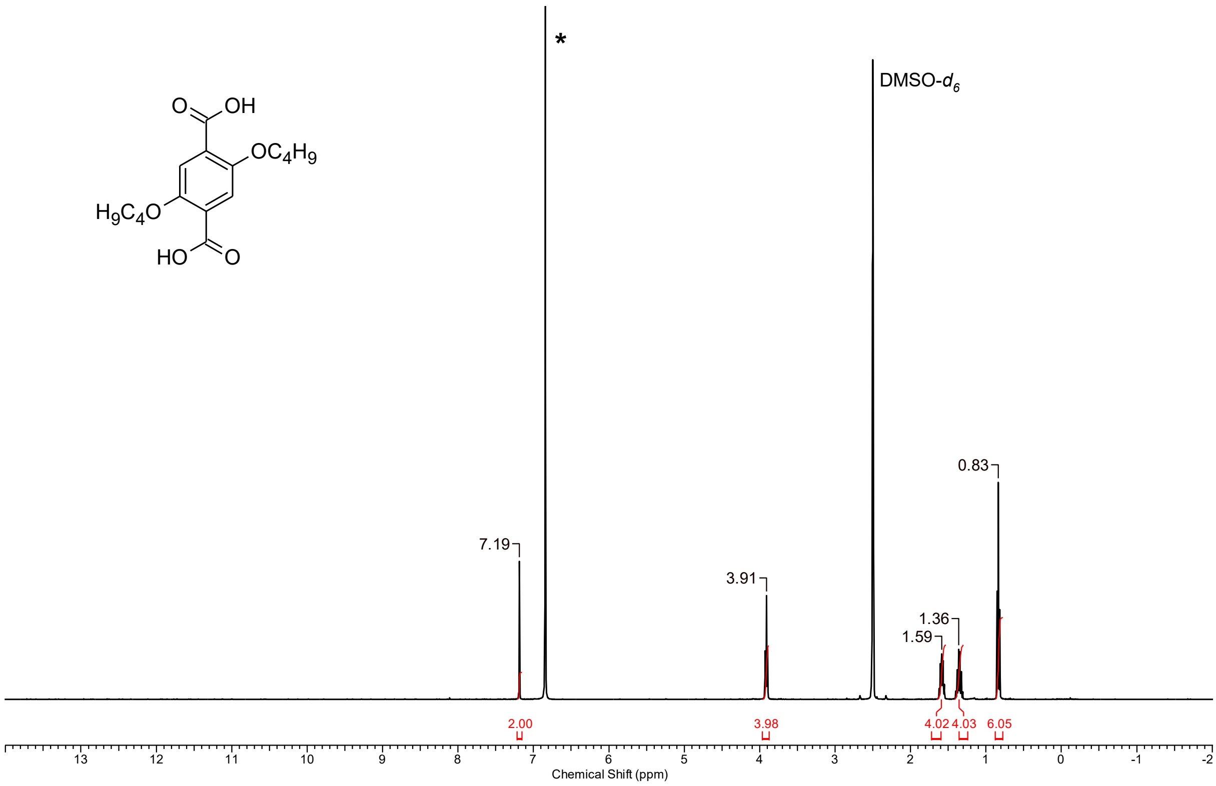

Supplementary Figure 4.3: ^{1}H NMR spectrum of a digested sample of dry-MOF-5-C4. The D_2O signal is marked with an asterisk (*).

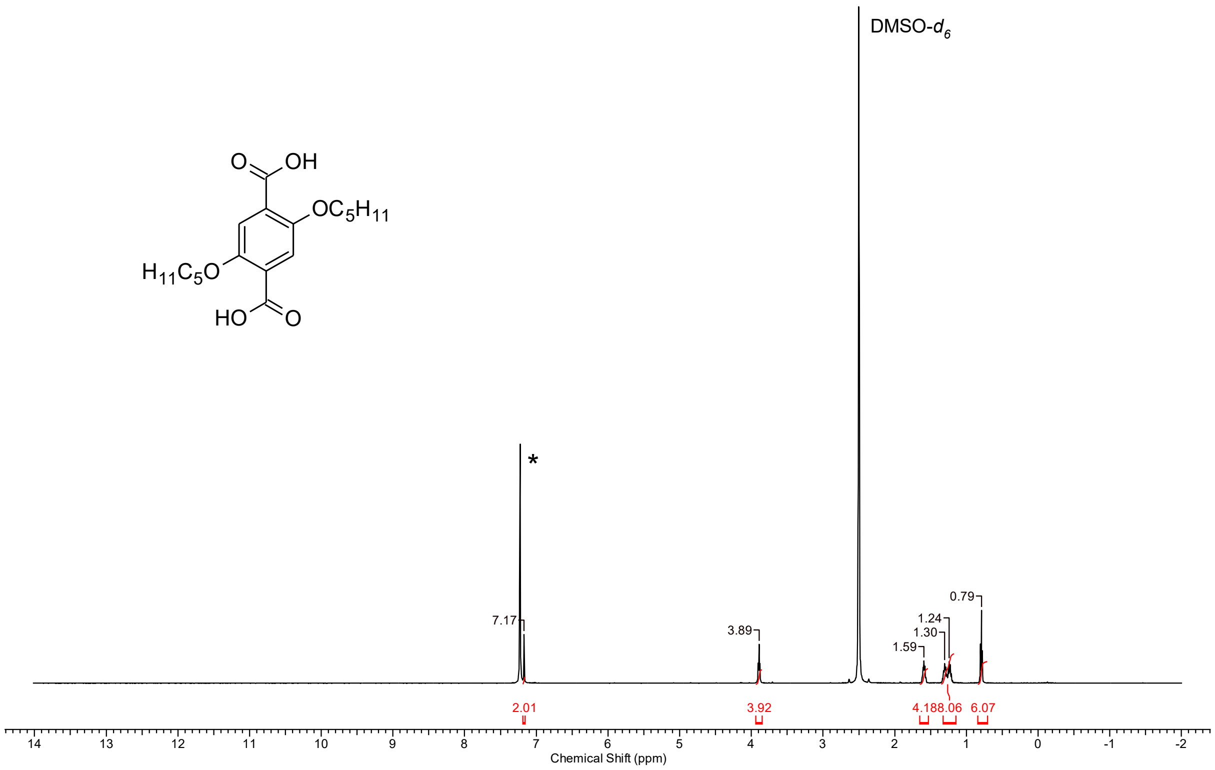

Supplementary Figure 4.4: ^{1}H NMR spectrum of a digested sample of dry-MOF-5-C5. The D_2O signal is marked with an asterisk (*).

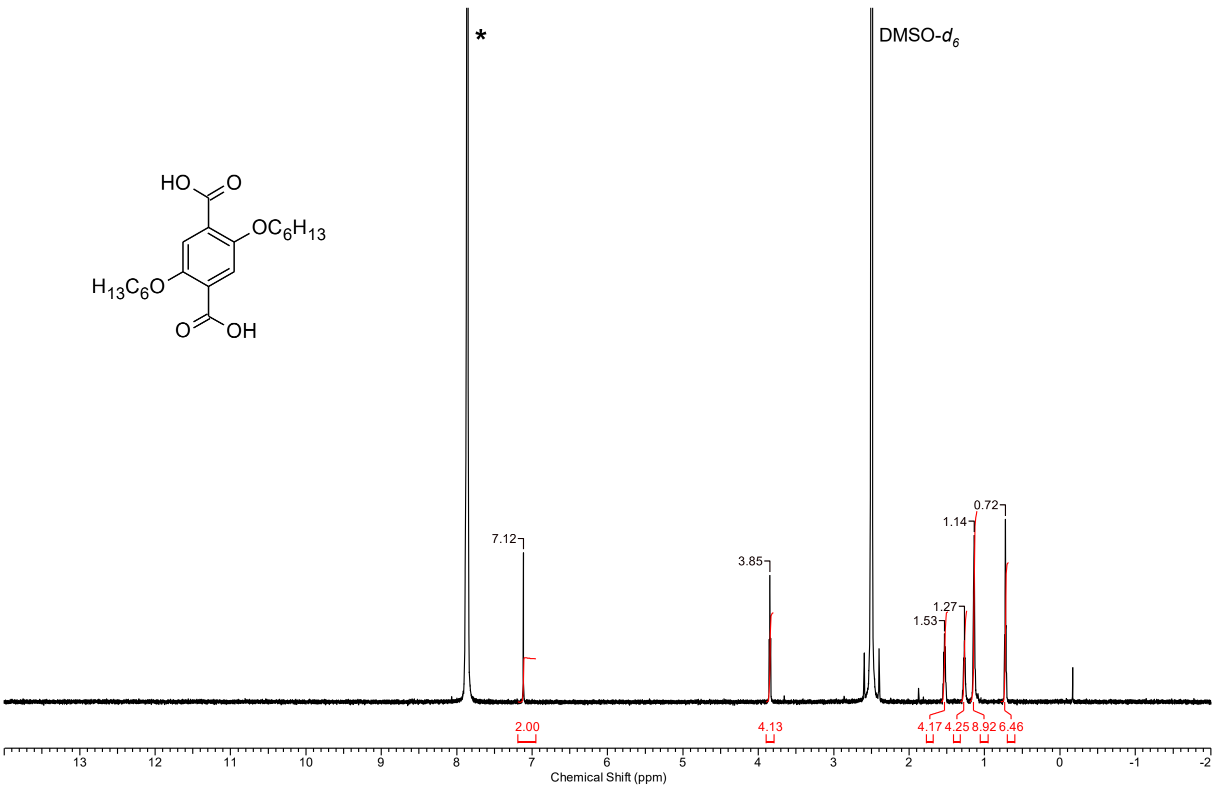

Supplementary Figure 4.5: ^{1}H NMR spectrum of a digested sample of dry-MOF-5-C6. The D_2O signal is marked with an asterisk (*).

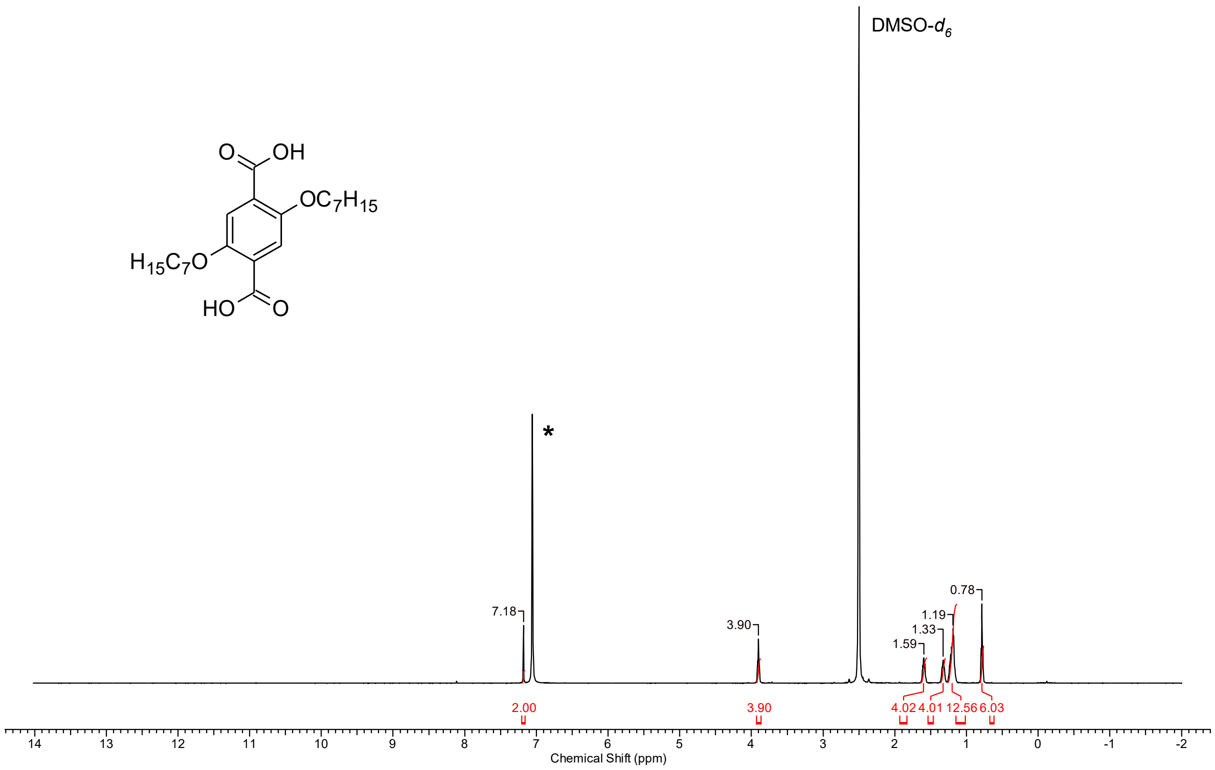

Supplementary Figure 4.6: ^{1}H NMR spectrum of a digested sample of dry-MOF-5-C7. The D_2O signal is marked with an asterisk (*).

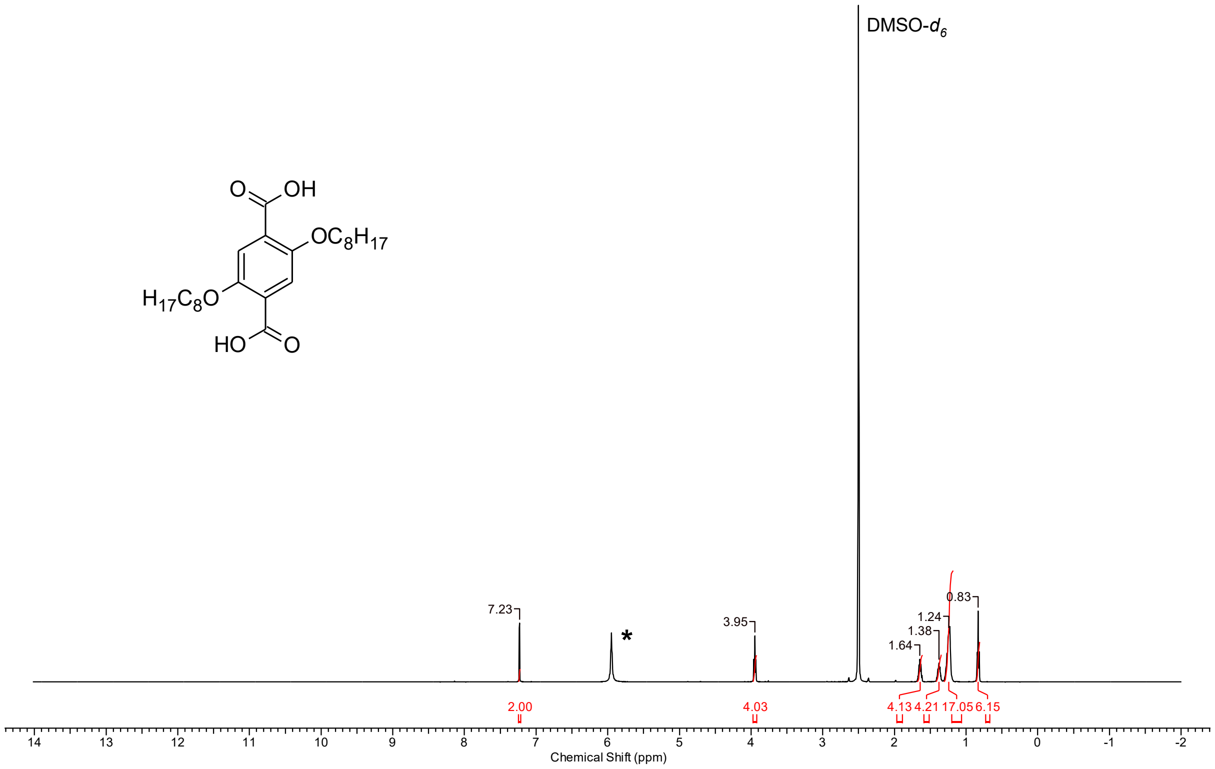

Supplementary Figure 4.7: ^{1}H NMR spectrum of a digested sample of dry-MOF-5-C8. The D_2O signal is marked with an asterisk (*).

Supplementary Methods 5 - X-ray pair distribution function analysis

For the as-synthesized materials, the correct chemical compositions were determined by use ^1H NMR spectroscopy, since the pores of the MOF-5-CX are filled with a number of DMF molecules (see Supplementary Methods 1.4, Supplementary Table 1).

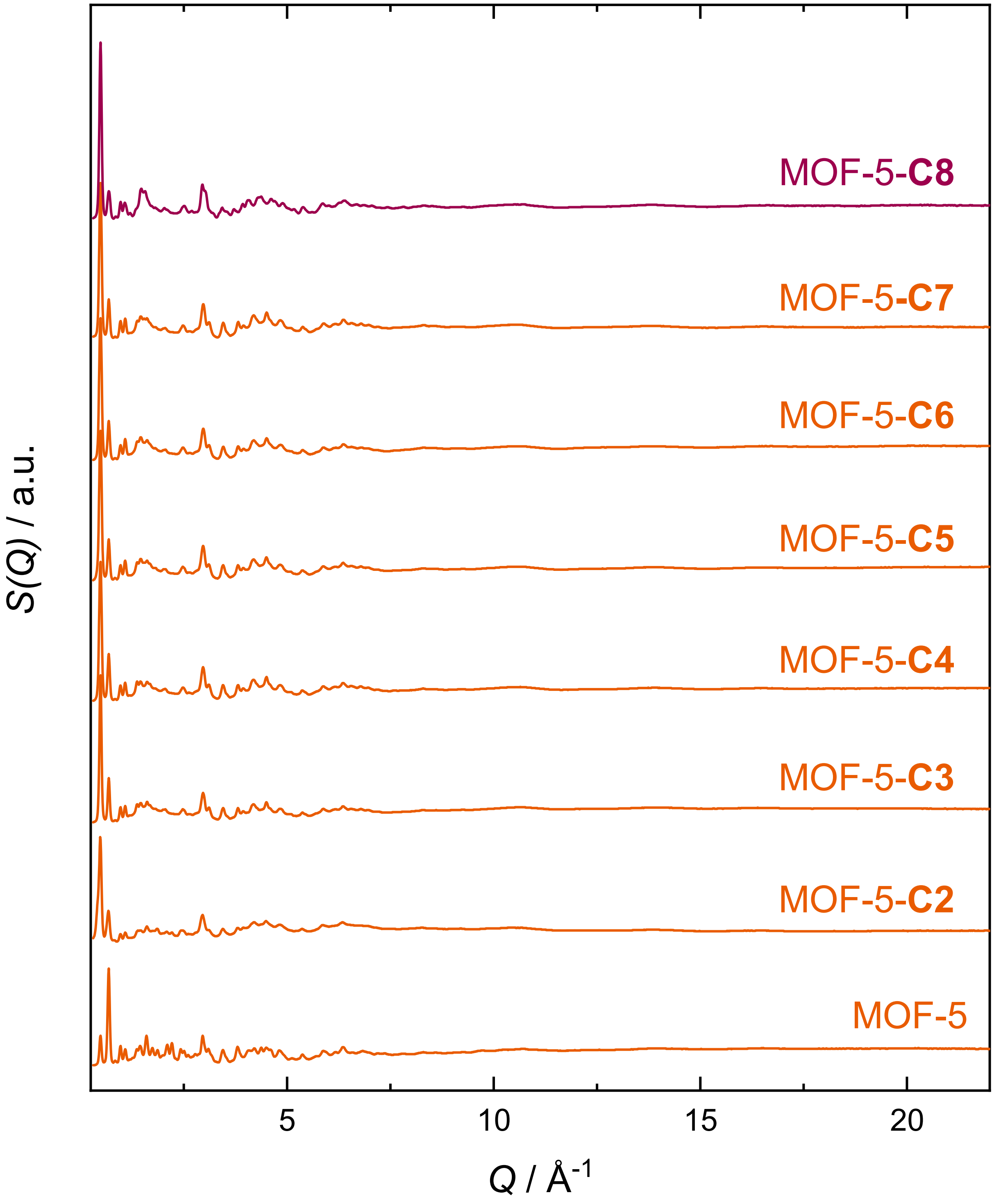

Supplementary Figure 5.1: X-ray scattering factors $S(Q)$ for as-synthesized MOF-5 and the as-MOF-5-CX determined from total scattering experiments at I15-1 of Diamond Light Source.

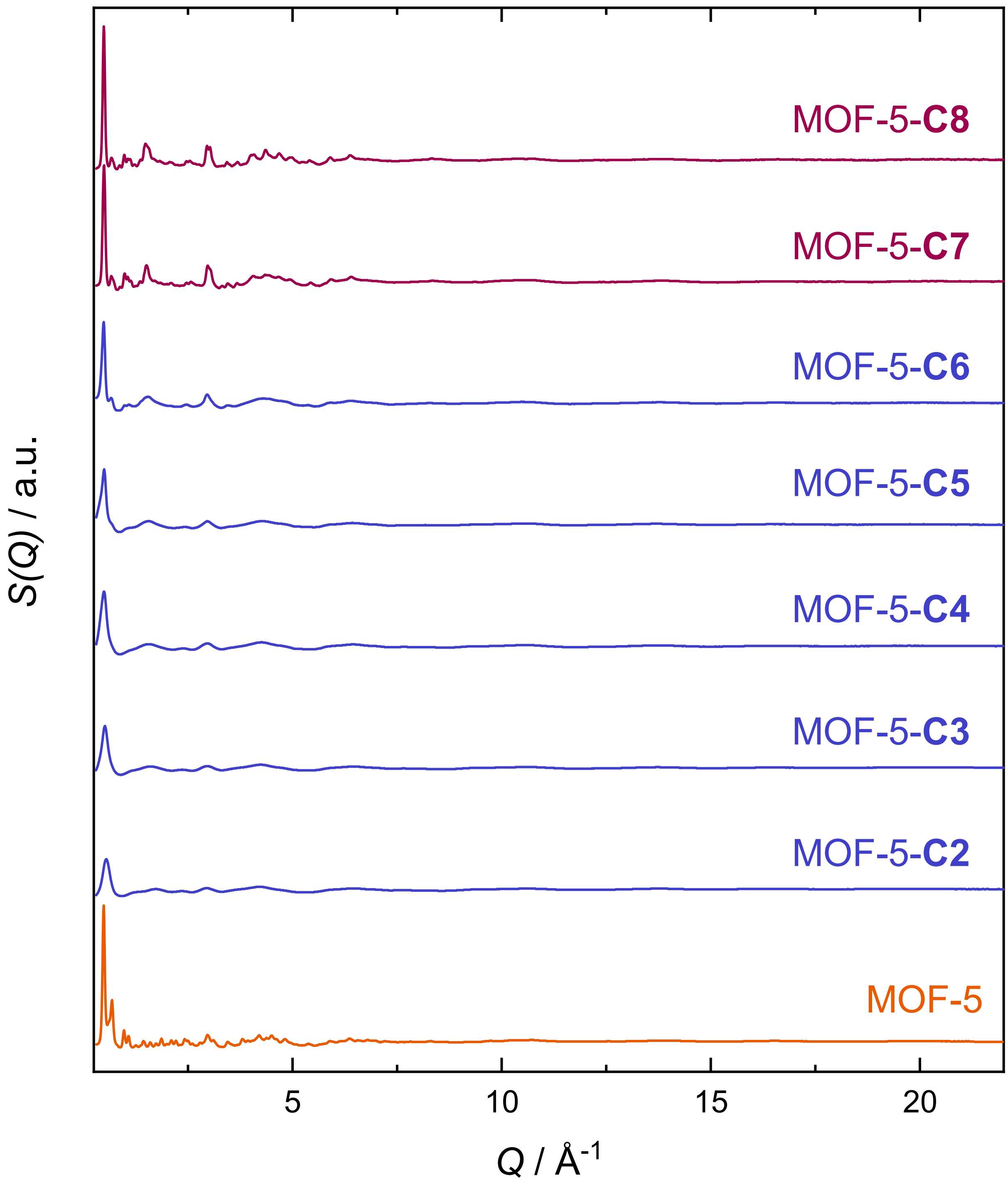

Supplementary Figure 5.2: X-ray scattering factors *S(Q)* for dried MOF-5 and the dry-MOF-5-CX determined from total scattering experiments at I15-1 of Diamond Light Source.

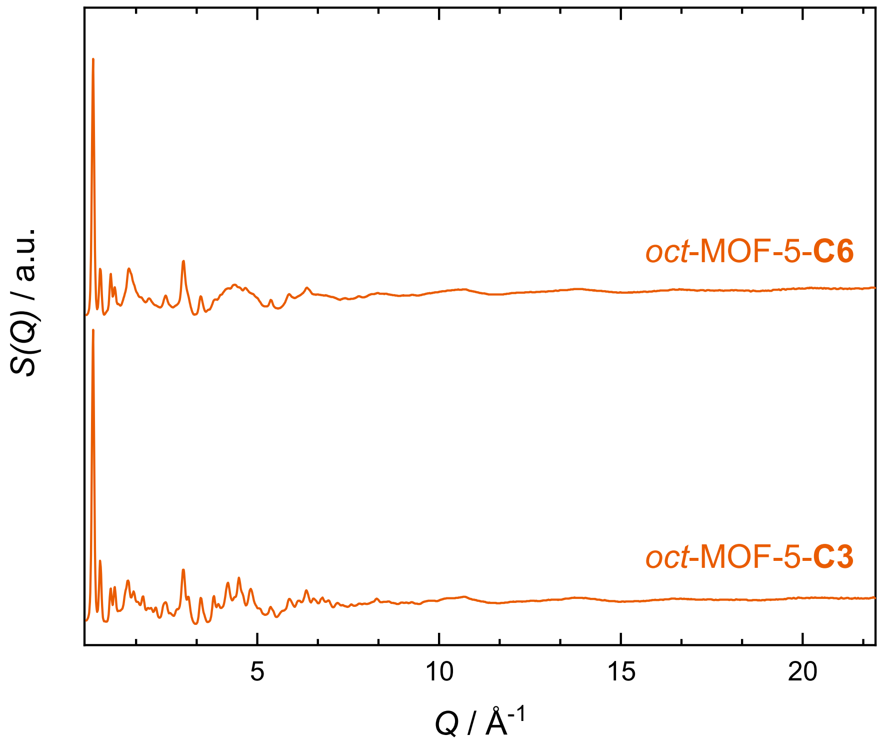

Supplementary Figure 5.3: X-ray scattering factors *S(Q)* for oct-MOF-5-C3 and oct-MOF-5-C6 determined from total scattering experiments at I15-1 of Diamond Light Source.

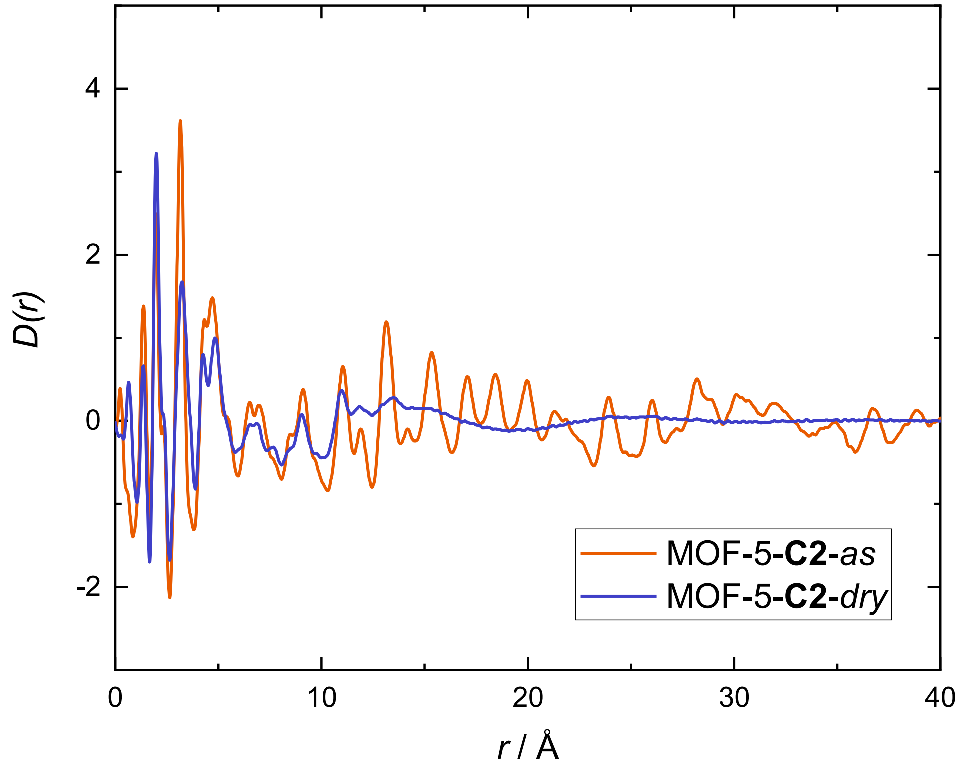

Supplementary Figure 5.4: X-ray pair distribution functions *D(r)* of as- and dry-MOF-5-C2 obtained via Fourier transform of the *S(Q)* shown above.

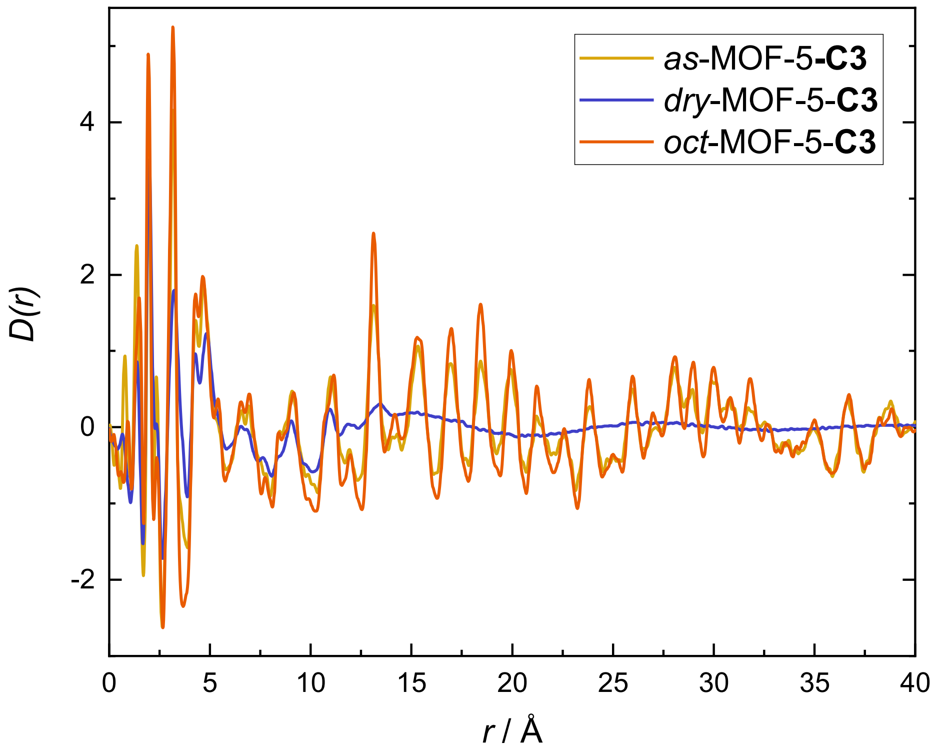

Supplementary Figure 5.5: X-ray pair distribution functions *D(r)* of as-, dry- and oct-MOF-5-C3 obtained via Fourier transform of the *S(Q)* shown above.

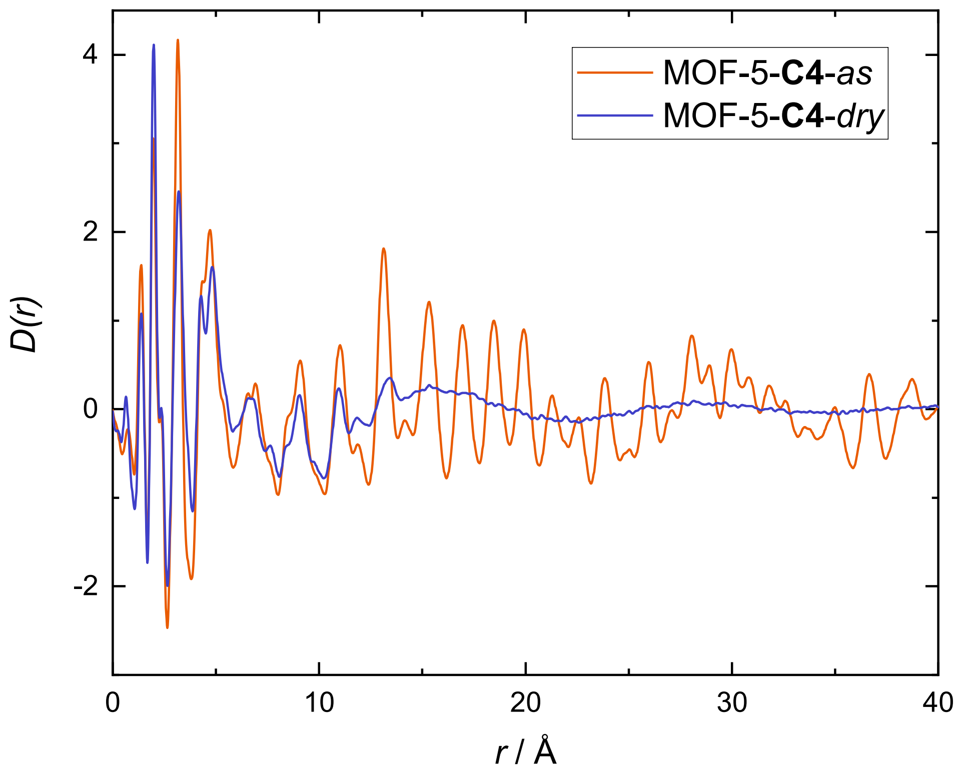

Supplementary Figure 5.6: X-ray pair distribution functions *D(r)* of as- and dry-MOF-5-C4 obtained via Fourier transform of the *S(Q)* shown above.

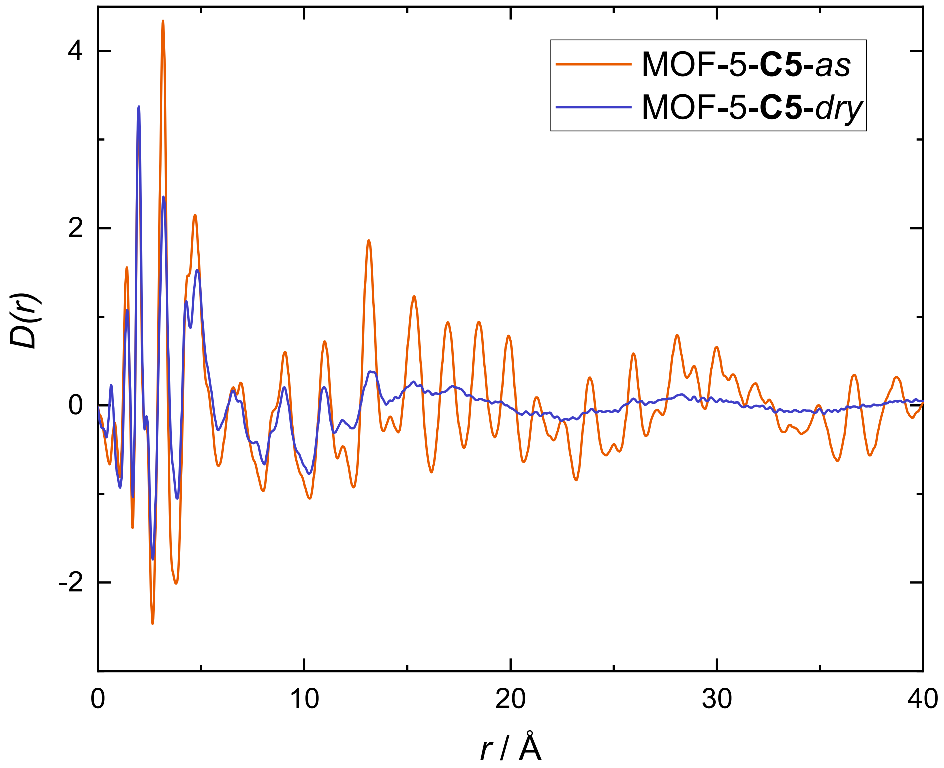

Supplementary Figure 5.7: X-ray pair distribution functions *D(r)* of as- and dry-MOF-5-C5 obtained via Fourier transform of the *S(Q)* shown above.

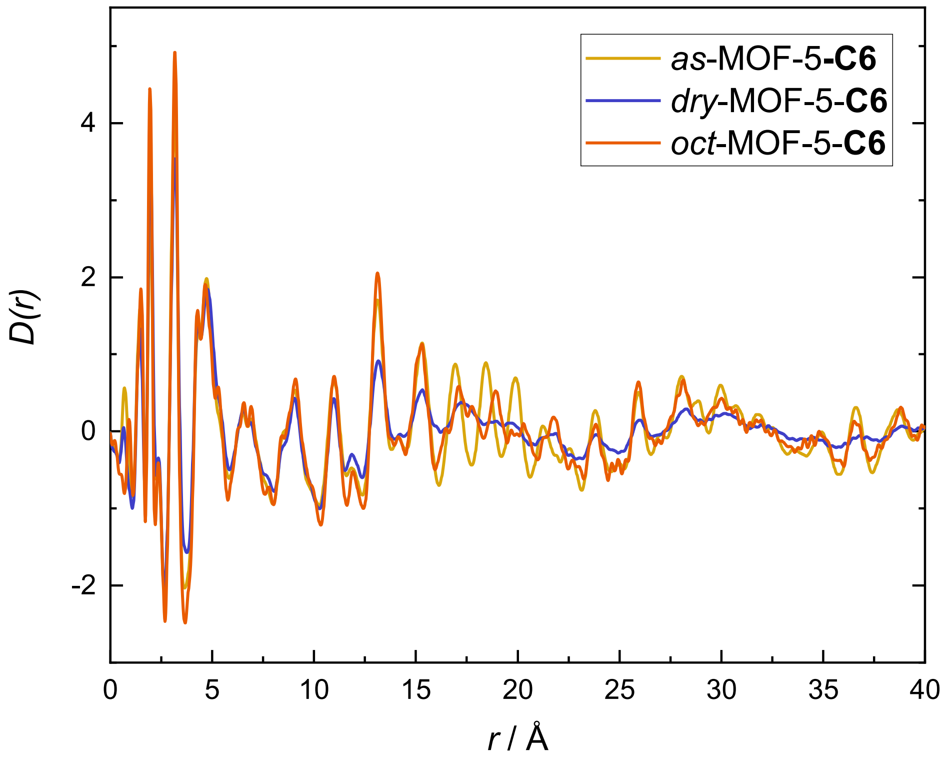

Supplementary Figure 5.8: X-ray pair distribution functions *D(r)* of as-, dry- and oct-MOF-5-C6 obtained via Fourier transform of the *S(Q)* shown above.

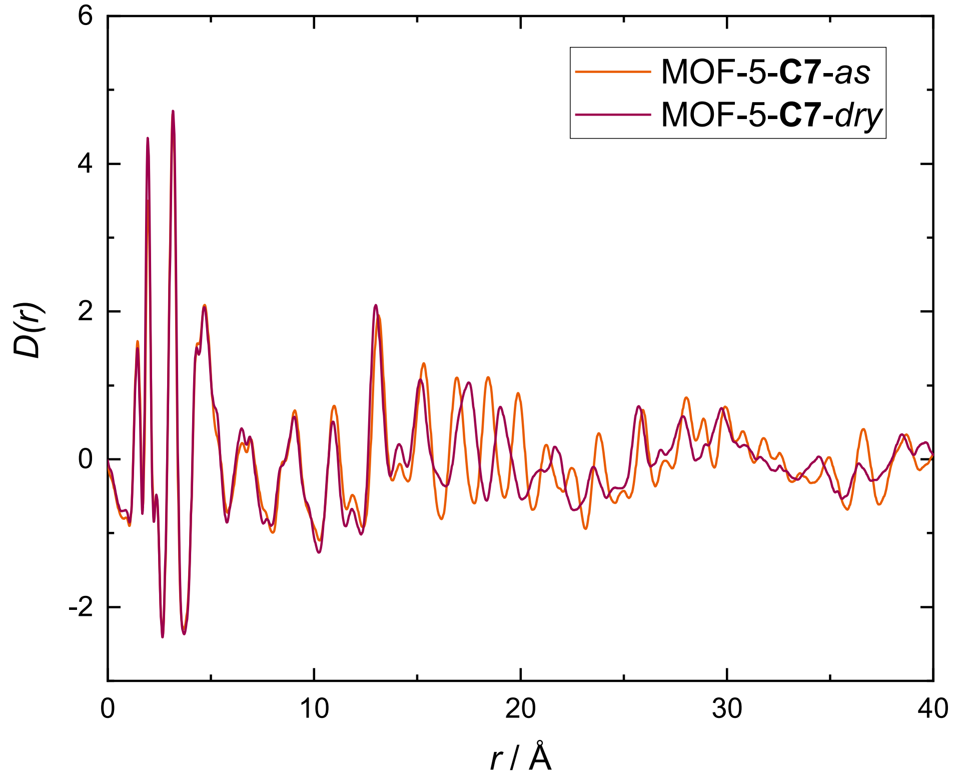

Supplementary Figure 5.9: X-ray pair distribution functions *D(r)* of as- and dry-MOF-5-C7 obtained via Fourier transform of the *S(Q)* shown above.

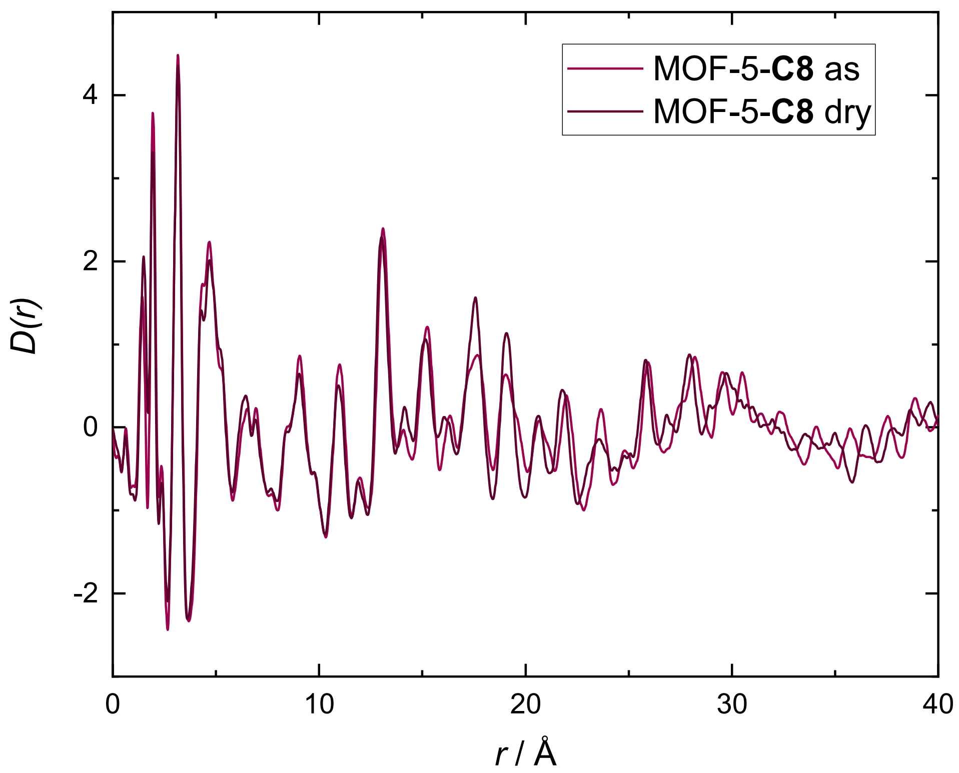

Supplementary Figure 5.10: X-ray pair distribution functions *D(r)* of as- and dry-MOF-5-C8 obtained via Fourier transform of the *S(Q)* shown above.

Supplementary Methods 6 - IR spectroscopy

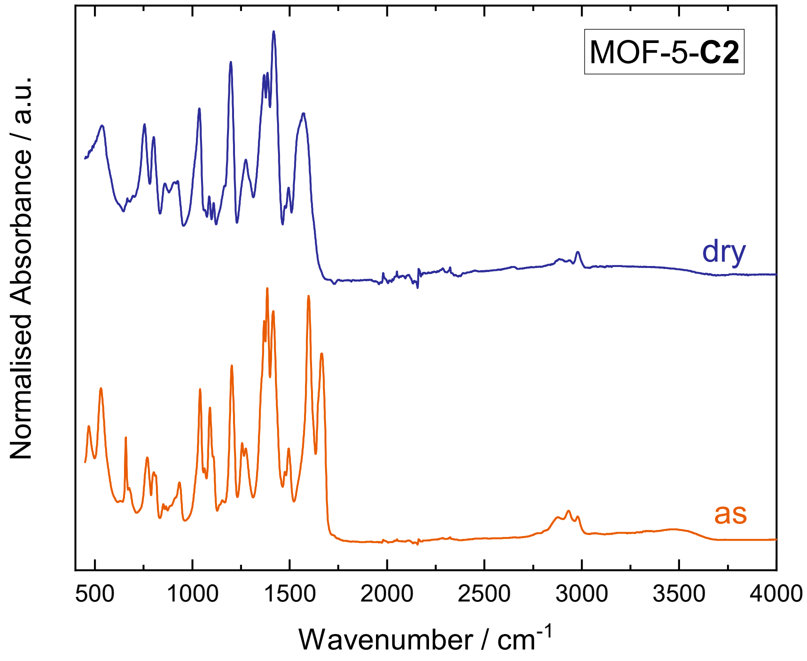

Supplementary Figure 6.1: FT-IR spectra of as-MOF-5-C2 and dry-MOF-5-C2.

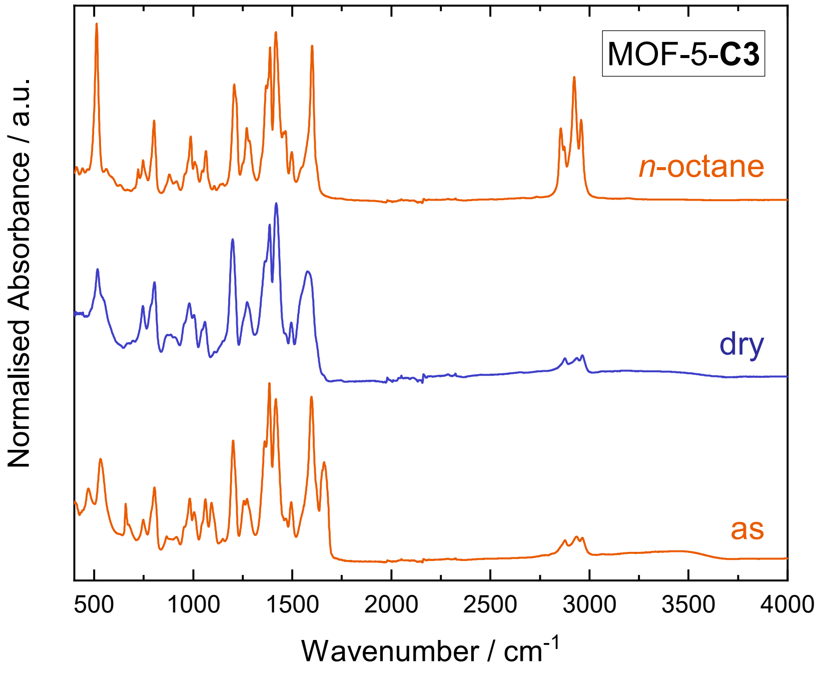

Supplementary Figure 6.2: FT-IR spectra of as-, dry- and n-octane reinfiltrated MOF-5-C3.

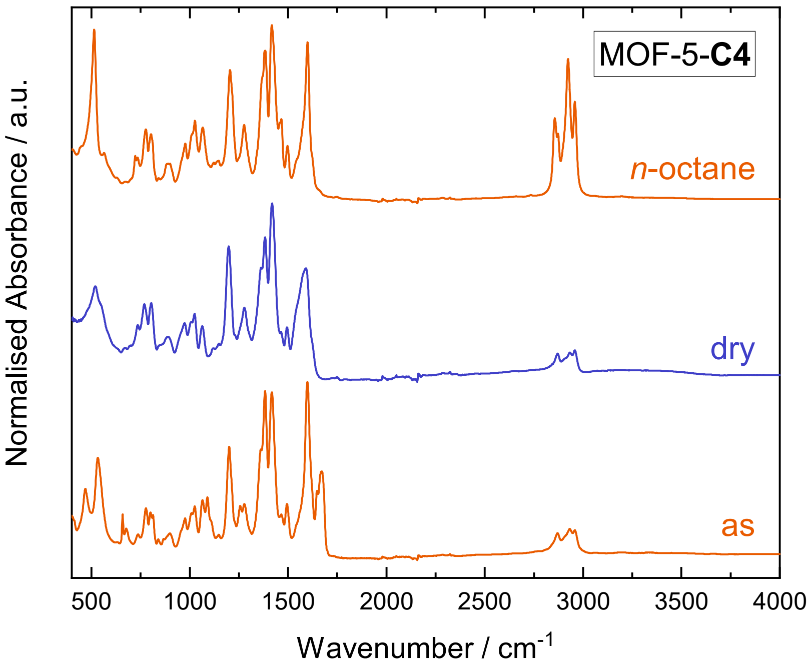

Supplementary Figure 6.3: FT-IR spectra of as-, dry- and n-octane reinfiltrated MOF-5-C4.

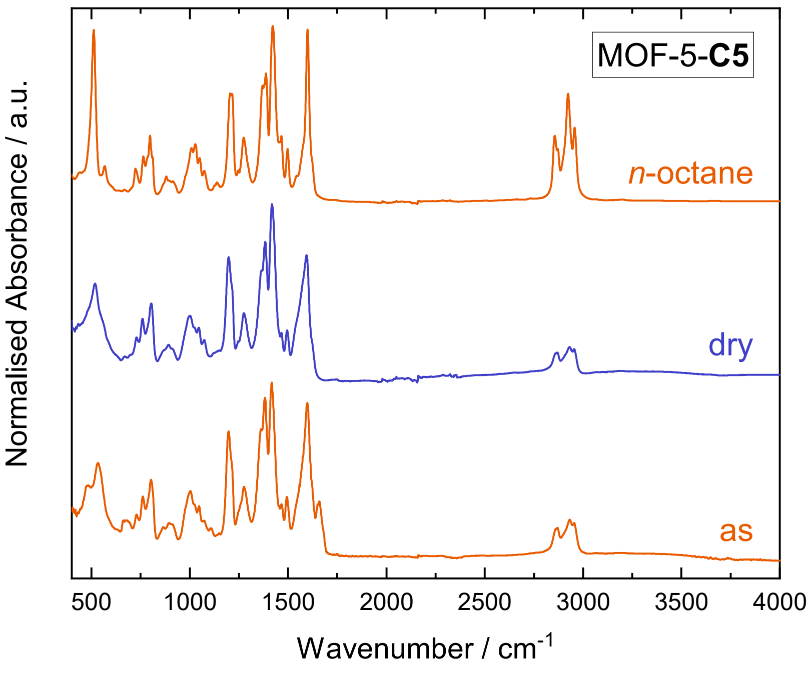

Supplementary Figure 6.4: FT-IR spectra of as-, dry- and n-octane reinfiltrated MOF-5-C5.

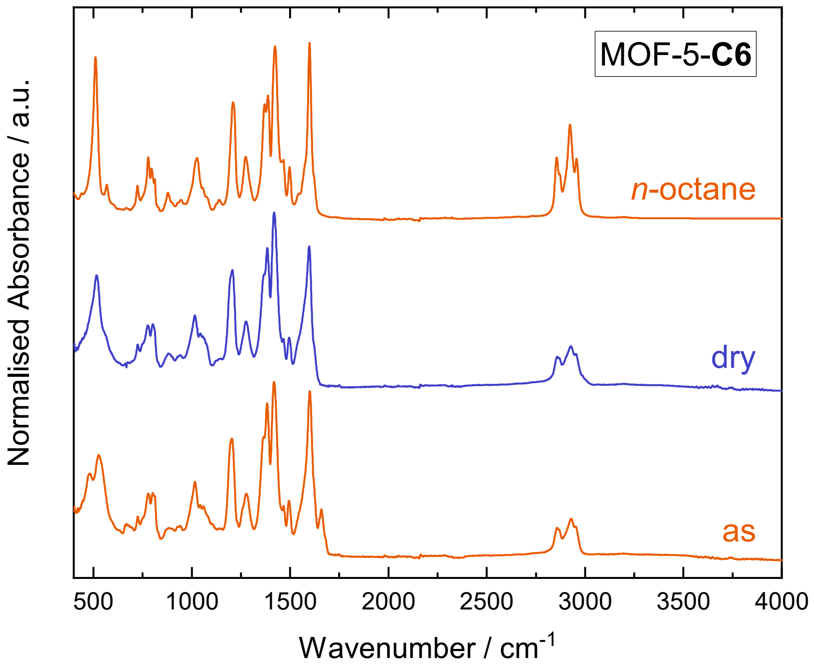

Supplementary Figure 6.5: FT-IR spectra of as-, dry- and n-octane reinfiltrated MOF-5-C6.

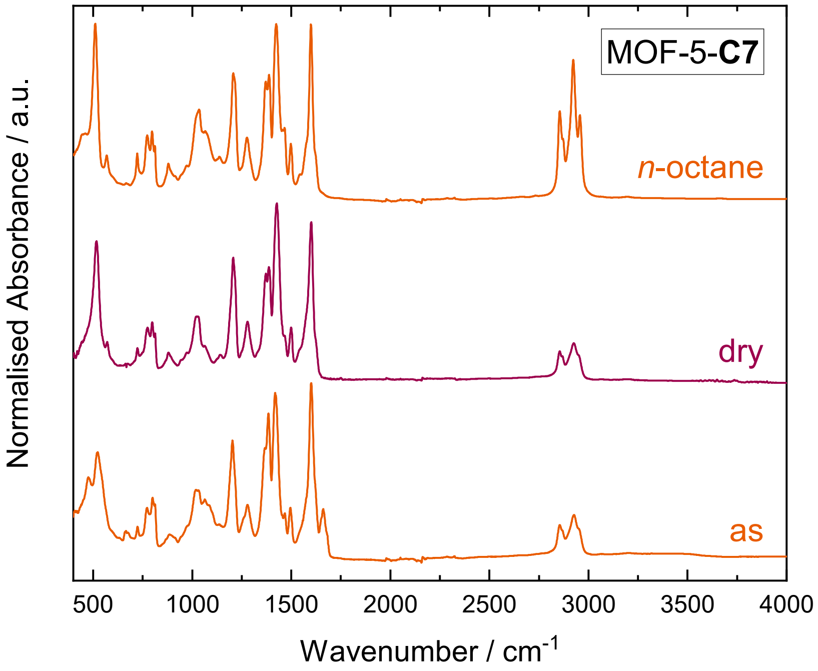

Supplementary Figure 6.6: FT-IR spectra of as-, dry- and n-octane reinfiltrated MOF-5-C7.

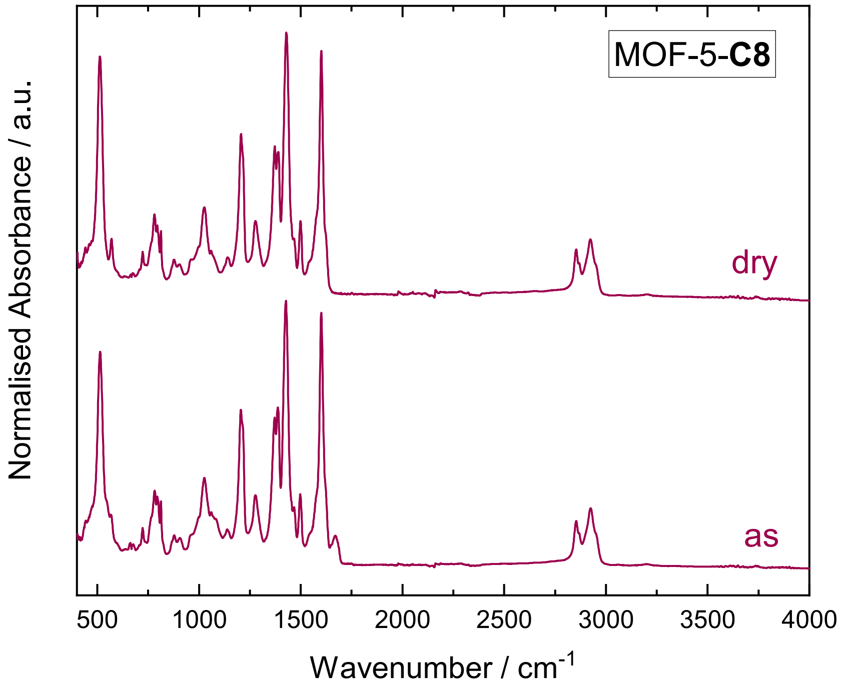

Supplementary Figure 6.7: FT-IR spectra of as- and dry-MOF-5-C8.

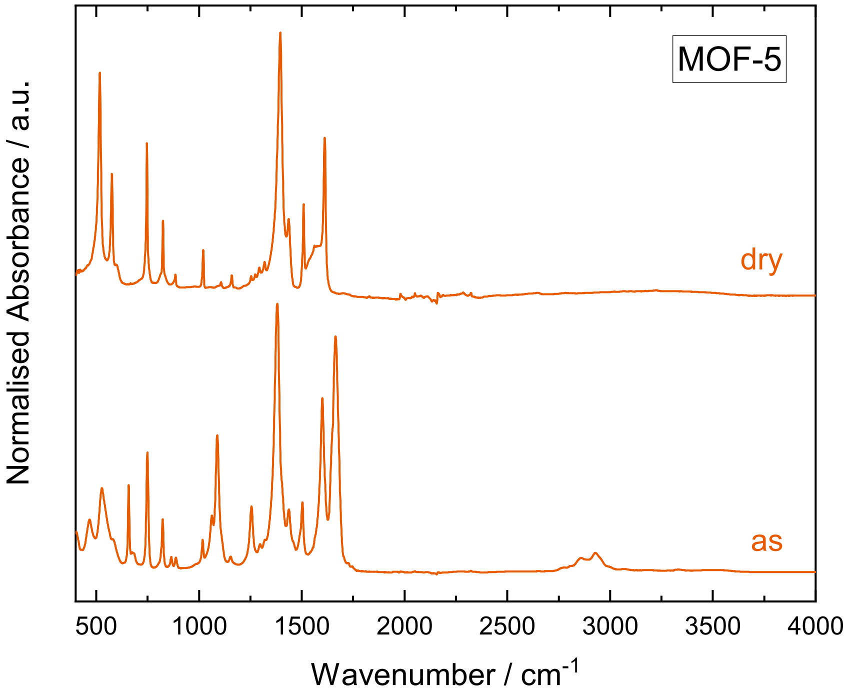

Supplementary Figure 6.8: FT-IR spectra of as-MOF-5 and dry-MOF-5 used for comparison.

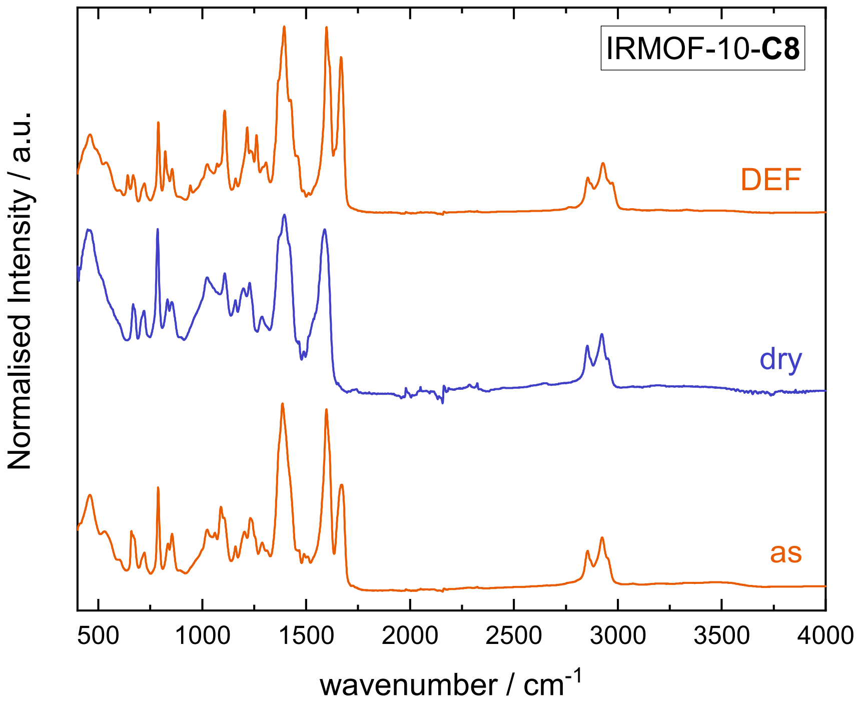

Supplementary Figure 6.9: FT-IR spectra of as-, dry- and DEF-IRMOF-10-C8.

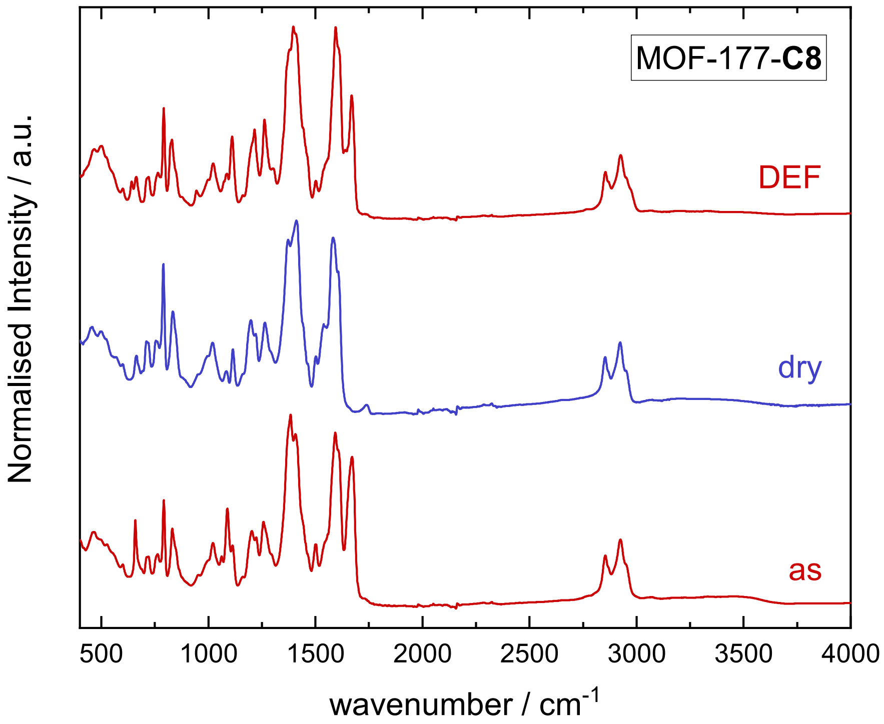

Supplementary Figure 6.10: FT-IR spectra of as-, dry- and DEF-MOF-177-C8.

Supplementary Methods 7 - Thermal analysis

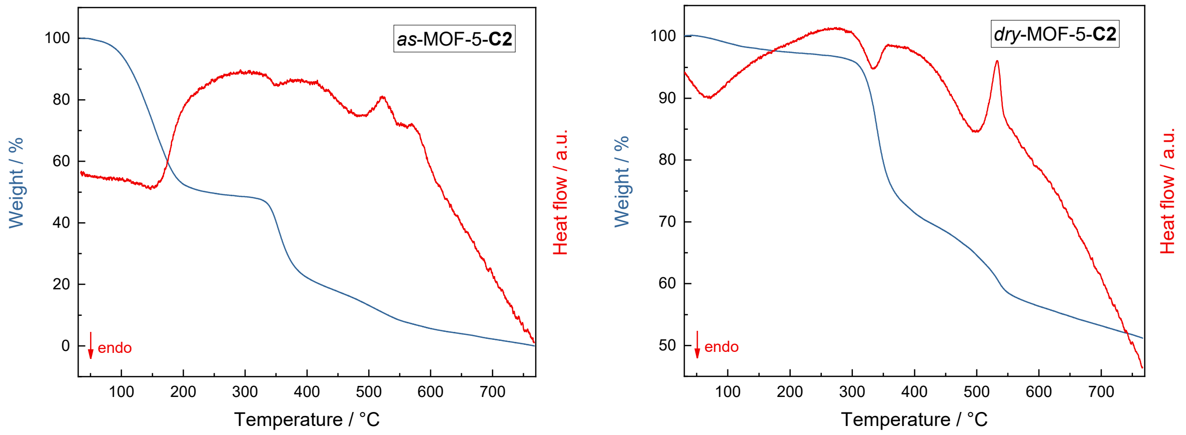

Supplementary Figure 7.1: TG-DSC data of as- (left) and dry-MOF-5-C2 (right). The small weight-loss of dry-MOF-5-C2 below 150 °C is ascribed to the release of moisture, adsorbed on the sample during preparation for the TG-DSC measurement in air.

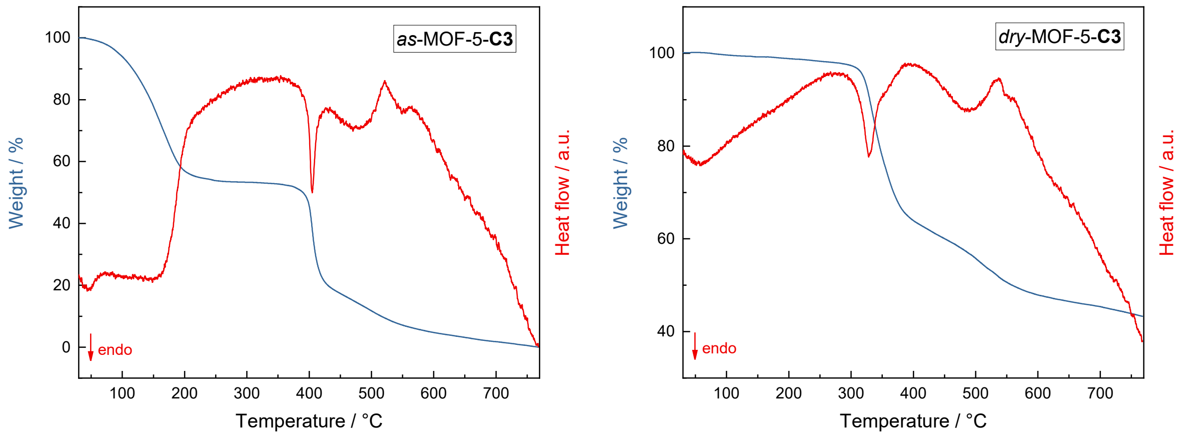

Supplementary Figure 7.2: TG-DSC data of as- (left) and dry-MOF-5-C3 (right).

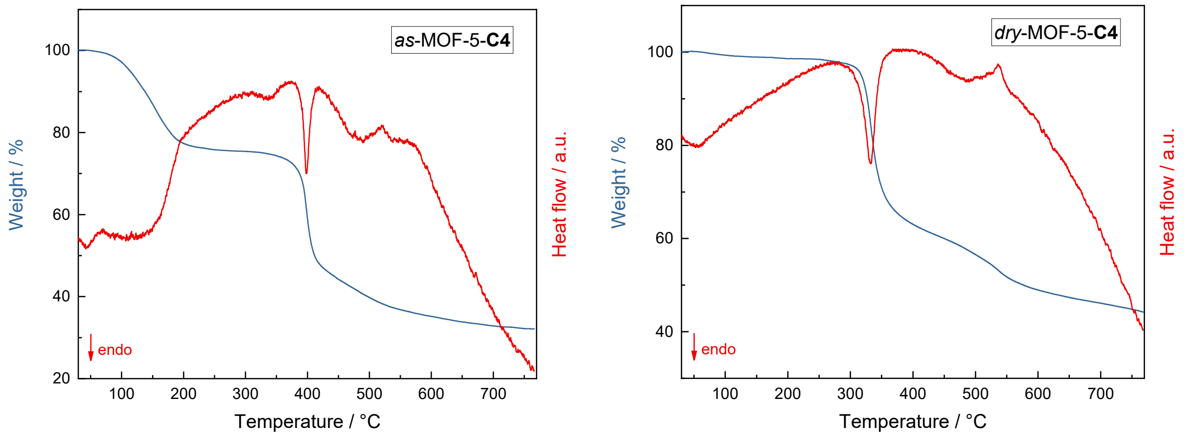

Supplementary Figure 7.3: TG-DSC data of as- (left) and dry-MOF-5-C4 (right).

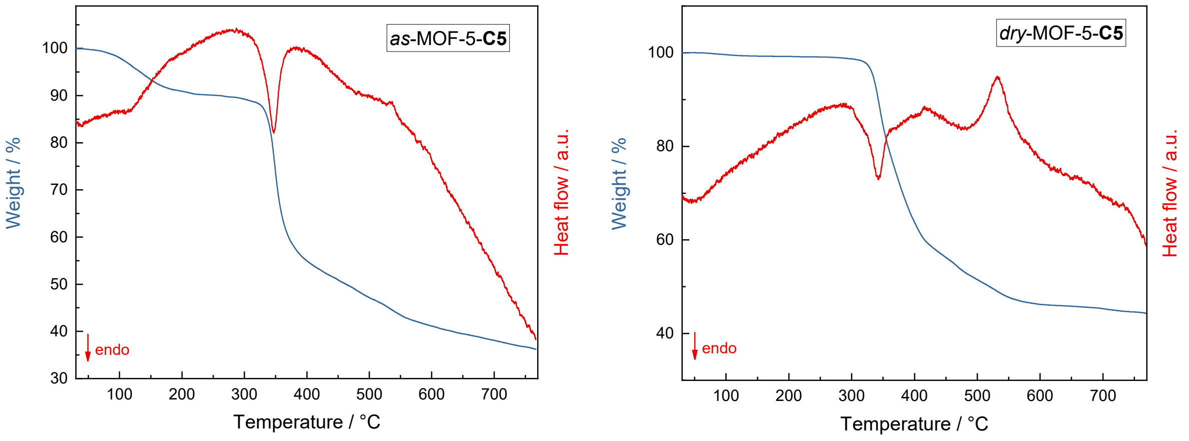

Supplementary Figure 7.4: TG-DSC data of as- (left) and dry-MOF-5-C5 (right).

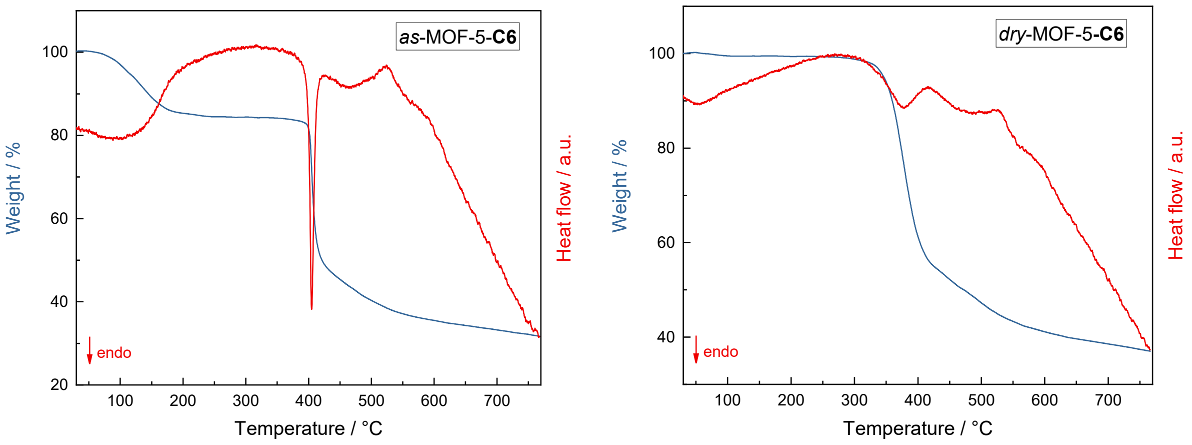

Supplementary Figure 7.5: TG-DSC data of as- (left) and dry-MOF-5-C6 (right).

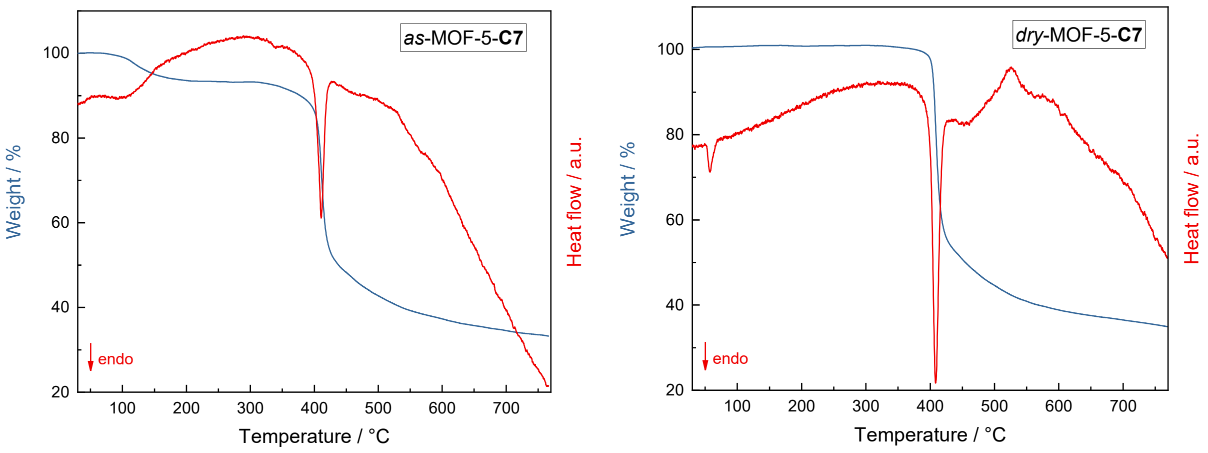

Supplementary Figure 7.6: TG-DSC data of as- (left) and dry-MOF-5-C7 (right).

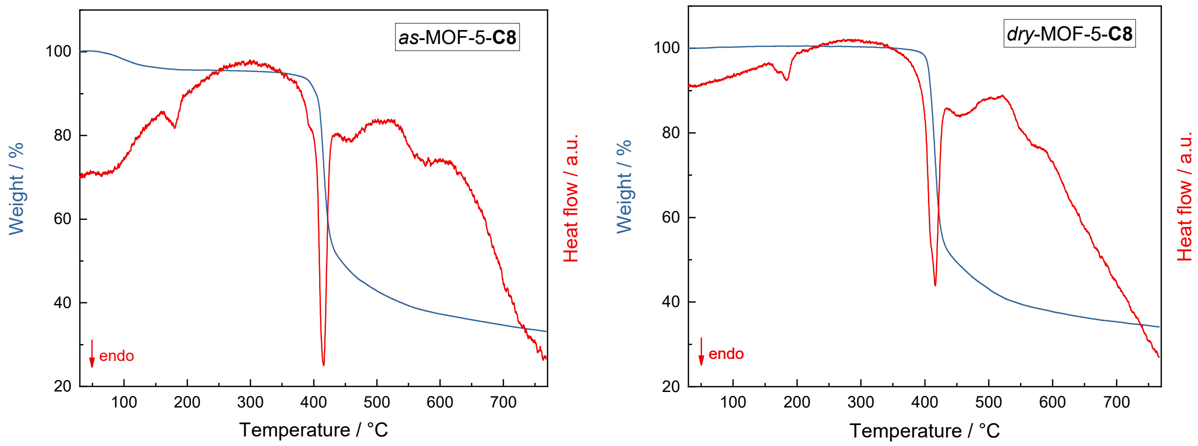

Supplementary Figure 7.7: TG-DSC data of as- (left) and dry-MOF-5-C8 (right).

From the TG-DSC data mentioned above, the decomposition temperatures of the dry-materials were extracted as the onset temperatures of the decomposition peak of the first derivative of the TG curve. The resulting values are listed below.

Supplementary Table 7.1: Decomposition temperatures of dry-MOF-5-CX obtained from TG-DSC.

Sample	Decomposition temperature / °C
MOF-5-C2	314
MOF-5-C3	314
MOF-5-C4	319
MOF-5-C5	327
MOF-5-C6	341
MOF-5-C7	407
MOF-5-C8	408

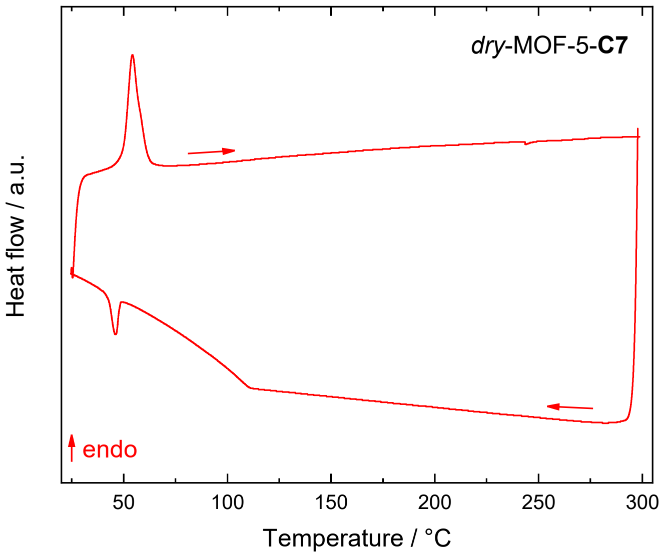

Supplementary Figure 7.8: DSC data of dry-MOF-5-C7 used for determination of $\Delta H_{rh\rightarrow c}$.

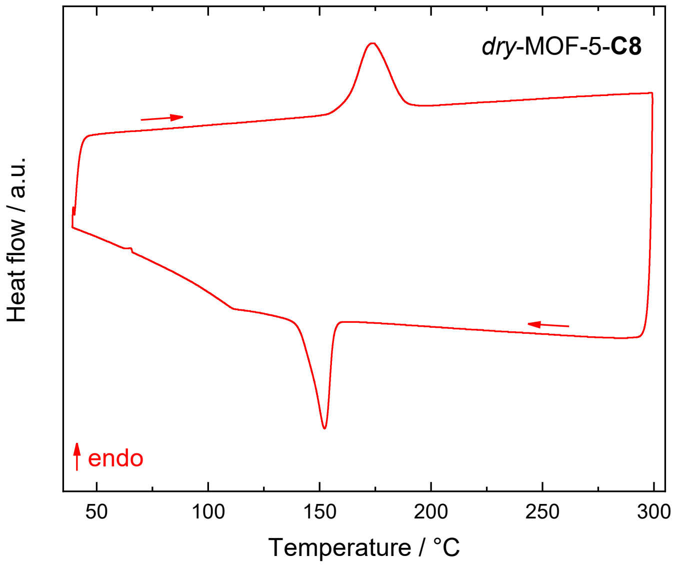

Supplementary Figure 7.9: DSC data of dry-MOF-5-C8 used for determination of $\Delta H_{rh\rightarrow c}$.

Supplementary Table 7.2: Thermodynamic data of dry-MOF-5-C7 and dry-MOF-5-C8 obtained from DSC data measured via simultaneous thermal analysis (STA). The phase transition temperatures are determined at the peak onset of the calorimetric signal.

**Sample**		ΔH **/ kJ mol^{-1}**	ΔS **/ J K^{-1} mol^{-1}**	$T_{rh\rightarrow c}$ **/** $T_{c\rightarrow rh}$ **/ °C**
dry-MOF-5-C7	upscan	9.7	30.1	50
	downscan	–7.7	–23.9	48
dry-MOF-5-C8	upscan	23.1	53.2	162
	downscan	–19.8	–46.0	156

Supplementary Methods 8 - Variable temperature PXRD

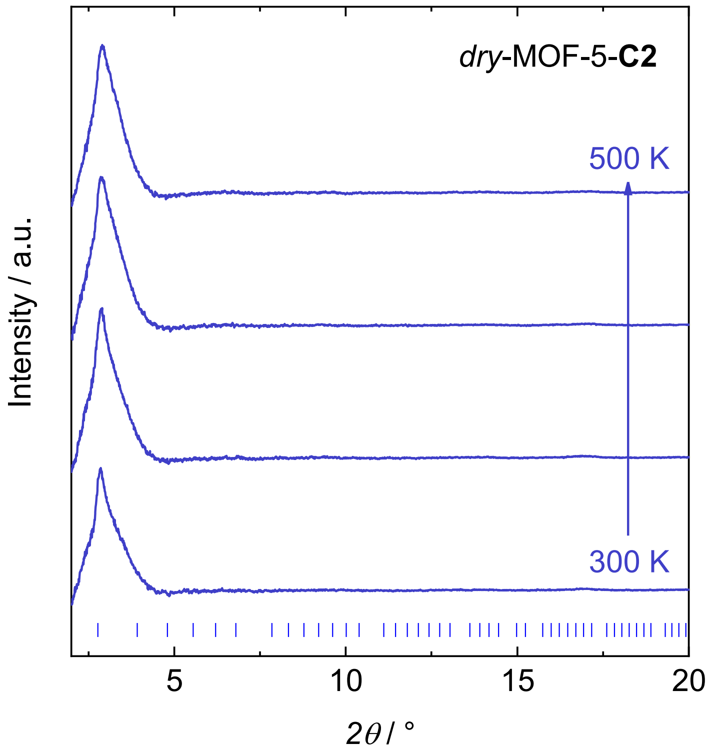

Supplementary Figure 8.1: VT-PXRD patterns recorded of a dried sample of MOF-5-C2 in the range from 300 – 500 K (DELTA, λ = 0.620 Å). Tick marks correspond to positions of allowed Bragg reflections of the corresponding as-synthesized phase of the MOF.

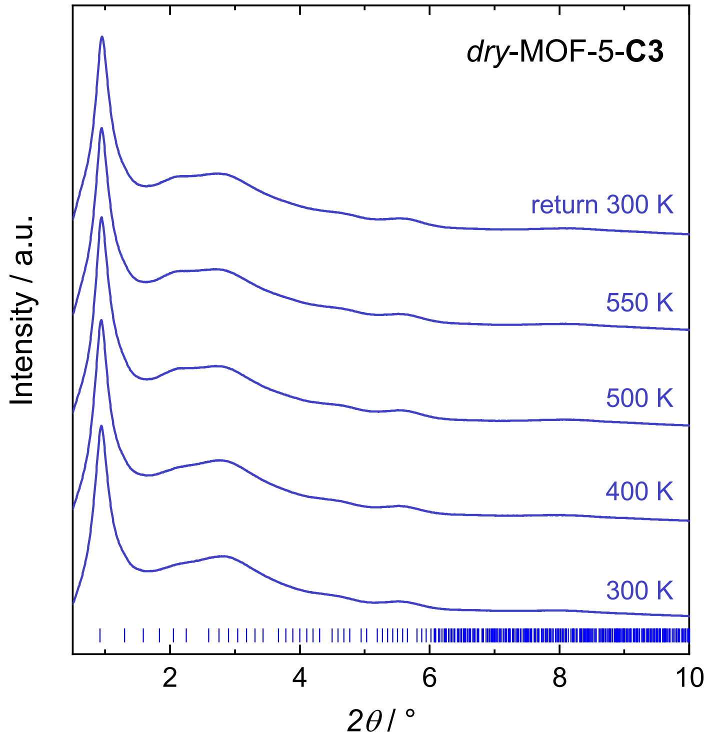

Supplementary Figure 8.2: VT-PXRD patterns recorded of a dried sample of MOF-5-C3 in the range from 300 – 550 K (P02.1 of DESY, λ = 0.207 Å). Tick marks correspond to positions of allowed Bragg reflections of the corresponding as-synthesized phase of the MOF.

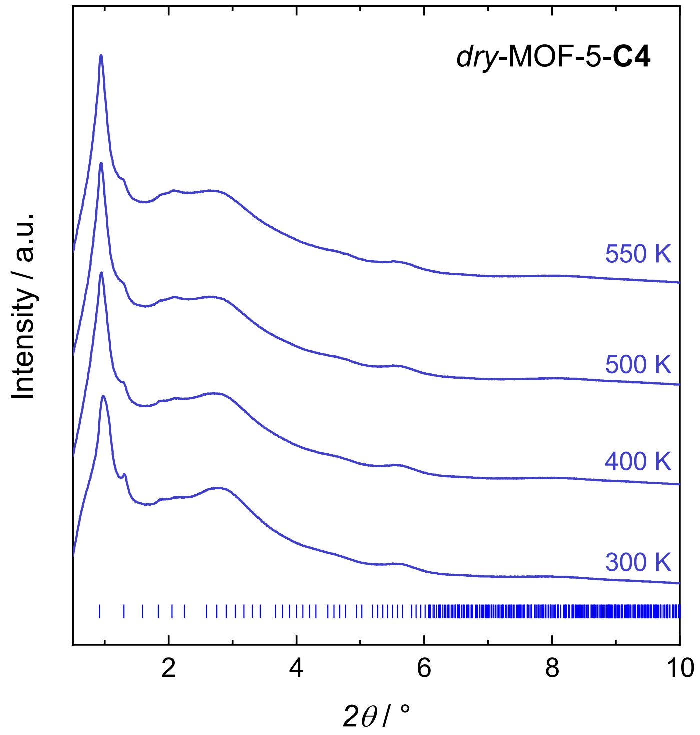

Supplementary Figure 8.3: VT-PXRD patterns recorded of a dried sample of MOF-5-C4 in the range from 300 – 550 K (P02.1 of DESY, λ = 0.207 Å). Tick marks correspond to positions of allowed Bragg reflections of the corresponding as-synthesized phase of the MOF.

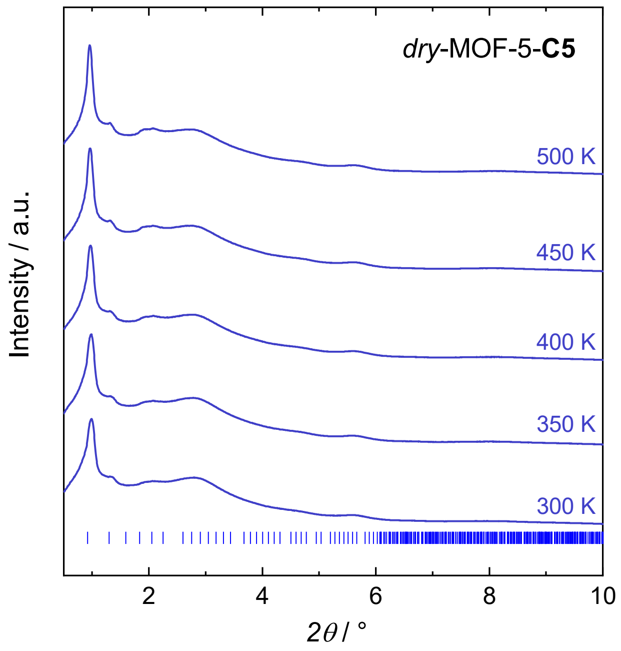

Supplementary Figure 8.4: VT-PXRD patterns recorded of a dried sample of MOF-5-C5 in the range from 300 – 500 K (P02.1 of DESY, λ = 0.207 Å). Tick marks correspond to positions of allowed Bragg reflections of the corresponding as-synthesized phase of the MOF.

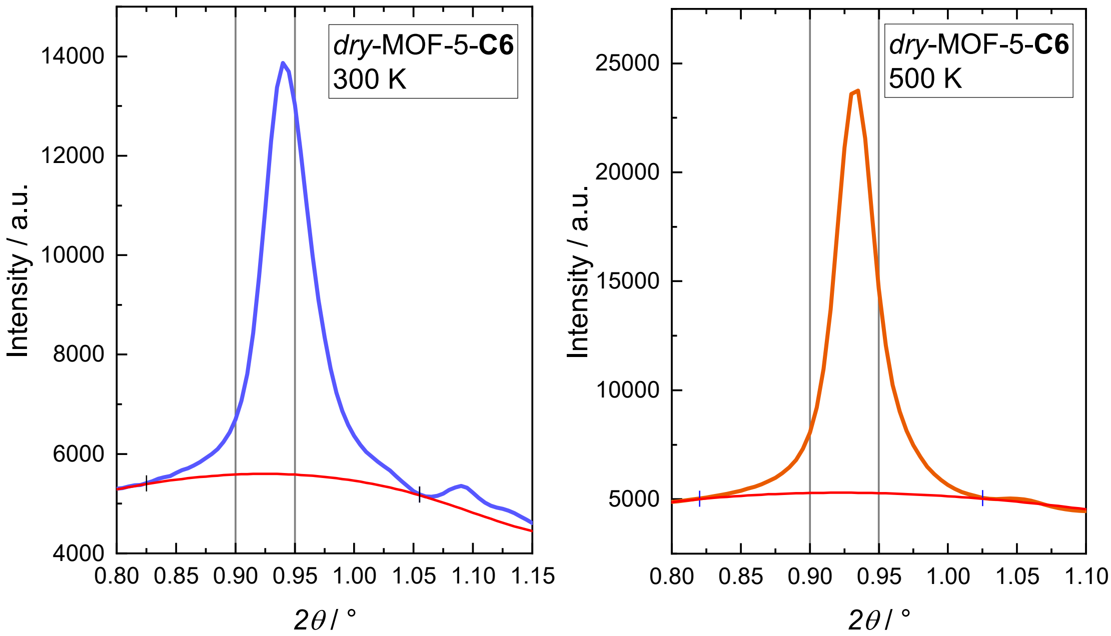

Supplementary Figure 8.5: Integrals of the FSP of dry-MOF-5-C6 at 300 and 500 K (P02.1 of DESY, λ = 0.207 Å). PXRD data and baseline used for the calculation of the integral of the low-*Q* peak in dry-MOF-5-C6 at 300 K (left) and 500 K (right). Grey lines at 0.90° and 0.95° were added as a guide to the eye to elucidate the shift of the peak maximum. The calculation of the integrals of the first scattering peak (FSP) of dry-MOF-5-C6 was performed using the ORIGIN Pro 2020 software package. The diffraction patterns are not normalized and have been collected on the same sample with the very same exposure time. Previously subtracted baselines and used diffraction data are depicted in the figures given below.

Supplementary Table 8.1 Integral, FWHM values and the maximum of the FSP of dry-MOF-5-C6 at different temperatures at identical exposure times of the same sample.

T / K	*Integral* / a.u.	*FWHM* / °	$2\theta_{FSP}$	d_{FSP}
300	464.7	0.060	0.945	12.57
500	797.5	0.023	0.933	12.73

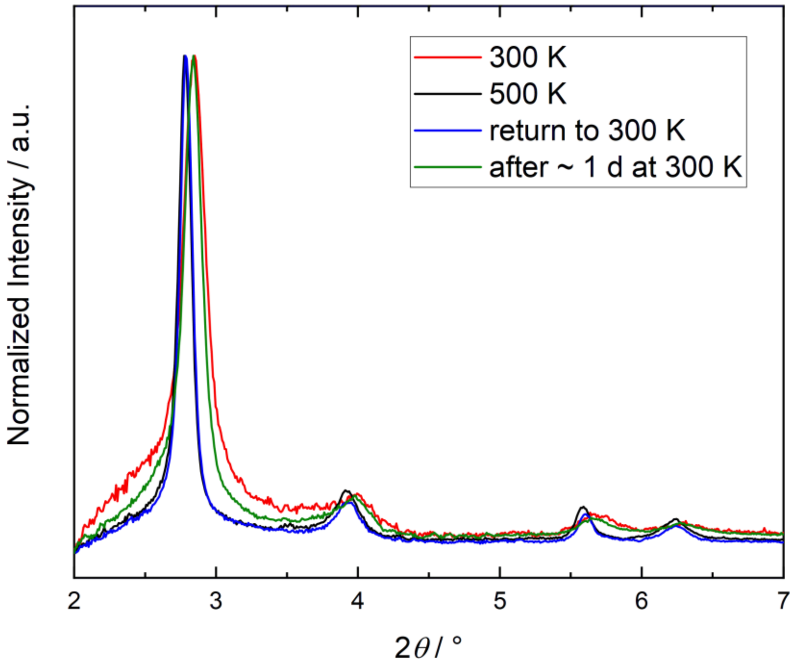

Supplementary Figure 8.6: Normalized PXRD patterns of dry-MOF-5-C6 recorded at different temperatures (BL9 of DELTA, λ = 0.620 Å). Following heating the sample to 500 K, it does not immediately return to its initial structure after cooling back to 300 K. However, after letting the sample rest for ~ 1 d at room temperature, the PXRD pattern is very similar to the one recorded prior to the heat treatment. This signifies that the kinetics of the transformation from the crystalline into the non-crystalline, distorted state are rather slow.

Supplementary Methods 9 - Isothermal gas sorption

Supplementary Methods 9.1 - N_2 sorption

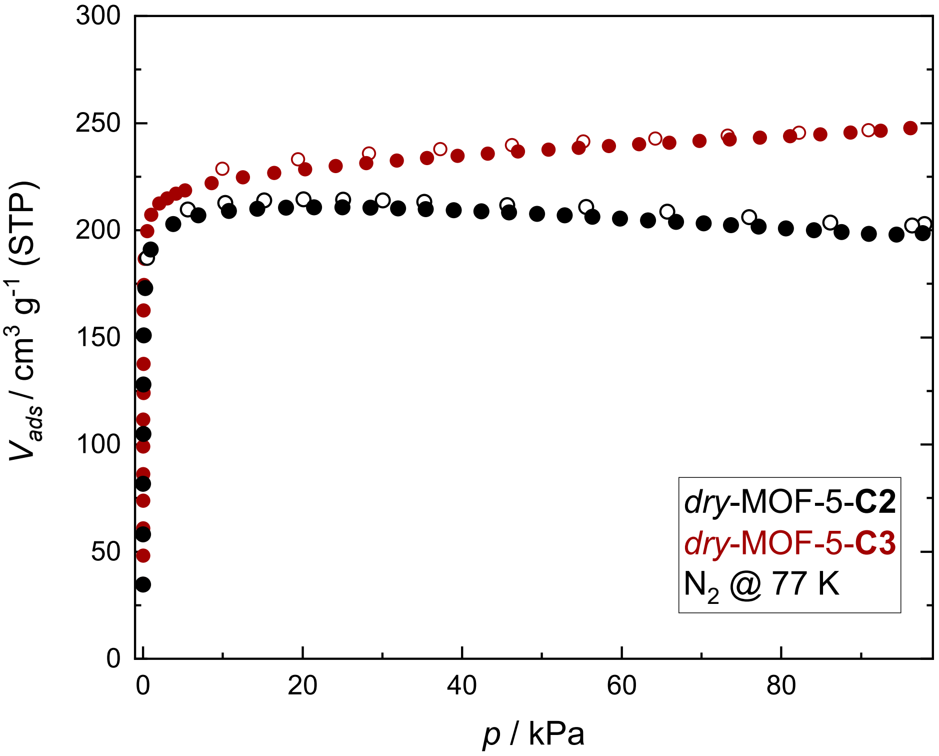

Supplementary Figure 9.1: N_2 sorption isotherms of dry-MOF-5-C2 and dry-MOF-5-C3 recorded at 77 K. Adsorption and desorption branches are shown with closed and open symbols. Please note, that the drop of the N_2 uptake in the range from 30 to 100 kPa in the isotherm of dry-MOF-5-C2 is likely caused by one of the two following scenarios: (i) An error in the void volume measurement or (ii) a small leak in the measurement cell (microcrack in the glass or a faulty sealing ring). The drop in uptake amounts to about 5%. Due to this measurement error, the Langmuir and BET surface areas of dry-MOF-5-C2 (see Supplementary Table 9.1) are unreliable. Nevertheless, our scientific discussion is solely based on the quantitative results of the CO_2 isotherms (Supplementary Figure 9.3 and Supplementary Table 9.2) and thus not compromised by this issue.

Supplementary Table 9.1: Langmuir and BET surface areas of dry-MOF-5-C2 and dry-MOF-5-C3 calculated from the isothermal N_2 sorption data. For the surface areas of dry-MOF-5-C2 see the comment above.

	$S_{Langmuir}$ / m^{2}g^{-1}	S_{BET} / m^{2}g^{-1}
dry-MOF-5-C2	919	842
dry-MOF-5-C3	1159	911

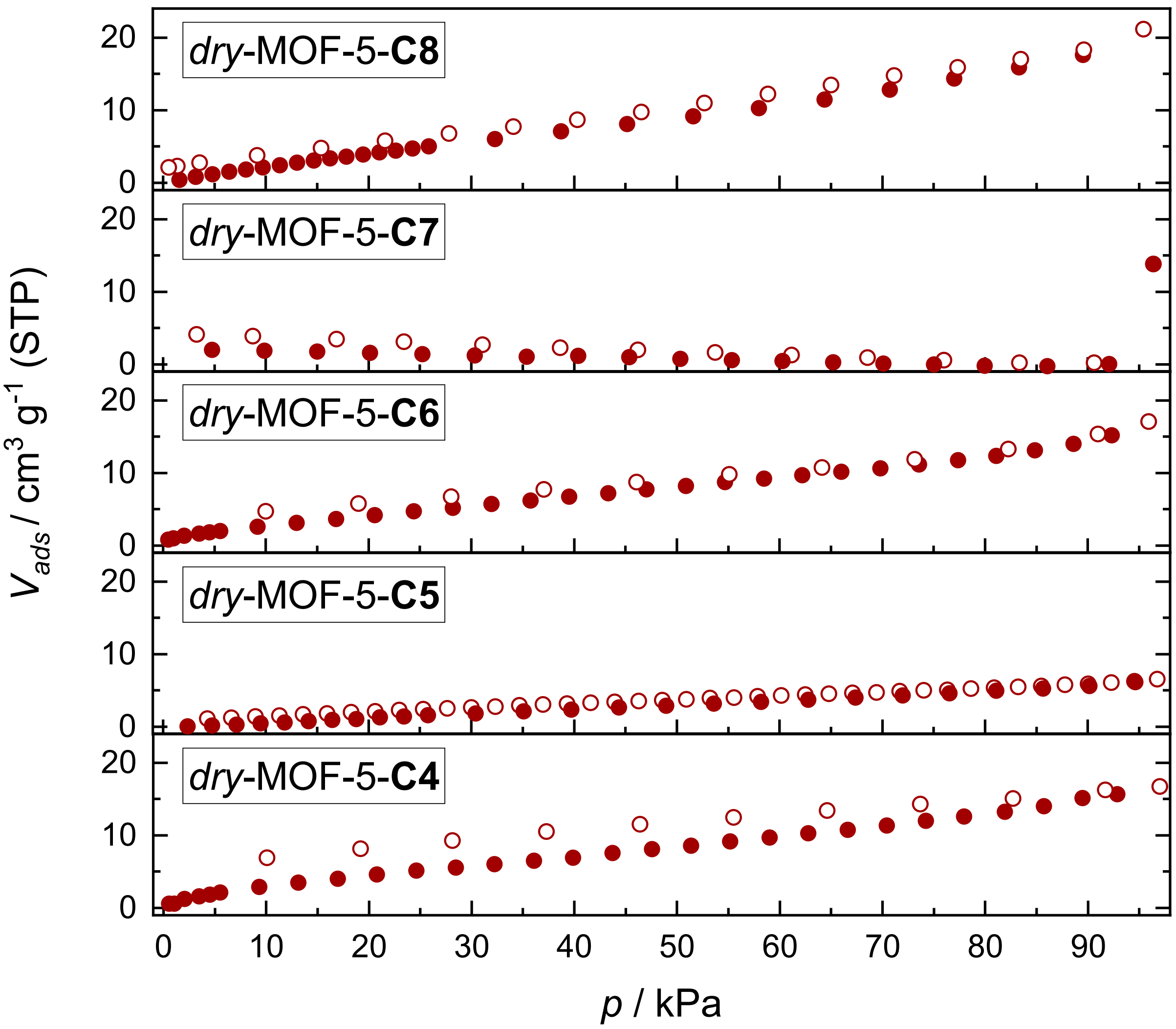

Supplementary Figure 9.2: N_2 sorption isotherms of dry-MOF-5-C4 to dry-MOF-5-C8 recorded at 77 K. Adsorption and desorption branches are shown with closed and open symbols.

Supplementary Methods 9.2 - CO_2 sorption

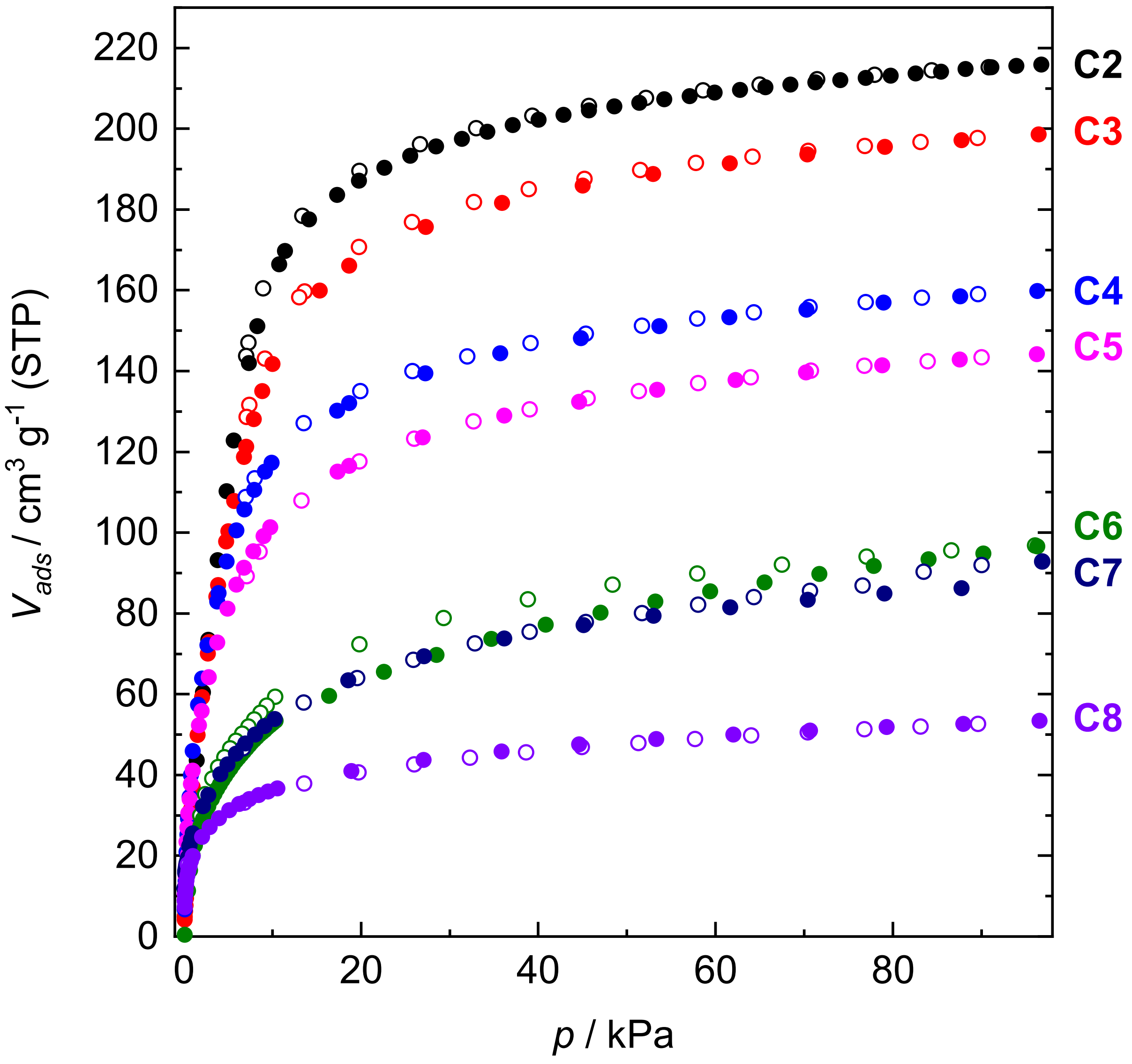

Supplementary Figure 9.3: CO_2 sorption isotherms of dry-MOF-5-C2 to dry-MOF-5-C8 recorded at 195 K. Adsorption and desorption branches are shown with closed and open symbols.

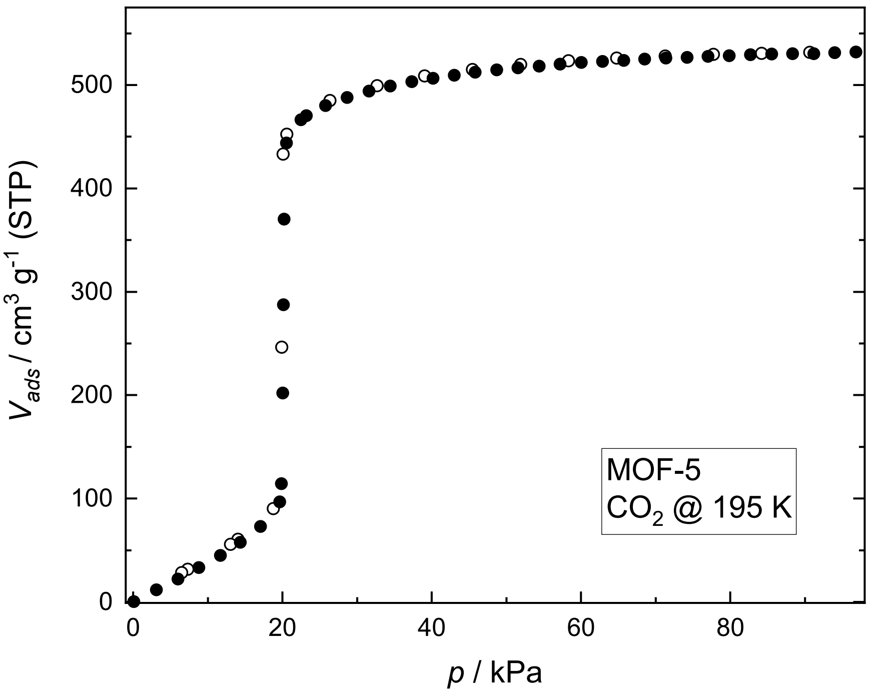

Supplementary Figure 9.4: CO_2 sorption isotherms of MOF-5 recorded at 195 K. Adsorption and desorption branches are shown with closed and open symbols.

Supplementary Table 9.2.: Langmuir and BET surface areas of dry-MOF-5-C2 to dry-MOF-5-C8 and MOF-5 were calculated from the isothermal (195 K) CO_2 sorption isotherms. In accordance to previous reports, a molecular cross sectional area σ = 17.00 Å^2 was used.[18] For the calculation of the BET surface areas three data points between 0.014 and 0.05 (MOF-5-C3 to C8) or 0.07 and 0.11 (MOF-5-C2) p/p_0 (p_0 = 100 kPa) were taken[19]. The BET calculation has not been performed for MOF-5 because of the unusual shape of its CO_2 isotherm.

	$S_{Langmuir}$ / m^{2}g^{-1}	S_{BET} / m^{2}g^{-1}
dry-MOF-5-C2	1041	895
dry-MOF-5-C3	978	731
dry-MOF-5-C4	797	559
dry-MOF-5-C5	697	481
dry-MOF-5-C6	489	235
dry-MOF-5-C7	427	237
dry-MOF-5-C8	258	159
MOF-5	2534	-

Determination and evaluation of total pore volumes

To determine the volume contraction of the dry-MOF-5-CX phases, we compared the experimentally determined total pore volumes of the dry-MOF-5-CX phases (V_{exp}) to the expected pore volumes of a respective theoretical cubic framework (V_{theo}). The discrepancy between V_{exp} and V_{theo}, was then related to the crystallographic volume of the cubic phases, $V_{cryst,\text{cub}}$, of the as-MOF-5-CX in order to estimate how much pore volume has been lost for the dry-MOF-5-CX materials as a consequence of non-correlated framework contraction.
In order to do so, experimental total pore volumes (V_{exp}) were calculated from the maximum CO_2 uptake (in cm^3 CO_2 / mol MOF at standard temperature and pressure) at pressures close to p/p_0 = 0.95 (i.e. 95 kPa) by applying a liquid density of $\rho(CO_2)$ = 1.032 g cm^{-3}.[18] These calculations were undertaken by use of the *ASiQwin* (Version 5.2) software package. V_{theo} was obtained from a linear interpolation (see Fig. 5b in the main article, dashed line) of a plot of the experimental pore volume vs. the Connolly solvent excluded volume (CSEVs) of the alkoxy side chains of MOF-5 and dry-MOF-5-C8. The latter was used, because it shows a relatively small volume contraction (approx. 1.5% compared to the cubic phase). Subtracting V_{exp} from V_{theo} gives ΔV. ΔV is the 'missing' pore volume per mole MOF of the dry-MOF-5-CX (X = 2 - 6) phases. Relating ΔV to the crystallographic volume per mole as-MOF-5-CX, V_{cubic}, as known from PXRD, yields an estimate for the degree of contraction of the dry-MOF-5-CX phases. Results are listed in Supplementary Table 9.3.

The CSEVs of the alkoxy substituents used here were calculated using the routines implemented in the Perkin Elmer *Chem3D* software with a probe radius r = 1.4 Å. At this, benzene was used as a model compound, to which at first two ethoxy moieties were added in 2,5-positions, that were successively elongated by CH_2-groups at both arms. For each step, the resulting CSEV was obtained and the respective volume of benzene was subtracted from it. By this procedure, the space requirement of the alkoxy chains of each length was determined for one repeating unit of the framework.

Supplementary Table 9.3: Estimation of the volume contraction from CO_2 sorption experiments. V_{exp} is the experimental pore volume per mole MOF. V_{theo} is the theoretical pore volume of a hypothetical crystalline cubic MOF-5-CX derived via interpolation (see text above). $\Delta V = V_{theo} - V_{exp}$. $V_{cryst,cub}$ is the crystallographic volume of the corresponding cubic as-MOF-5-CX phase. %change = $\Delta V / V_{cryst,cub}$.

X	V_{exp} / cm^3mol^{-1}	V_{theo} / cm^3mol^{-1}	ΔV / cm^3mol^{-1}	$V_{cryst,cub}$ / cm^3mol^{-1}	%change
**2**	425	611	186	1297	-14
**3**	423	536	113	1303	-9
**4**	366	461	95	1306	-7
**5**	353	386	33	1299	-3
**6**	253	311	58	1300	-4

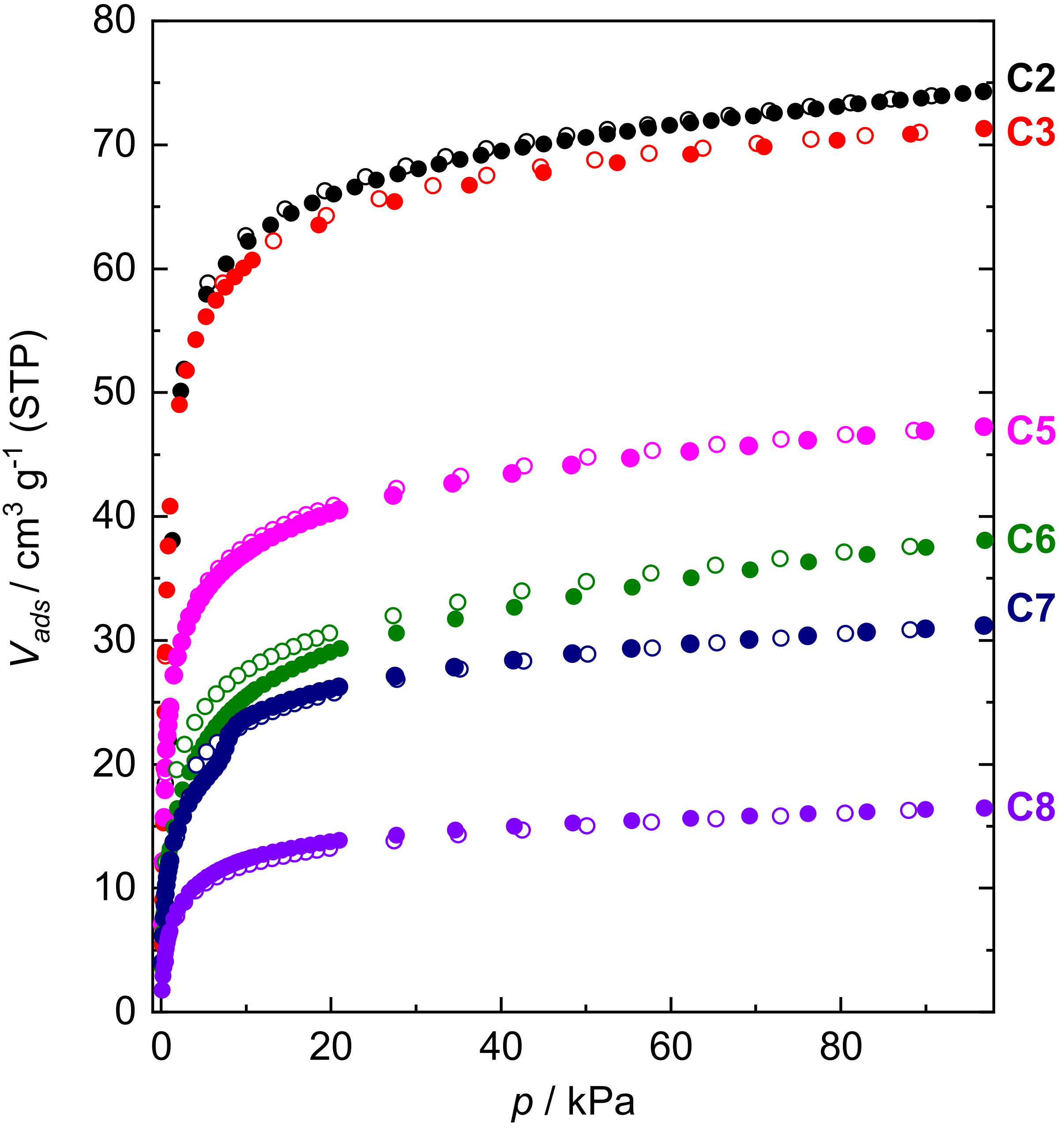

Supplementary Figure 9.5: n-Butane sorption isotherms of dry-MOF-5-CX derivatives recorded at 293 K. Adsorption and desorption branches are shown with closed and open symbols.

Supplementary Methods 10 - In situ gas sorption PXRD

Supplementary Methods 10.1 - In situ CO_2 sorption PXRD

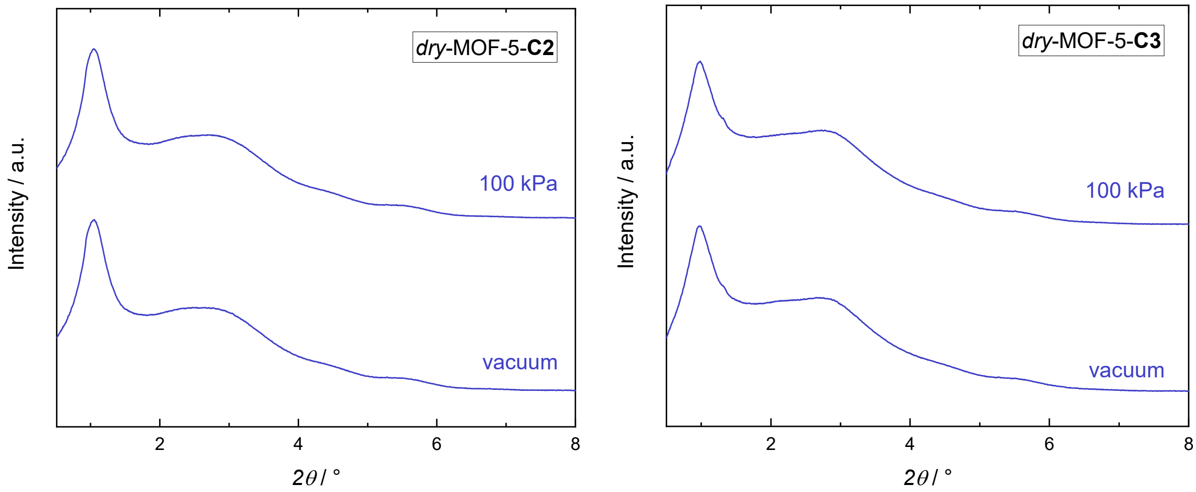

Supplementary Figure 10.1: In situ CO_2 sorption PXRD patterns of dry-MOF-5-C2 and dry-MOF-5-C3 recorded at 195 K.

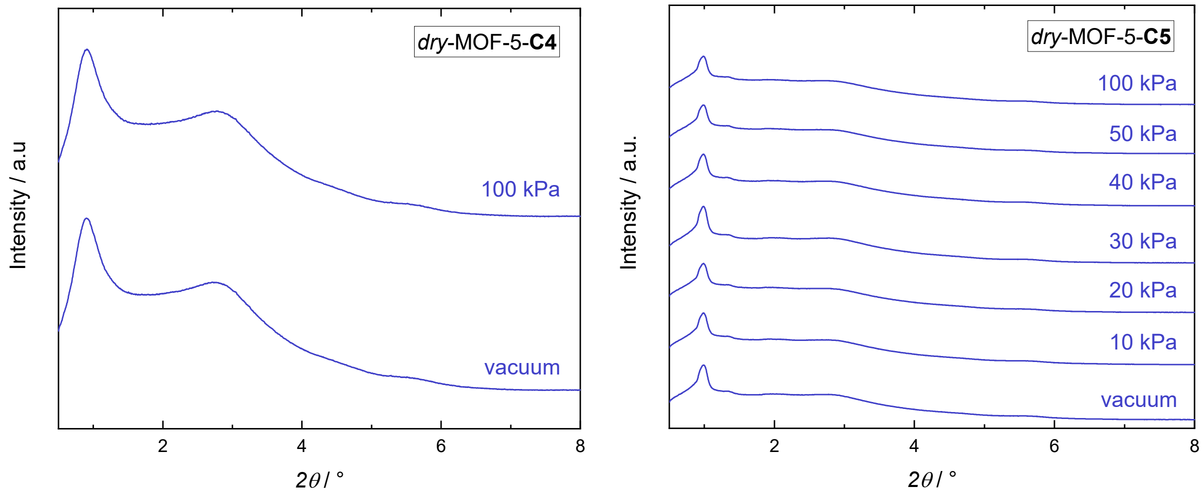

Supplementary Figure 10.2: In situ CO_2 sorption PXRD patterns of dry-MOF-5-C4 and dry-MOF-5-C5 recorded at 195 K.

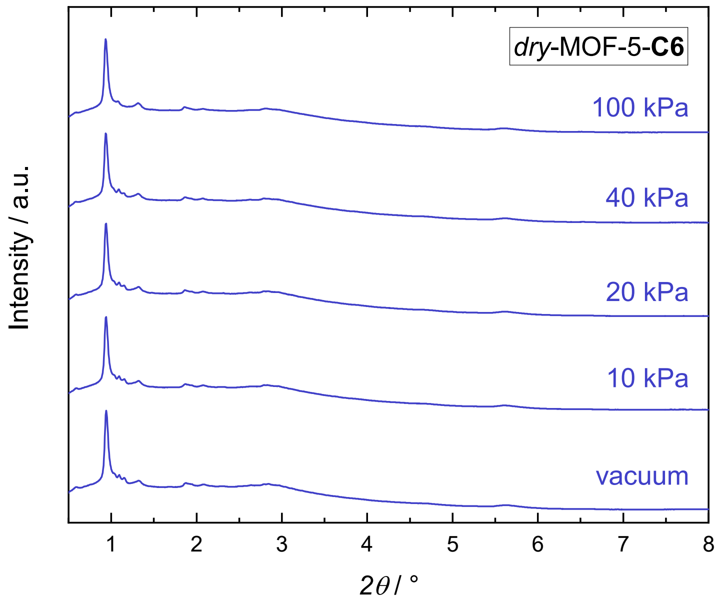

Supplementary Figure 10.3: In situ CO_2 sorption PXRD patterns of dry-MOF-5-C6 recorded at 195 K.

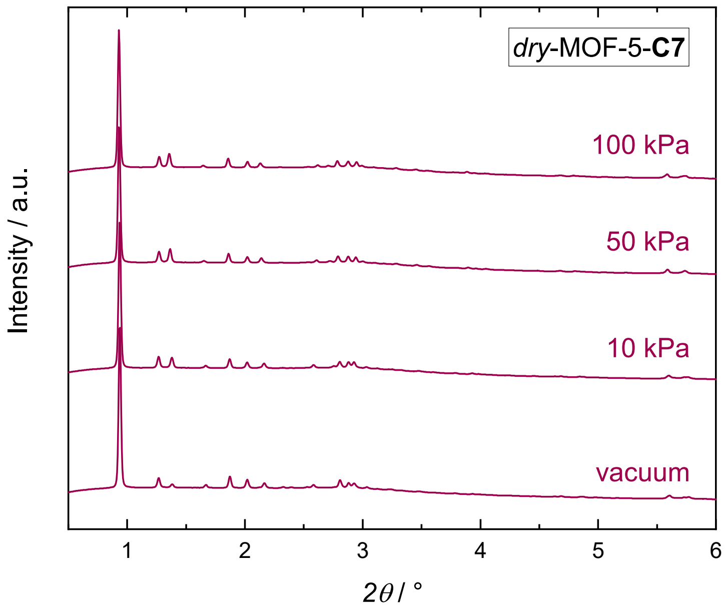

Supplementary Figure 10.4: In situ CO_2 sorption PXRD patterns of dry-MOF-5-C7 recorded at 195 K.

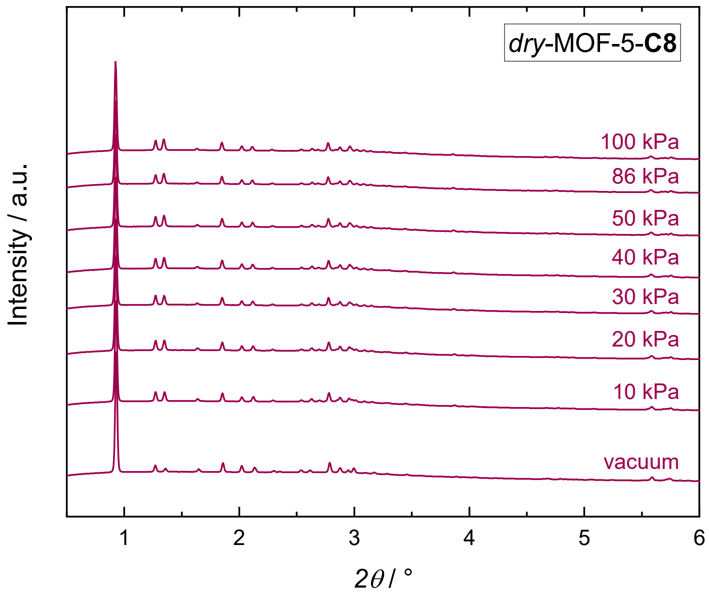

Supplementary Figure 10.5: In situ CO_2 sorption PXRD patterns of dry-MOF-5-C8 recorded at 195 K.

Supplementary Methods 10.2 - In situ n-butane sorption PXRD

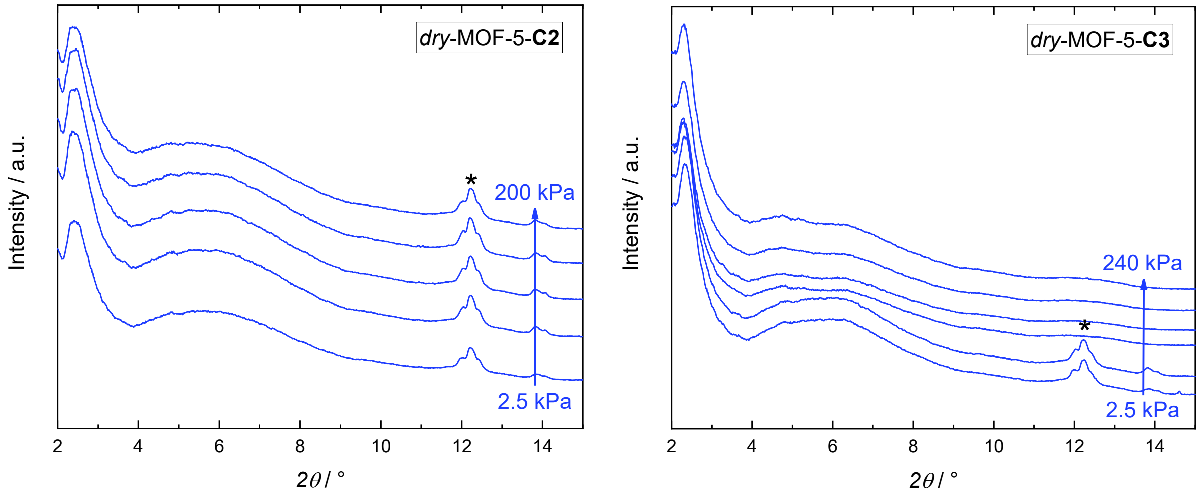

Supplementary Figure 10.6: In situ n-butane sorption PXRD patterns of dry-MOF-5-C2 and dry-MOF-5-C3 recorded at 298 K. Marked reflections (*) result from the gas cell being hit by the X-ray beam.

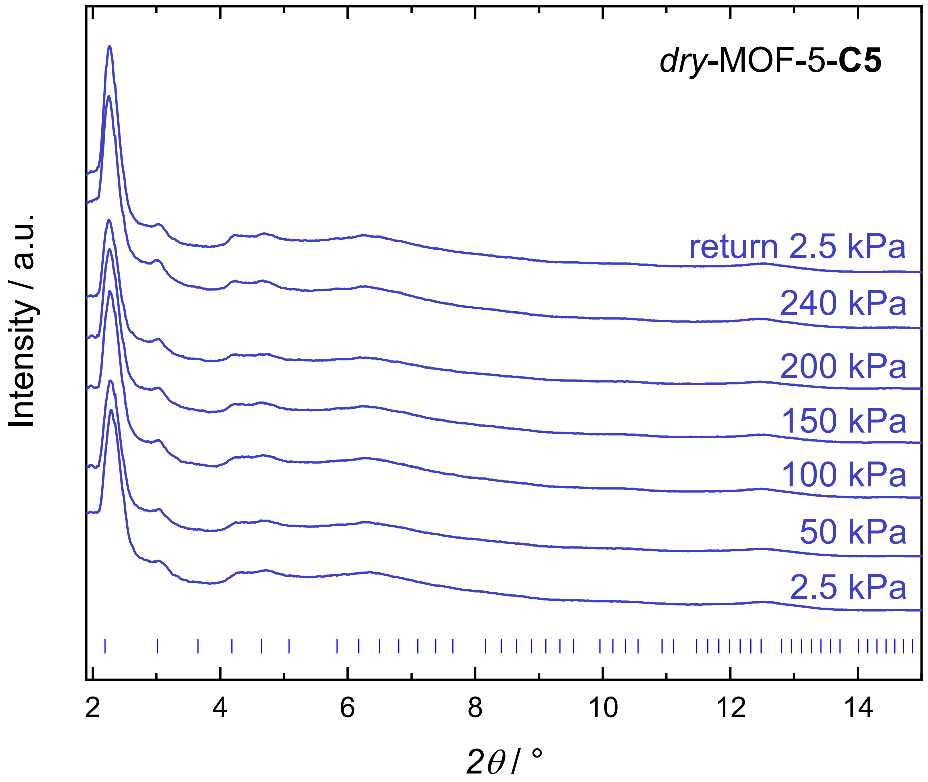

Supplementary Figure 10.7: In situ n-butane sorption PXRD patterns of dry-MOF-5-C5 recorded at 298 K (λ = 0.460 Å). Tick Marks correspond to positions of allowed Bragg reflections of the corresponding as-synthesized phase of the MOF.

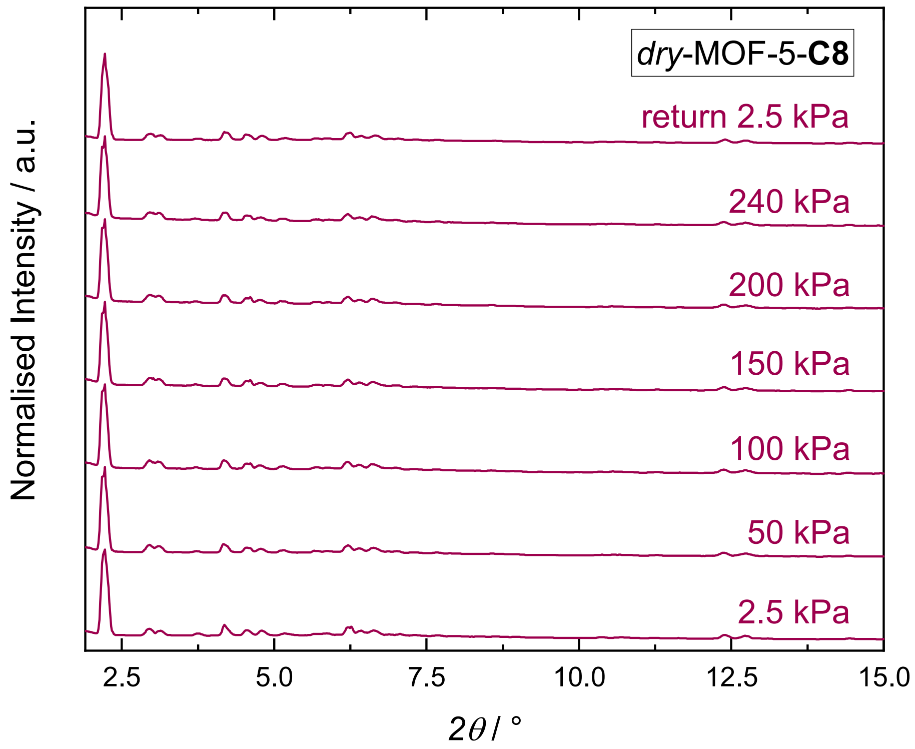

Supplementary Figure 10.8: In situ n-butane sorption PXRD patterns of dry-MOF-5-C8 recorded at 298 K (λ = 0.460 Å).

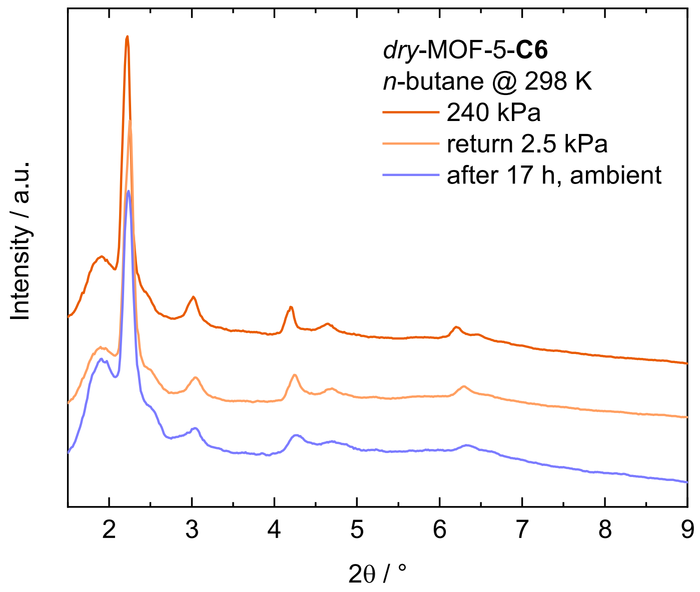

Supplementary Figure 10.9: In situ n-butane sorption PXRD patterns of dry-MOF-5-C6 recorded at 298 K (λ = 0.460 Å, DELTA). The first pattern was recorded at 240 kPa n-butane pressure. The second directly after returning to 2.5 kPa (the maximum accessible vacuum in this experiment) and after 17 h storage under ambient conditions. Similar to the variable temperature experiments (Supplementary Figure 8.6), the relaxation from the crystalline to the non-crystalline phase of dry-MOF-5-C6 is kinetically hindered and thus requires at least a few hours.

Supplementary Methods 11 - Molecular dynamics simulations

Preparation of the initial structures

Initial structural models used as input for the simulations are derived from the rhombohedral single crystal structure of as-MOF-5-C8 by successively cropping off each terminal methyl group and replacing it with a hydrogen atom. Before that, these MOF-5-CX structures were embedded in a primitive cubic unit cell containing one formula unit $Zn_4O(CX\text{-}bdc)_3$ (edge length ~ 12.9 Å)

The Zn_4O disorder present in the crystal structure was resolved manually by picking one of the two realizations of the Zn_4O tetrahedra and the corresponding carboxylic oxygen atoms.

A 2x2x2 supercell (i.e. a cell containing eight formula units, edge length ~ 25.81 Å) was constructed upon book-keeping of the atom indices that give rise to the one or the other cluster orientation, such that neighboring Zn_4O clusters are related by inversion symmetry as in the parent MOF-5 material. At the early stage of this investigation, we considered also the variant, where neighboring Zn_4O tetrahedra are related by translational symmetry. Due to the experimental SCXRD evidence (see Supplementary Methods 3.1) suggesting the vast majority of Zn_4O clusters to be related by inversion symmetry, however, we chose not to report these results herein. As a general trend, we found in our simulations, that the variant with translational symmetry possesses a slightly stronger tendency towards the distorted forms (based on the same small cell simulations as reported below). After the construction, process, all structures were lattice optimized keeping the cell angles fixed at 90.0 ° in order to enforce a cubic structure.

Pressure ramp NPT simulations

During initial ambient pressure NPT simulations we found that all simulated materials except for MOF-5-C8 remain in their cubic forms without any apparent structural transformation. We therefore enforce a phase transition by an external stimulus. An elevated pressure has recently been widely used to enforce such transformations,[20–24] which is why we use it here as well.

In contrast to the established simulation methodology, we slightly altered the protocol herein by adding a negative pressure ramp in the first simulation step to guide the formerly rhombohedral (although transformed to cubic) crystal structures to be cubic. In these pressure ramp NPT simulations, the initial structures for the free energy calculation are generated. Additionally, temperature ramp simulations were performed starting from the compressed structure obtained from the NPT simulations. The simulation protocol to enforce a pressure induced cubic-to-rhombohedral phase transition is composed of five steps:

- Velocity equilibration, 250 ps *NVT* @ 300 K. Nosé-Hoover Thermostat
- Cell shape equilibration, 250 ps $NV(\sigma_a=0)T$ @ 300 K, MTTK Barostat
- Negative pressure ramp, 5 ns $NP(\sigma_a=0)T$, p_0= 0.0 GPa, p_1=-0.5 GPa
- Positive pressure ramp, 5 ns $NP(\sigma_a=0)T$, p_0=-0.5 GPa, p_1=0.0 GPa,
- Production pressure ramp, 10 ns $NP(\sigma_a=0)T$, p_0= 0.0 GPa, p_1=1.0 GPa

The pressure gradient of dp/dt = ±0.1 GPa/ns was kept fixed throughout all simulations. Supplementary Figure 11.1 shows the collected results.

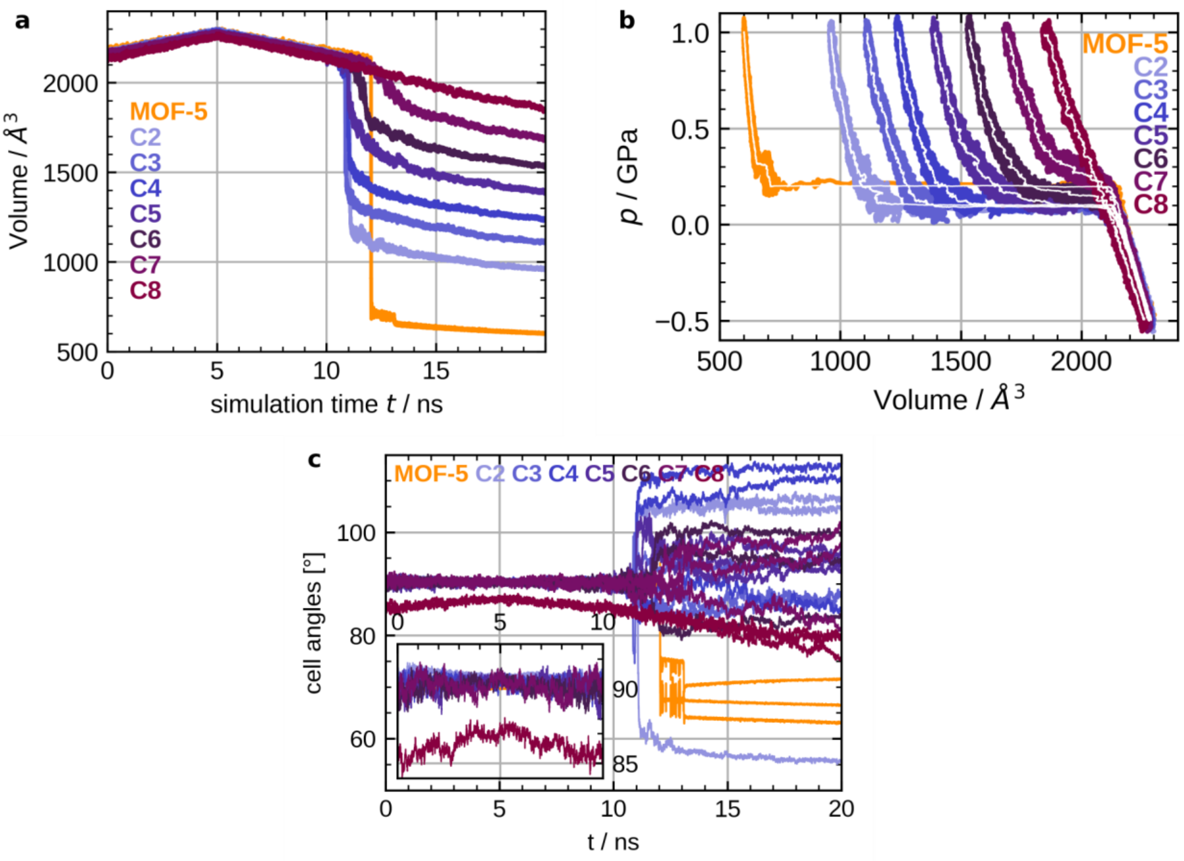

Supplementary Figure S11.1: Pressure ramp simulation results. **a**: Simulation time vs cell volume of the MOF-5-CX library, **b**: Cell volume as a function of pressure and **c**: Cell angles as a function of simulation time. The volume is plotted as a running average using a window of τ_V = 1 ps and the pressure as a running average with a τ_V = 10 ps window. White data points in **b** correspond to a window of τ = 100 ps for both V and p.

Whereas during the negative pressure the systems shows only a small isotropic cell volume change, the positive pressure ramp simulations trigger a phase transition for all systems but the C8 system, which already at the very beginning of the simulation transforms into its rhombohedral phase again. The onset of these phase transitions happens approximately at the same pressure of about 0.1 GPa for all systems, only the parent MOF-5 transforms later at a pressure of about 0.2 GPa. Prior to this transition, all systems are in its cubic form, as the cell parameters oscillate around 90° throughout the entire negative pressure ramp. Only the MOF-5-C8 cell parameters remain close to 85 °even at a negative pressure of –0.5 GPa.

In the final volumes we observe an approximately constant volume offset of about 150 Å^3 per additional carbon atom in the side chains in the volume the systems adapt at that pressure. Note also that the speed at which the non-linear compression – which we attribute as the phase transition region – is different for the different side chain lengths. In some cases, an intermediate region, where the cell angles have not yet fully converged, appears as e.g. for MOF-5, MOF-5-C2 and C3.

Temperature ramp NPT simulations

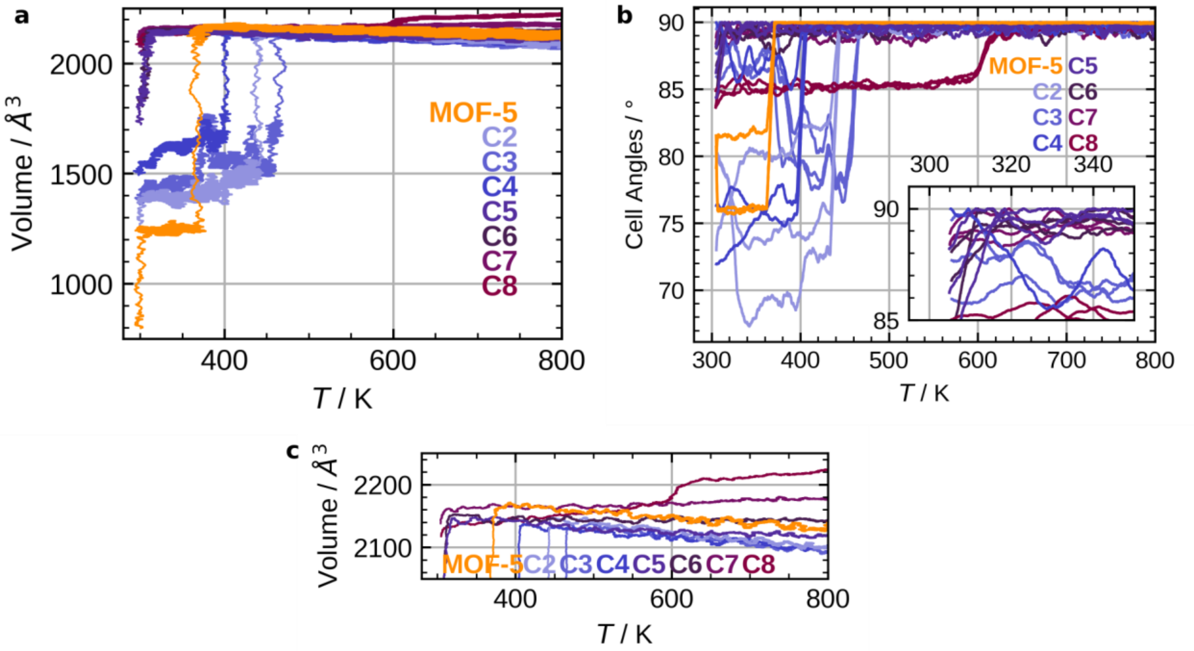

Supplementary Figure 11.2 a+c: Cell volume as a function of temperature during the temperature ramp *NP(σa=0)T* simulations. **b**: symmetrized cell angles as a function of temperature. The data is shown as centered running averages. The utilized window sizes are *V* = 10 ps, *T* = 1 ps (100 ps in **b**), cell angles = 100 ps.

In addition to the pressure ramp $NP(\sigma_a{=}0)T$ simulations, temperature ramp $NP(\sigma_a{=}0)T$ simulations at zero pressure were performed starting from the final frame of the pressure ramp $NP(\sigma_a{=}0)T$ simulations. Each system is equilibrated for 350 ps (100 ps NVT, 250 ps $NV(\sigma_a{=}0)T)$ and ramped from *T*=300 K to *T*=1000 K using a temperature ramp delta of 100 K/ns (totaling 7 ns production simulation). All systems open up to the cubic form at elevated temperatures as shown in Supplementary Figure 11.2. While for the smaller side chains we do not observe a trend in the transition temperature, the MOF-5-C8 system clearly is most resistant to the opening and thus starts its transformation at about 550 K, whereas all the other MOFs open up earlier. Some of the MOFs tend to reside for a short period in the intermediate volume, where half of the MOF in the simulation cell has already undergone the transition and the other is still in the closed phase. This is clearly visible for the parent MOF-5 and to some degree in the trajectories for MOF-5-C3 and MOF-5-C4. It further underlines that these states are considerably stable and are thus consistent with the experimental observation of the non-crystalline phase, where a large amount of these intermediate regions have to be present considering the proposed mechanism, structure and average cell volume.
An interesting trend in the thermal expansion coefficient is apparent in the volume trajectories. As MOF-5 is a material featuring negative thermal expansion,[25] we correctly observe its cell volume shrinking with increasing temperature. Interestingly, this attribute persists in our simulation up to the MOF-5-C6 system, whose thermal expansion coefficient is close to zero. Only the side chain lengths C7 and C8 have a positive coefficient. This is somewhat surprising since negative thermal expansion in MOF-5 is usually attributed to particular vibrational modes of the phenyl moiety which at elevated temperature get increasingly populated and increase in their amplitude.[26] Particular these vibrations thus seem not to be affected too much by the tethered side chains.

_p(V)_ Equation of State, inner Energy Δ_U(V)_ and free energy Δ_A(V)_

In order to compute the underlying *p(V)* equation of state (EoS), including the states not accessible in the $NP(\sigma_a{=}0)T$ simulations described above (namely volume data points in beneath the transition region, where *p(V)* has a positive slope), we make use of the methodology described in the literature[27], which is frequently used to compute the thermodynamic properties of flexible MOFs.[20–24] Its starting point is a trajectory with a structural transformation with an apparent volume change, as the pressure and temperature ramp simulations given above. By running simulations in the $NV(\sigma_a{=}0)T$ ensemble initiated from various snapshots at different volumes, the *p(V)* EoS can then be computed from the average of the instantaneous pressure of the simulation cell. In contrast to the *V(p)* trajectories obtained during the NPT simulations, this EoS can be interpolated and integrated to yield the underlying free energy profile (Δ*A(V)*) by means of thermodynamic integration.

$$\Delta A(V) = A(V) - A(V_{ref}) = -\int_{V_{ref}}^{V} p(V')dV'$$

Due to the constant volume simulations, in which only the cell shape is allowed to vary, the system is forced to remain also in instable regions, whereas in a non-constrained simulation the system would leave this state due to its instability.

Since the internal energy is directly accessible as the average total force field energy at each volume point, the total entropy change of the system can be calculated via the fundamental relation $A = U - TS$.

Initial structures were taken from the pressure ramp simulations by defining a volume grid and by selecting the structure from the trajectory, where the difference between the cell volume and the target cell volume is as small as possible. The residual difference was corrected by isotropically scaling the cell to the targeted volume. $NV(\sigma_a{=}0)T$ simulations are carried out based on both, the pressure ramp and the temperature ramp NPT simulations. Notably, we observed differences in the results comparing the $NV(\sigma_a{=}0)T$ *p(V)* results based on the temperature and the pressure ramp, as shown as an example in Supplementary Figure 11.3.

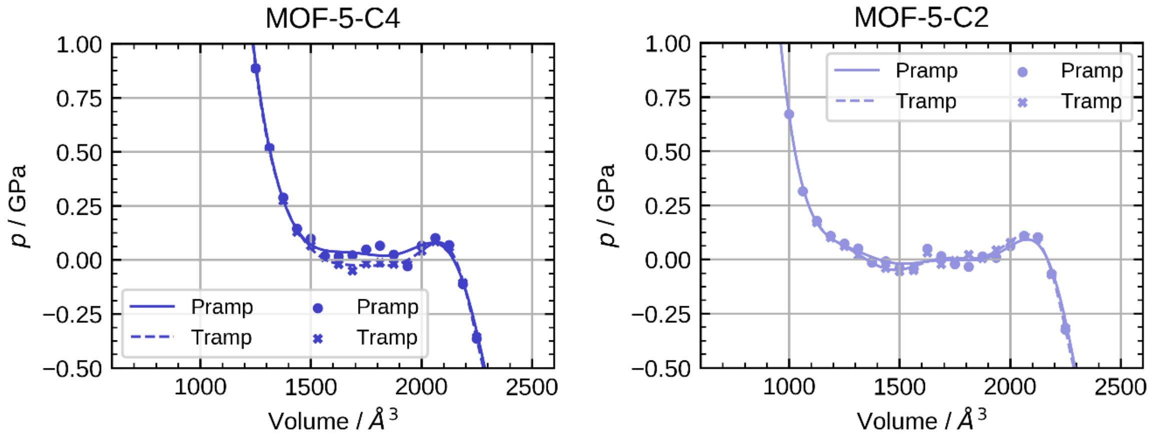

Supplementary Figure 11.3: Examples of *p(V)* derived from the pressure ramp (solid line, circles) and the temperature ramp (dashed line, crosses) for two structures, where *p(V)* differs (MOF-5-C4, left) and where they are nearly identical (MOF-5-C2, right).

In equilibrium, the results should be the same for each volume point no matter how the initial structural models were derived. Perhaps during the pressure ramps, the system was treated

too roughly, and the system did not have sufficient time to adapt its favored configuration. This is why, given that *p(V)* differs, the temperature ramp based simulations were used to compute the thermodynamic properties, as we assume they are the ones that are closer to mechanical equilibrium. We assume this because these structures are the ones that are aged more. Furthermore, it is usually easier to unfold than to fold a system.

Supplementary Figure 11.4 shows the *p(V)* EoS and the derived thermodynamic quantities ΔU, ΔA, $-T\Delta S$ as a function of cell volume and X in MOF-5-CX.

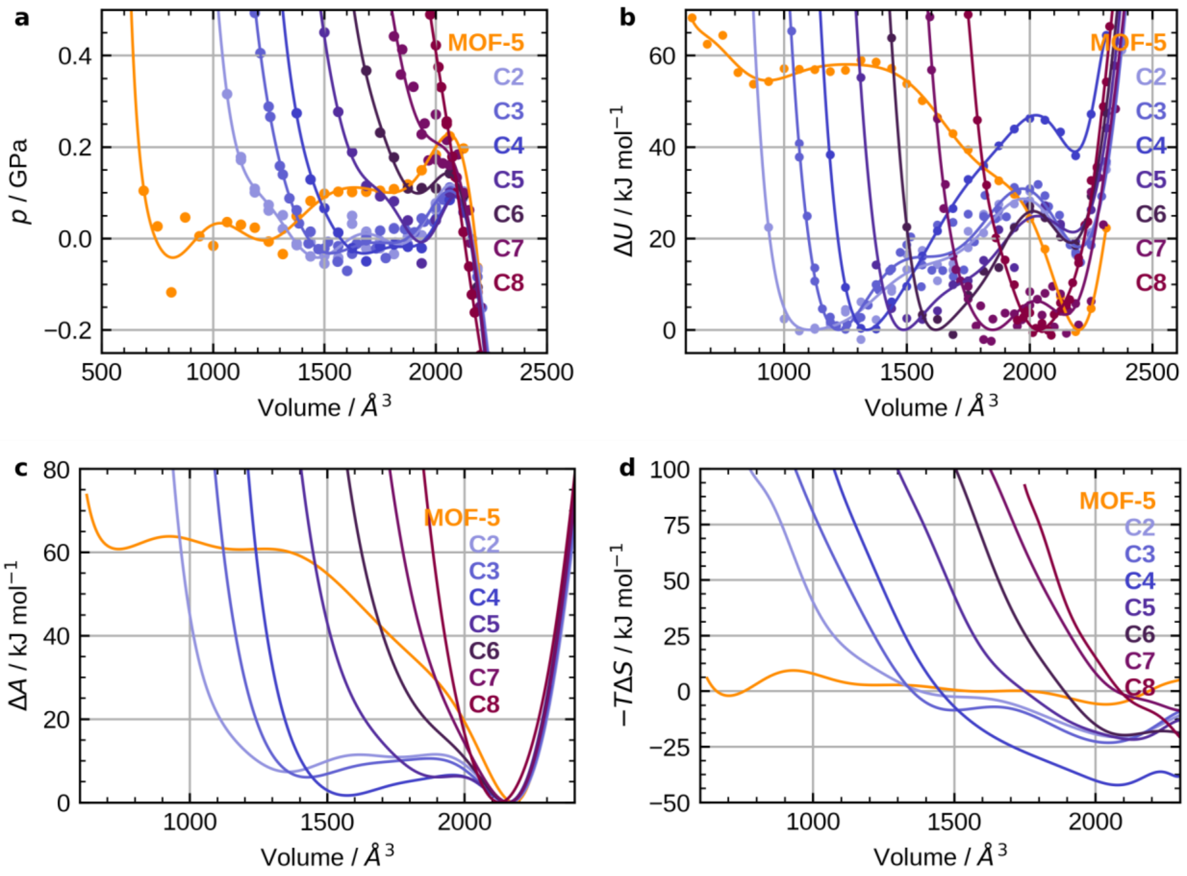

Supplementary Figure 11.4: Simulation results of the *NV(σₐ=0)T* simulations of the 2x2x2 supercell. **a**: The *p(V)* pressure points and the corresponding polynomial profile fits. **b**: The averaged total energies of the simulation, artificially shifted to zero origin for comparability. **c**: Relative free energy differences obtained by numerical integration of the fits of *p(V)* shown in in **a**. **d**: Contribution of the entropy ($-T\Delta S$ term) to the free energy as a function of cell volume. This contribution is calculated from the fit of the internal energies shown in **b** and an interpolation of the free energy shown in **c**.

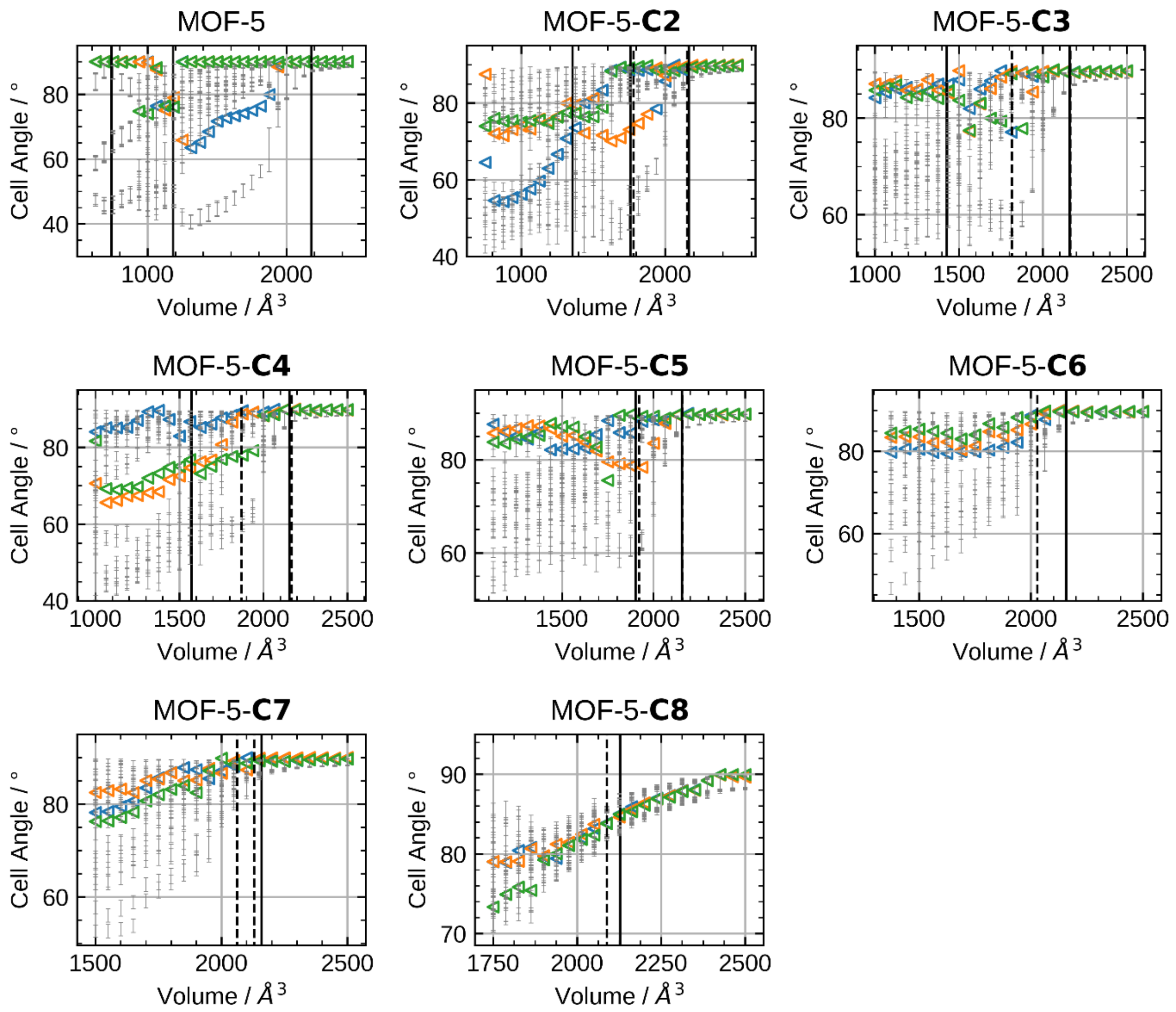

Supplementary Figure 11.5 The average 2x2x2 cell angles along the *NV(σ_a=0)T* trajectories are shown as the blue(α), orange(β), green(γ) symbols: Shown in grey are the results of the cell volume analysis (i.e. the for the eight small cells in the 2x2x2 supercell). The error bar of each of the 24 cell angle data points per volume (3 angles for each of the eight Zn_4O clusters) denotes the standard deviation of the respective cell angle trajectory. Minima positions in the free energy minima of the respective structure are indicated as the solid black lines. Experimentally determined Volumes are indicated as the dashed black lines.

The pressure profile shown in Supplementary Figure 11.4a indeed shows for MOF-5-C2 to MOF-5-C5 a negative pressure region, indicative for a (local) free energy minimum in this region. However, none of these are the global free energy minimum and hence describe metastable states of our simulated 2x2x2 models. The barriers connecting these states, however, are quite small and hence the free energy function is shallow in between the two local minima up to the point that another minimum is observed for MOF-5-C2. The driving force that stabilizes the distorted form in all cases is the inner energy. For all structures except for the parent MOF-5 material, the internal energy minimum corresponds to a contracted distorted form. It is the entropy that penalizes the contracted forms to be only metastable states, at least in the small 2x2x2 supercell simulations.

Cell volume analysis

In order to assess how each individual cluster reacts during the phase transformation, we calculated for each cluster the position of the seven neighboring clusters in positive direction and approximate the volume of a single cell corresponding to one formular unit (or one cluster), as the convex hull of the eight points. Additionally, the cell angles are approximated from the vectors that connect the current Zn_4O cluster with the adjacent ones in $\langle 100 \rangle$, $\langle 010 \rangle$ and $\langle 001 \rangle$ direction (of the cubic form). Note that the sum of all these volumes does not fit quantitatively to the 2x2x2 supercell volume of the simulation box. This is due to a small volume region at the borders of two of these cells which is counted twice due to the use of the convex hull to calculate the cell volumes. We estimate this error to be about 1-2 % for the cubic form and up to 10 % for the distorted forms, where the double-counting is more pronounced. Since this is a systematic error, it does not affect the comparability of the results of this analysis.

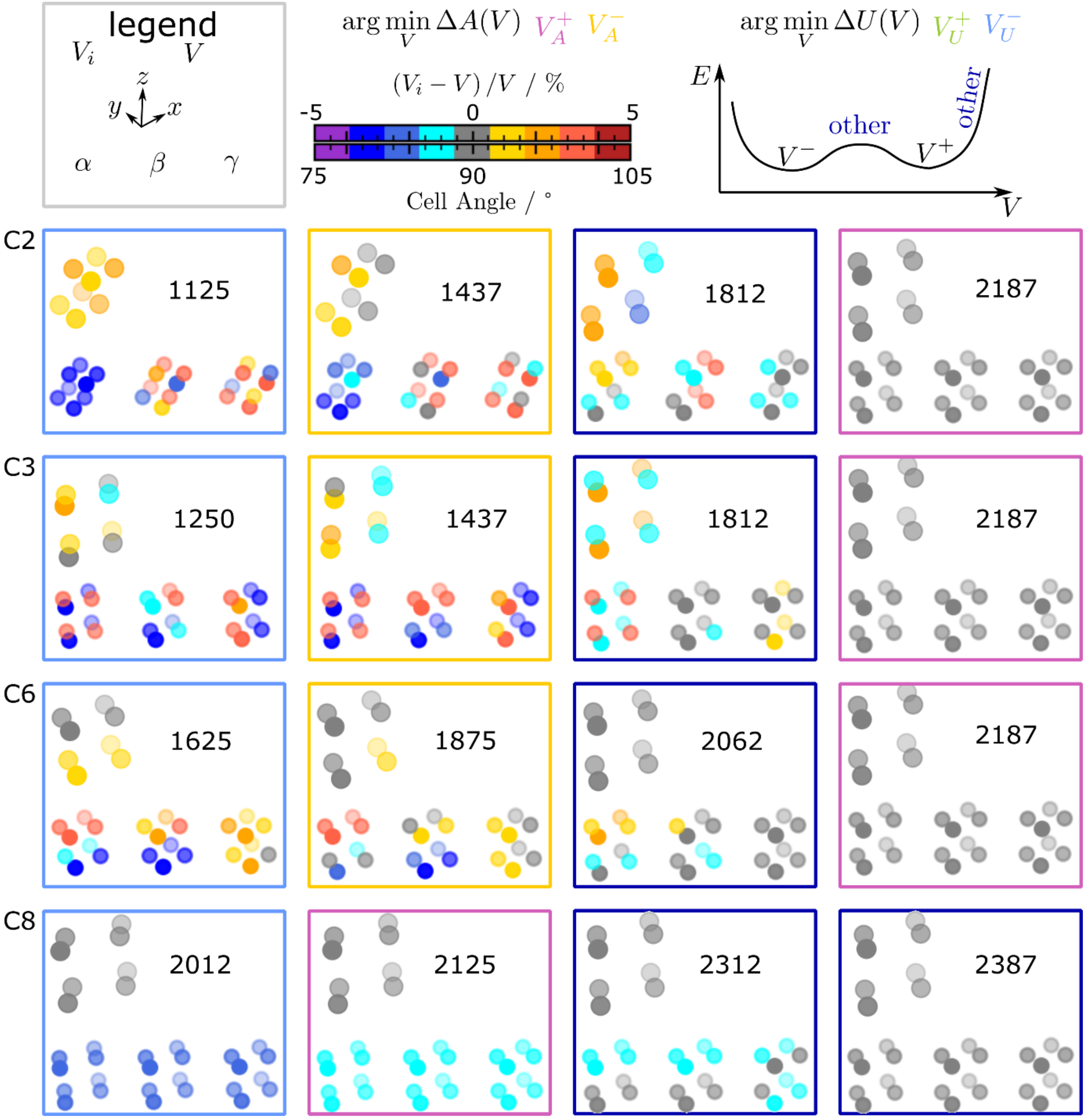

Supplementary Figure 11.6 Results of the cell volume analysis for selected structures and volumes. The positions of the points are calculated from the centre of the eight adjacent

Zn_4O units. Each box contains the differences in volume (top left) comparing the individual cell volumes with the average simulation box volume of the respective $NV(\sigma_a=0)T$ simulation, where blueish colours denote a negative deviation and the reddish colours denote a positive deviation. The respective volume is given at the top right. The bottom images denote the individual cell angles (left: ∢ xy, centre: ∢ xz, right: ∢ yz), where the grey colour corresponds to an angle close to 90°, the blueish colours denote an angle smaller 90° and the reddish colours denote angles larger than 90°.The colours of the boxes correspond to the energetics of the respective system type MOF-5-CX at the given volume. Magenta and yellow correspond to minima in the free energy, with magenta being the large volume minimum (V^+) and yellow the small volume minimum (V^-). Volumes with no apparent energy minimum, which, are interesting for the discussion herein are marked dark blue. Light blue boxes denote the largest cavity volume, where the internal energy features a minimum (V^-_U). The centre of the pore (of the respective cell) calculated from the average of the oxygen positions is used as representative position (the scatter coordinates).

Supplementary Figure 11.6 shows this analysis for a selection of structures and cell volumes. Interestingly we observe a concerted rhombohedral distortion only for MOF-5-**C8**, whereas – even in these small simulation boxes – the distortion directions for the other systems are different for the eight individual cavities of each simulation cell. These distortion directions are, however, not entirely random, but we can observe patterns in them: In MOF-5-**C3** V=1437 Å^3 for example, layers of distortions in different directions appear and each individual angle forms a layer in a different spatial dimension (i.e. $\sphericalangle\vec{x}\vec{y}$ in the x-y plane, $\sphericalangle\vec{x}\vec{z}$ in the x-z plane and $\sphericalangle\vec{y}\vec{z}$ in the y-z plane). This results also in an inhomogeneous distribution of the individual cell volumes. This may be a consequence of the artificially enforced symmetry due to the small simulation cells.

The inhomogeneous volume distribution arises from the different cell angles ∢xy, ∢xz and ∢yz that change their angle in different directions. As a trend these three angles form layers, where in one layer the tilt is opposing to the other one. This is not always the case, but we identify it as a general trend.

Simulated PXRDs

We computed the full set of PXRD patterns for each *NVT* trajectory given 200 sample structures from each trajectory.

Below the averaged PXRD patterns are shown for a selection of volume points. They reveal for all structures except of MOF-5-C8 the presence of a cubic structure (**a**) at simulation conditions, as their PXRD patterns at V^+ compare to the pattern of MOF-5. The PXRDs of MOF-5-CX (2 ≤ X ≤ 5) in the local free energy minimum V^- (the metastable contracted state) have entirely lost their main peak at Q = 0.5 Å^{-1} (**b**) , whereas at experimentally determined volumes corresponding to the contracted phase (**d**), this main peak persists in diminished intensity. In both cases, however the number of small peaks increases resulting in a broad distribution of reflections compared to the PXRD patterns of the cubic phases. Intense peaks occur at about Q = 0.6 ± 0.1 Å^{-1}, which is just the position of the FSP in the experimental diffraction pattern of the dry-MOF-C2 to -C6 materials.

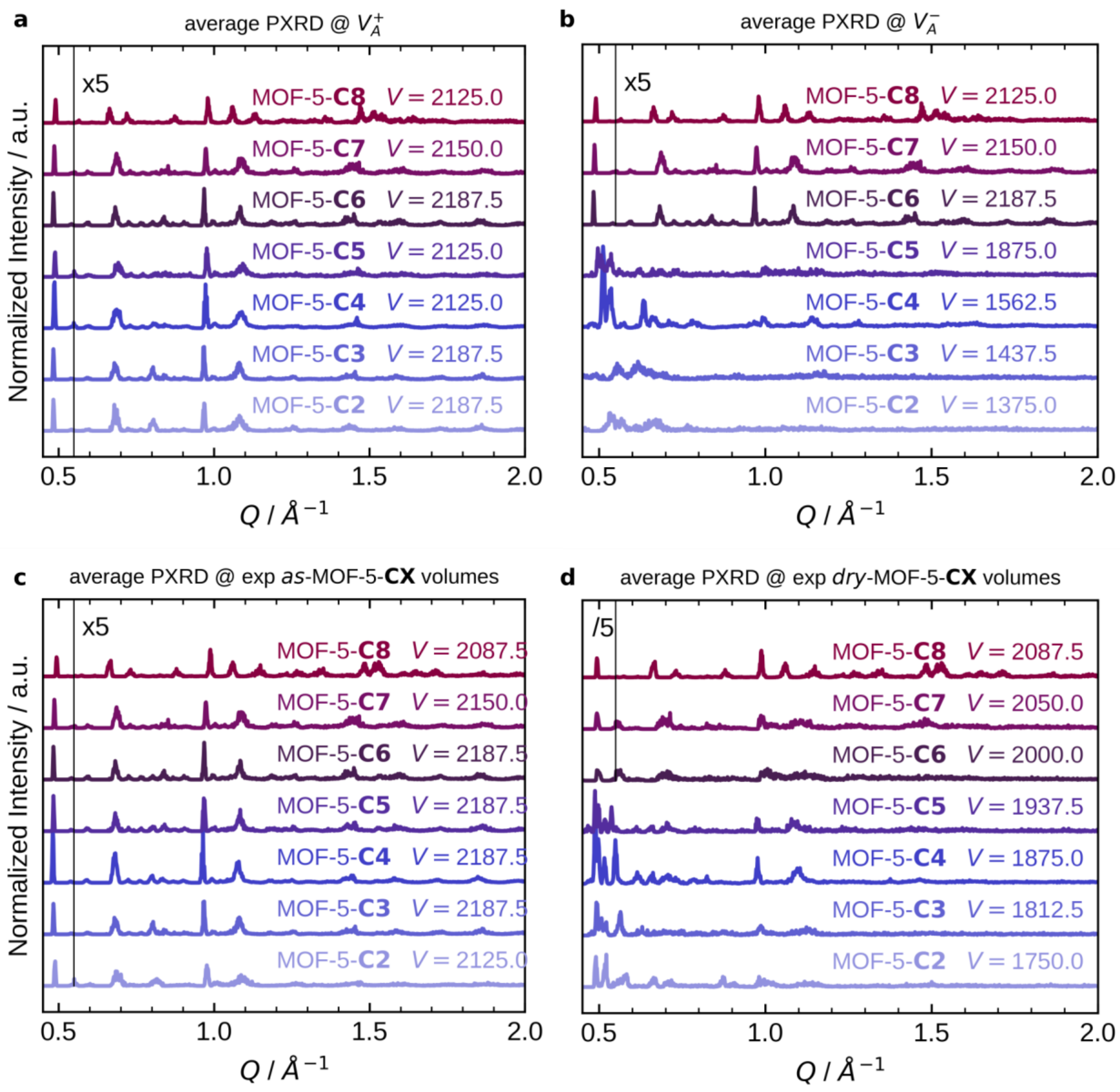

Supplementary Figure 11.7: Average PXRD patterns along trajectories of the *NV(*σ_a*=0)T* simulations shown for the different interesting volume regions. **a** corresponds to the large volume forms as predicted from the free energy minimum V^+_A and **b** the small volume free energy minima V^-_A. **c** and **d** contain the PXRDs at the experimentally determined large volumes (**c**) and small volumes (**d**).

Simulated XPDFs

We computed the full set of XPDFs for each *NVT* trajectory given 200 sample structures from each trajectory. The XPDFs at different volumes are shown in Supplementary Figure 11.8. All the main peaks comparing to experiment are well reproduced in the cubic (C2-C7) or rhombohedrally distorted (C8) XPDFs. Interestingly, even these small 2x2x2 supercells show signs of the loss of long-range order of the contracted forms: At the experimentally determined distorted cell volumes, peaks beyond 12 Å lose some of their intensity for MOF-5-CX (X ≠ 8). XPDFs of the shorter side-chains (C2 to C5) at the simulated local free energy minimum V^-_A even show diminished peak intensities starting at 5 Å. Hence, we observe the qualitatively correct tendency regarding the loss of long-range order in these small cell simulations, but with significant quantitative discrepancies, emphasizing on the need to go

beyond these small simulation cells and the associated enforced symmetry due to periodic boundary conditions.

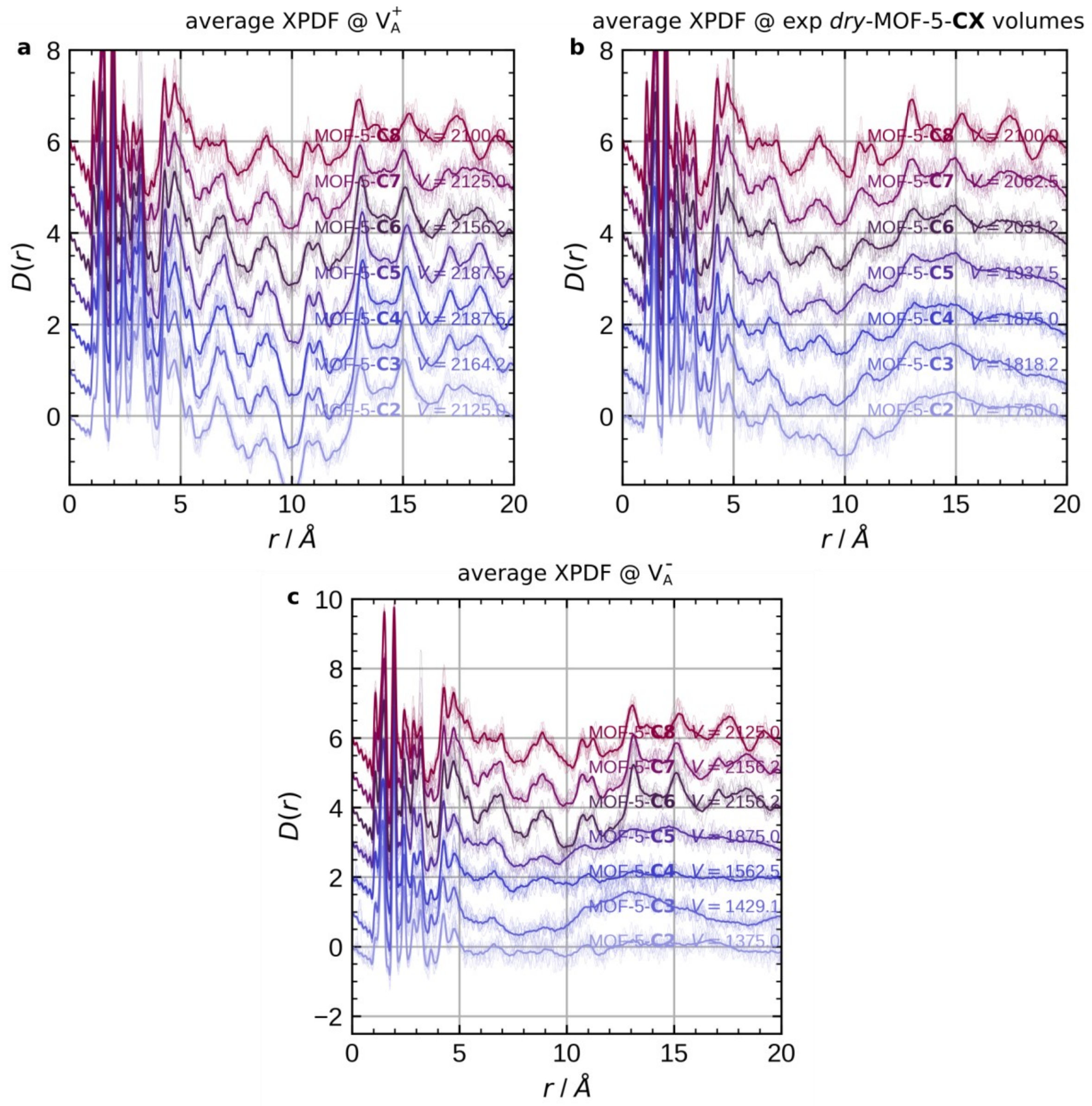

Supplementary Figure 11.8: Simulated XPDFs of the cubic forms (**a**), at the volumes corresponding to the experimentally determined distorted average cell volumes (**b**) and at the smallest volume free energy minima V^-_A.

8x8x8 Supercell simulations

The large simulation boxes for MOF-5-C3, MOF-5-C6 and MOF-5-C8 are based on the optimized cubic initial structures. The 8x8x8 supercells with a simulation box dimension of about 10.4 nm contain 512 Zn_4O clusters and a total of 57856 (C3), 85504 (C6) and 103936 (C8) atoms.

The cubic forms of MOF-5-C3 and MOF-5-C6 were pressure ramped to 0.25 GPa and 0.5 GPa, respectively, starting in the cubic phase and MOF-5-C8 was temperature ramped from 300 K to 1000 K starting in the rhombohedral phase.

The stimulus was then gradually released, and the systems were propagated for another 0.5 ns to observe the structure back at ambient conditions. The resulting atomistic representation and cell volume analyses are given in Supplementary Figure 11.9 for MOF-5-C3 and in Supplementary Figure 11.10 for MOF-5-C6, respectively.

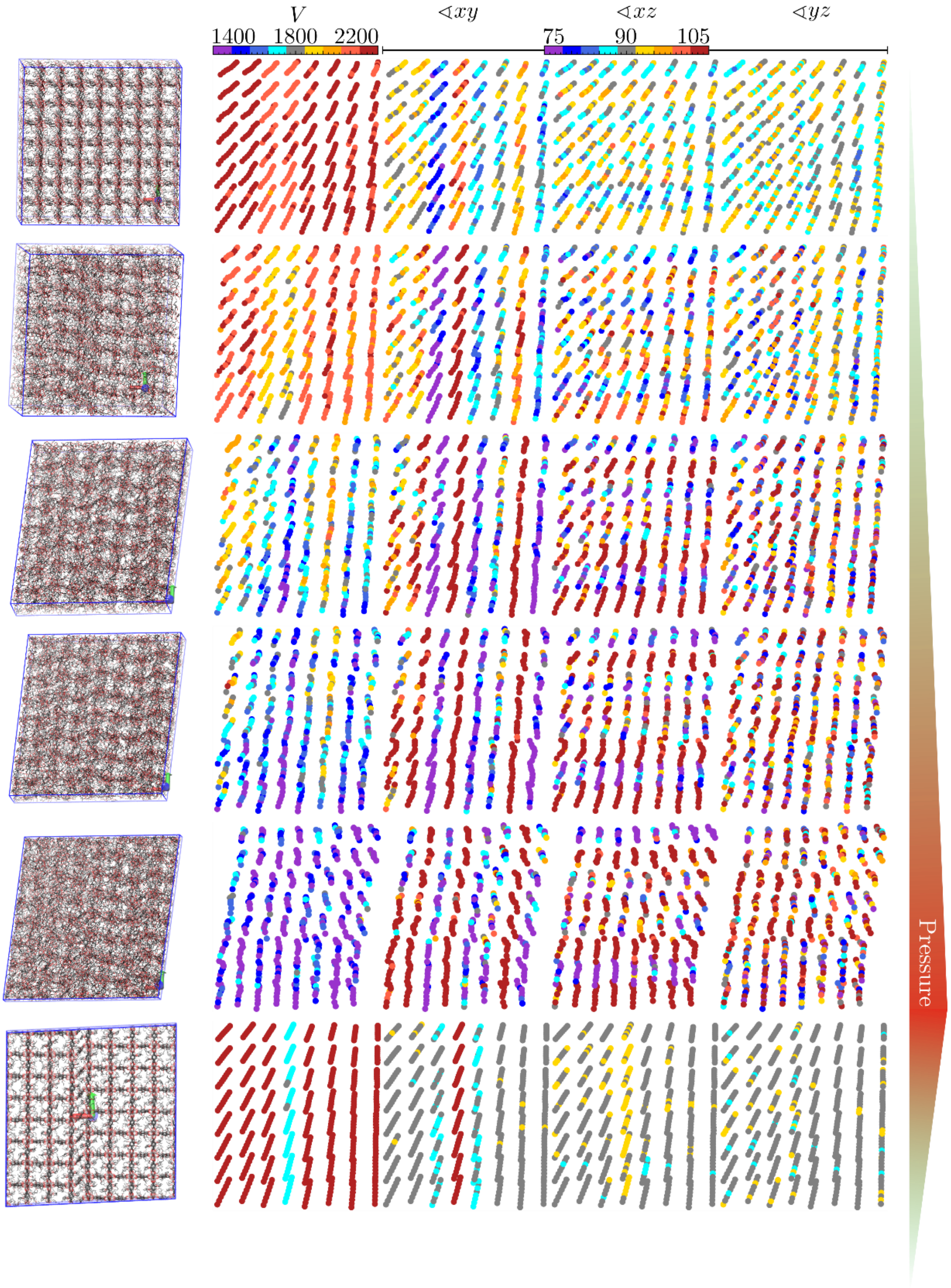

Supplementary Figure 11.9: Trajectory of the pressure ramp MOF-5-C3 8x8x8 supercell simulation. From left to right: atomistic representation, single cell volume and the latter three columns display the differences from the individual single cell angles (∢xy, ∢xz and ∢yz) from 90°.

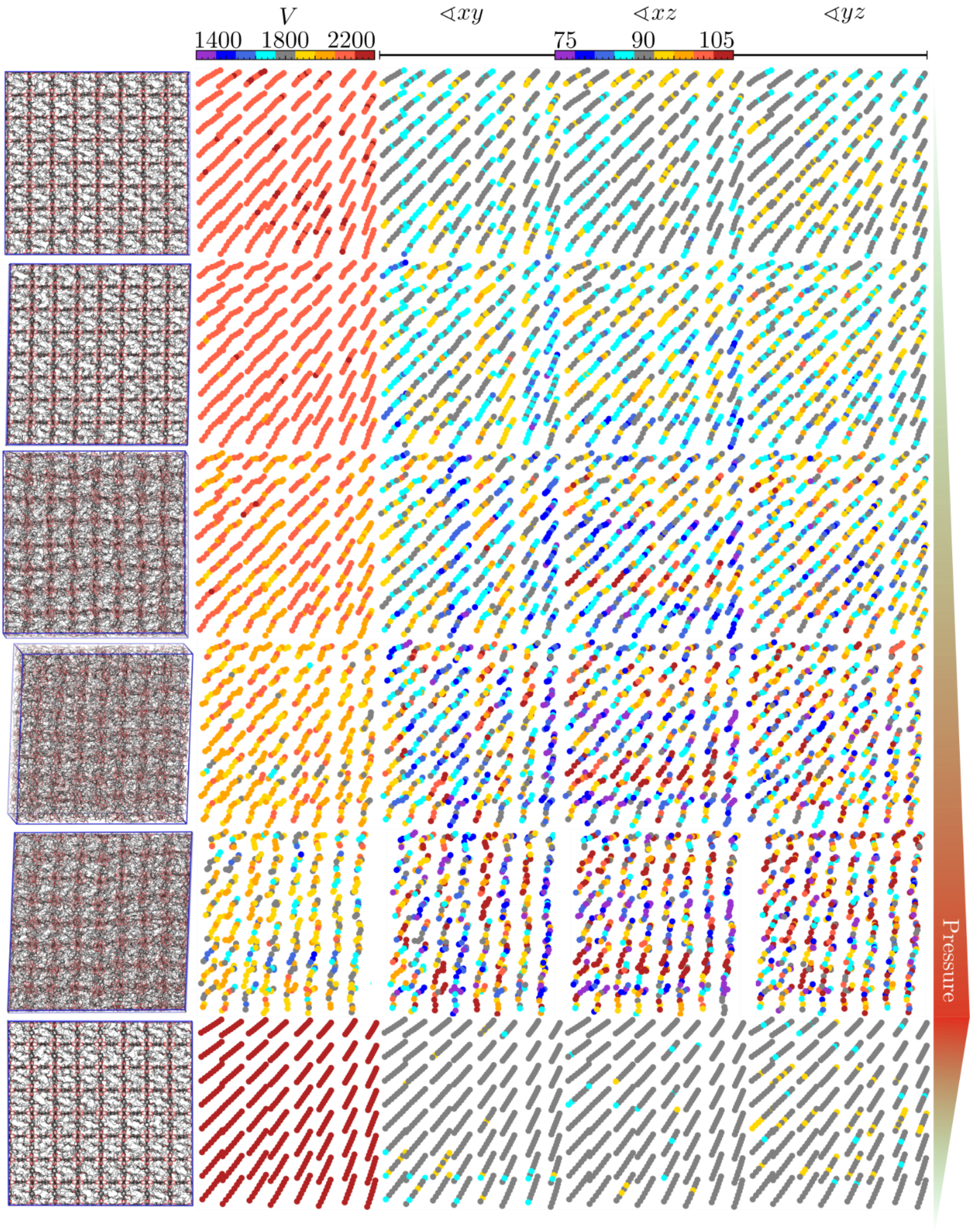

Supplementary Figure 11.10: Trajectory of the pressure ramp MOF-5-C6 8x8x8 supercell simulation. From left to right: atomistic representation, single cell volume and the latter three columns display the differences from the individual single cell angles (∢xy, ∢xz and ∢yz) from 90°.

PXRD and XPDF patterns

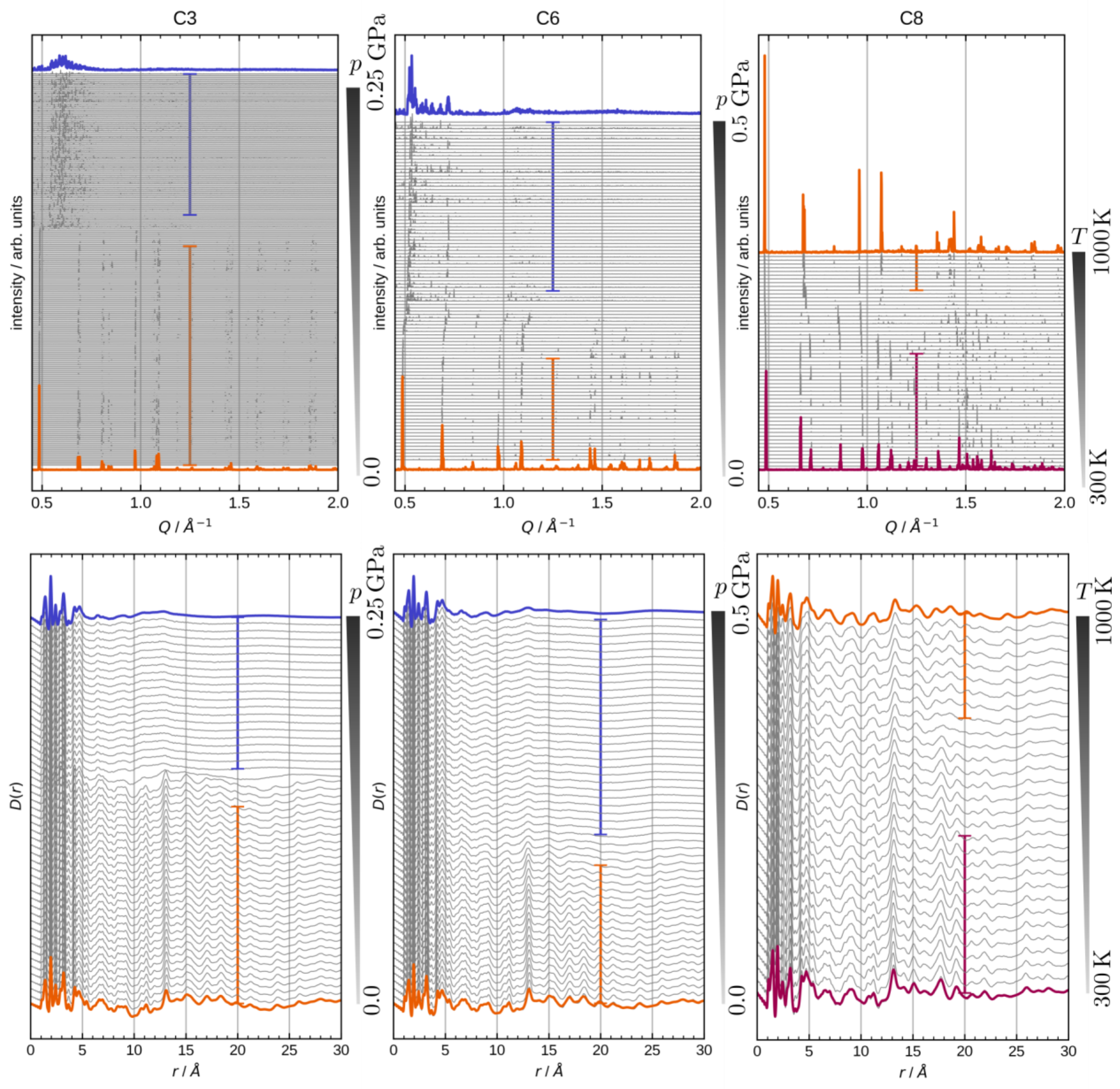

Supplementary Figure 11.11: Simulated PXRD patterns (top) and XPDFs (bottom) of the 8x8x8 supercell simulations, where the structural transformation was enforced with pressure (C3 (left) and C6 (center)) and with temperature (C8 (right)). The respective windows to obtain the average patterns are marked as isochromatic range. The averages are plotted in Figure 4 **g** and **h** of the main text.

Average O_{eth}-C_{Methyl} distances.

In Supplementary figure 11.12 the distance histograms of the distance between the alkoxy oxygen atom and the terminal carbon atom of the respective alkoxy side chain are shown both for the cubic and the distorted phase. Notably, the mean distance between these atoms increases from the cubic to the distorted phase. The longer the mean distance, the more elongated is the side chain. This leads to a stronger population of the energetic optimum staggered configuration. These results underline the need for the side chains to reorient during the cubic-to-non-crystalline phase transition.

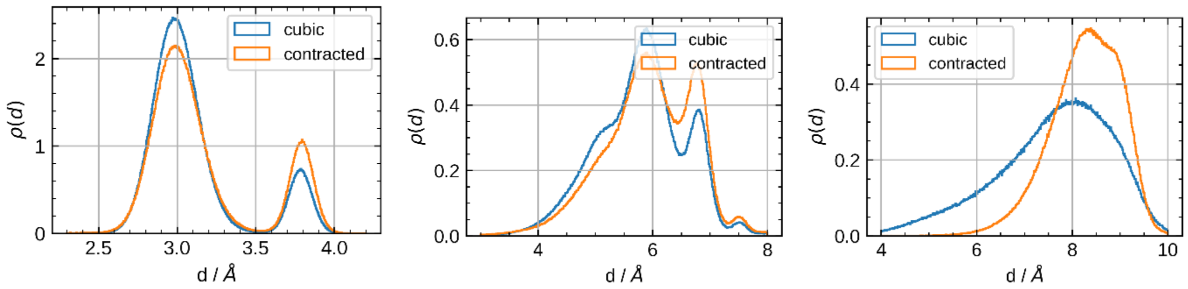

Supplementary Figure 11.12: $C_{R\text{-}CH3}$-O_{eth} distance histograms of C3 (left), C6 (center) and C8 (right), each averaged along the trajectory where the phase could be identified from Supplementary Figure 11.8.

Influence of the tethering of the side chains to the MOF-5 backbone: MOF-5-C8

Over the course of this investigation, we considered the option that the structural deformation present in the MOF-5-CX systems could also be triggered by adsorption, rather than the tethering of the DEDs to the backbone of the framework. In order to assess this, we artificially cut the C_{alkyl}-O_{eth} bond and deleted all bond, angle and torsion terms that include this bond of MOF-5-C8 during an $NP(\sigma_a=0)T$ simulation at ambient pressure starting in the rhombohedrally distorted phase.

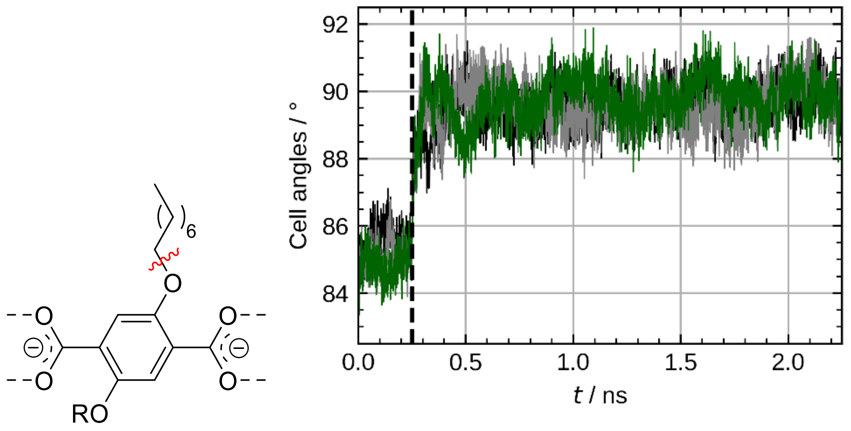

Supplementary Figure 11.13: left: the bond cut at *t*=250 ps in the *NPT* simulation is indicated as the red curly line. Right: Cell angles as a function of simulation time. The timeframe where the bonds were cut is indicated as the dashed line.

The system directly and within only several picoseconds responds with a structural transition towards a cubic simulation cell once the bonds are cut. Hence the tethering of the side chains mediating the stress onto the framework by means of dispersive interactions is the root cause for the rhombohedral distortion.

Strain energetics of the parent MOF-5 material

In order to estimate the deformation energy penalty of a single Zn_4O unit during a rhombohedral distortion, cell angles of the parent MOF-5 material were systematically varied from 90° to 100°, each followed by an optimization of the atom positions keeping the cell

constant. The resulting energy profile per formula unit $Zn_4O(bdc)_3$ is shown in Supplementary Figure 11.14.

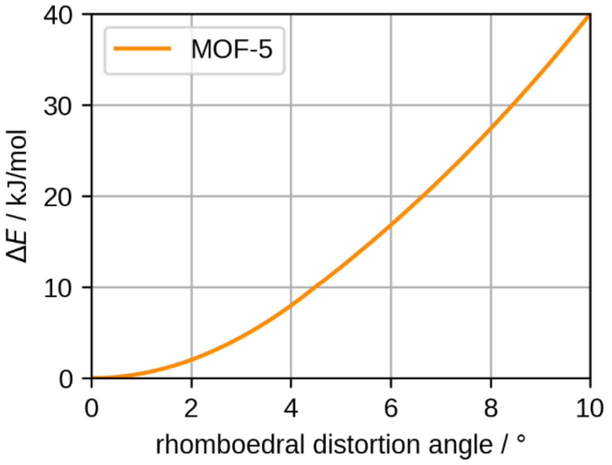

Supplementary Figure 11.14: Energy scan of the parent MOF-5 material (simulation cell containing 2x2x2 cavities) as a function of rhombohedral distortion angle. A rhombohedral distortion angle of 4 ° corresponds to cell angles of 94°. The energy is given per formula unit $Zn_4O(bdc)_3$.

Experimentally, the rhombohedral phases of dry-MOF-5-C7 and dry-MOF-5-C8 are distorted by about 5°, meaning, in these cases, the DEDs have to account for at least 12.5 kJ/mol per formula unit, i.e. per six side-chains to overcome this strain. The shorter side-chain MOFs cannot directly be compared since there are no experimental cell angles available. However, the global free energy minimum volumes in the small cell simulations feature rhombohedral distortion angles between 5° and up to more than 10° (MOF-5-C3), yet the total internal energy of this dense form is substantially smaller compared to the open cubic form (see Figure 4b or Supplementary Figure 11.4), suggesting an even larger effect of the DEDs on the order of 30-50 kJ/mol per formula unit.

Supplementary Methods 12 - Experimental data on MOF-5-CX with X = 9, 10

If not otherwise mentioned the experimental data listed herein were collected under the same conditions as stated under Methods.

Supplementary Methods 12.1 – Synthesis of H_2(C9-bdc) and H_2(C10-bdc)

2,5-Nonyloxy-1,4-benzenedicarboxylic acid and 2,5-decyloxy-1,4-benzenedicarboxylic acid were prepared using the same procedure as stated under Supplementary Methods 1.1 by replacing the alkyl halide with 1-Bromooctanefor H_2(C9-bdc) and 1-Bromodecane for H_2(C10-bdc).

2,5-Nonyloxy-1,4-benzenedicarboxylic acid (H_2(C9-bdc), ^{1}H NMR (400 MHz, DMSO-d_6): δ 12.89 (s, 2 H, COOH), 7.26 (s, 2 H, Ar–H), 3.97 (t, J = 6.4 Hz, 4 H, OCH_2), 1.72-1.62 (m, 4 H, CH_2), 1.45-1.36 (m, 4 H, CH_2), 1.34-1.20 (m, 20 H, CH_2), 0.86 (t, J = 6.9 Hz, 6 H, CH_3) ppm; ^{13}C NMR (126 MHz, DMSO-d_6): δ 166.83, 150.43, 125.49, 115.53, 69.13, 31.29, 28.98, 28.71, 28.64, 25.32, 22.10, 13.9 ppm.

2,5-Decyloxy-1,4-benzenedicarboxylic acid (H_2(C10-bdc), ^{1}H NMR (500 MHz, DMSO-d_6): δ 12.84 (s, 2 H, COOH), 7.24 (s, 2 H, Ar–H), 3.96 (t, J = 6.4 Hz, 4 H, OCH_2), 1.69-1.62 (m, 4 H, CH_2), 1.43-1.36 (m, 4 H, CH_2), 1.32-1.19 (m, 24 H, CH_2), 0.84 (t, J = 6.9 Hz, 6 H, CH_3) ppm; ^{13}C NMR (101 MHz, DMSO-d_6): δ 167.36, 150.87, 125.98, 115.93, 69.54, 31.78, 29.51, 29.42, 29.20, 29.18, 29.16, 25.79, 22.59, 14.45 ppm.

The corresponding materials MOF-5-C9 and MOF-5-C10 were synthesized using the linkers H_2(C9-bdc) or H_2(C10-bdc) and the methods described under Supplementary Methods 1.4.

Supplementary Methods 12.2 – PXRD

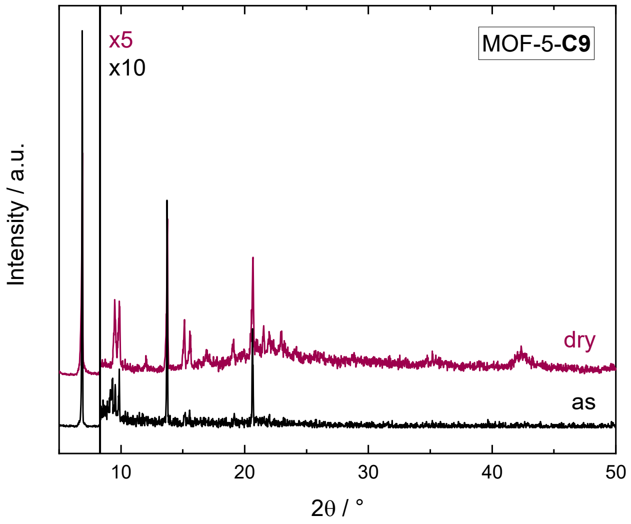

Supplementary Figure 12.1: PXRD patterns of as-MOF-5-C9 and dry-MOF-5-C9. The data quality of the diffraction pattern of as-MOF-5-C9 is limited, however, the compound appears to be rhombohedral. dry-MOF-5-C9 clearly is rhombohedral (see profile fit in Supplementary Figure 12.3). The rhombohedral distortion of dry-MOF-5-C9 is a bit smaller compared to dry-MOF-5-C8 and dry-MOF-5-C7, as expected due to the larger bulk of the C9 side chains.

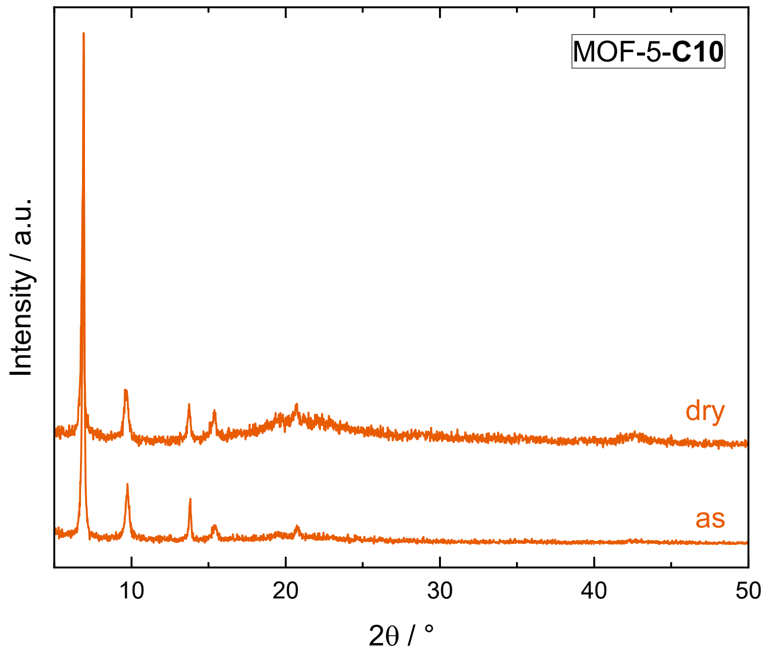

Supplementary Figure 12.2: PXRD patterns of as-MOF-5-C10 and dry-MOF-5-C10. MOF-5-C10 is cubic in the as synthesized state and remains cubic after guest removal (see profile fit in Supplementary Figure 12.4). We thus conclude that the C10 side chains fully occupy the pores in the cubic form, so that a rhombohedral distortion is not possible in the guest-free state.

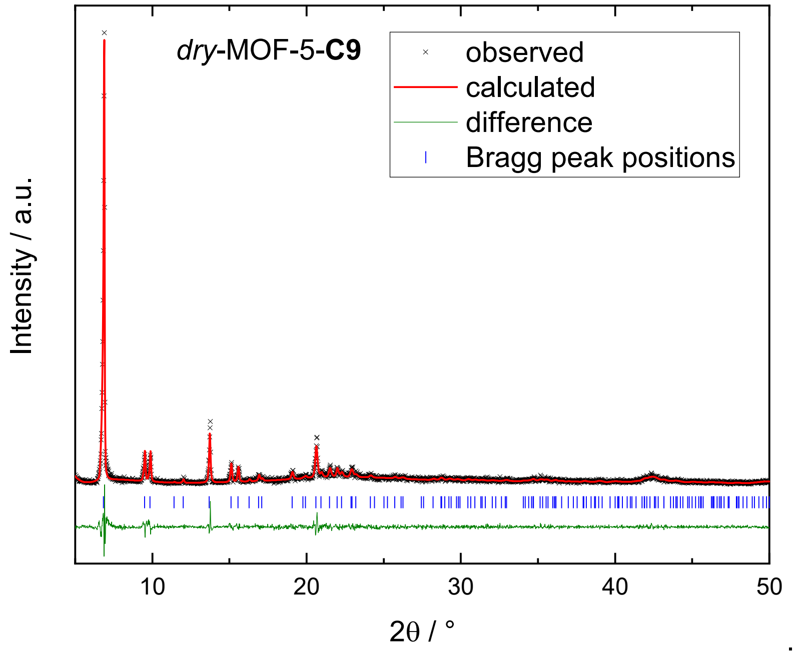

Supplementary Figure 12.3: PXRD pattern with profile fits (Pawley method) of dry-MOF-5-C9 (see Supplementary Table 12.1 for crystallographic data).

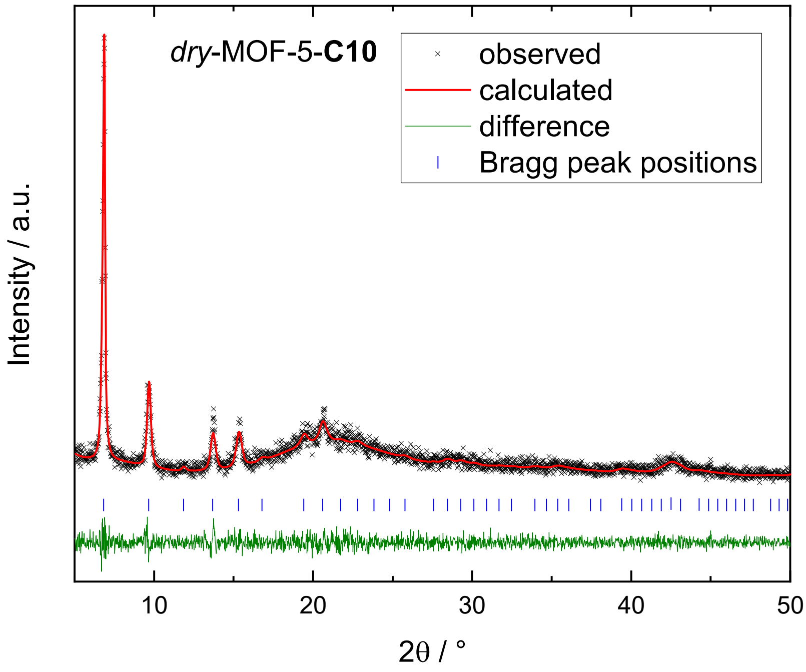

Supplementary Figure 12.4: PXRD pattern with profile fits (Pawley method) of dry-MOF-5-C10 (see Supplementary Table 12.1 for crystallographic data).

Supplementary Table 12.1: Unit cell parameters *a*, *b*, *c* and cell volume *V* determined by profile fitting (Pawley method) of the PXRD patterns and the corresponding R_{wp}, R_{exp} and χ^2 values for the dry-MOF-5-C9 and dry-MOF-5-C10.

Compound	dry-MOF-5-C9		dry-MOF-5-C10
crystal system	trigonal	rhombohedral	cubic
space group	$R\bar{3}$		$Pm\bar{3}m$
a / Å	17.957(4)	12.944(3)	12.931(10)
b / Å	17.957(4)	12.944(3)	12.931(10)
c / Å	23.252(7)	12.944(3)	12.931(10)
α / °	90	87.84(2)	90
β / °	90	87.84(2)	90
γ / °	120	87.84(2)	90
V / Å^3	6493(3)	2164(2)	2162(5)
R_{wp}	19.31		17.25
R_{exp}	15.85		16.41
χ^2	1.48		1.11

Supplementary Methods 12.3 - IR spectroscopy

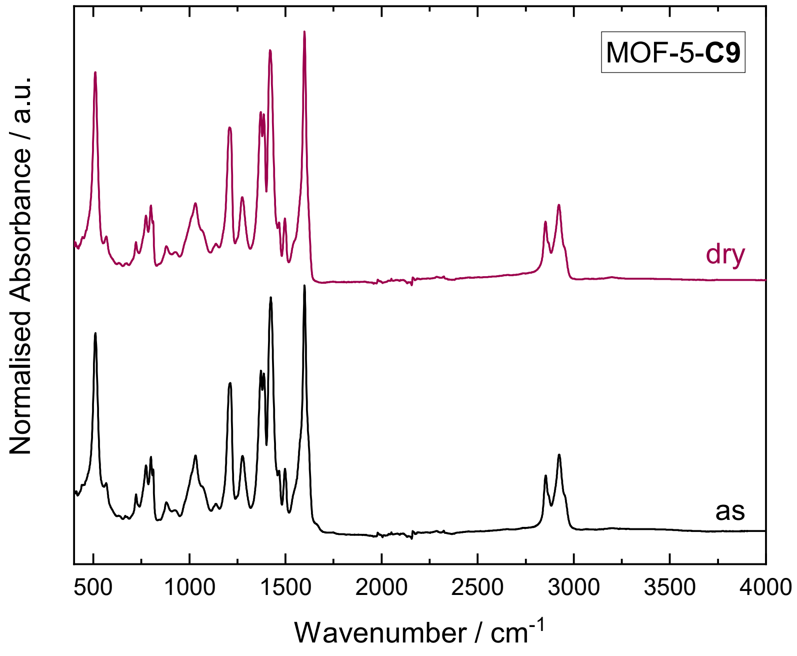

Supplementary Figure 12.5: FT-IR spectra of as-MOF-5-C9 and dry-MOF-5-C9.

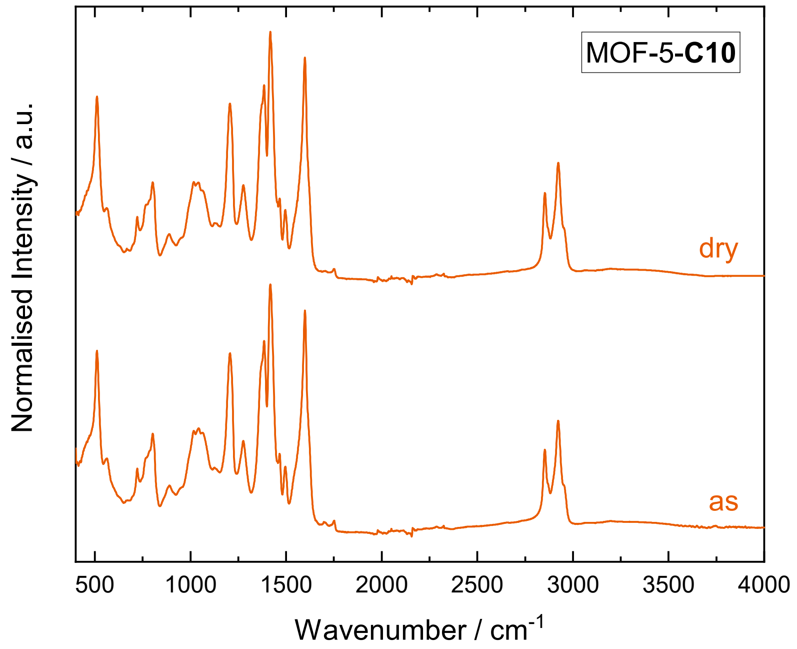

Supplementary Figure 12.5: FT-IR spectra of as-MOF-5-C10 and dry-MOF-5-C10.

Supplementary Methods 12.4 - Thermal analysis

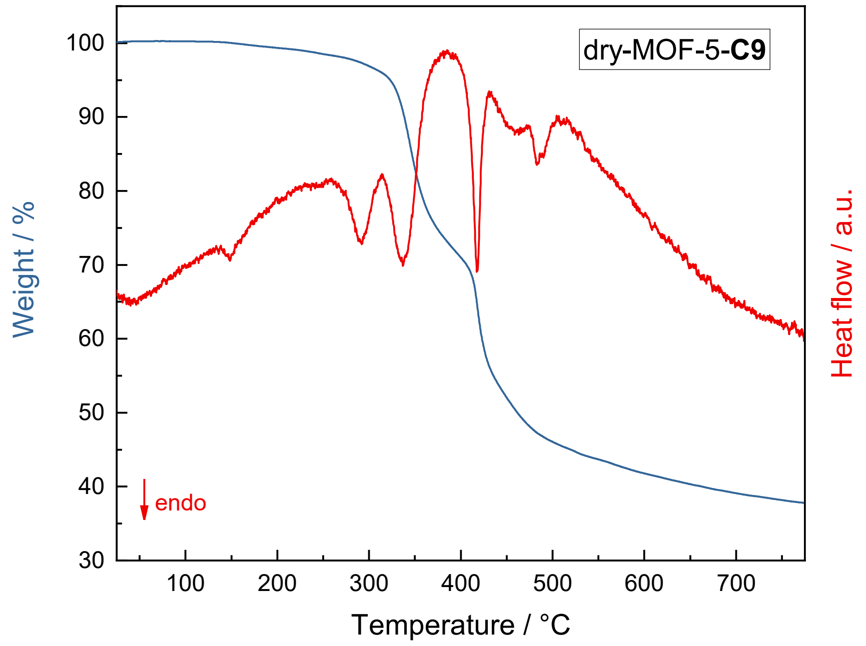

Supplementary Figure 12.6: TG-DSC data of dry-MOF-5-C9. The endothermic signal at approx. 160 °C is ascribed to the phase transition from the rhombohedral to the cubic phase. This is in excellent agreement with variable temperature PXRD data (see Supplementary Figure 12.8).

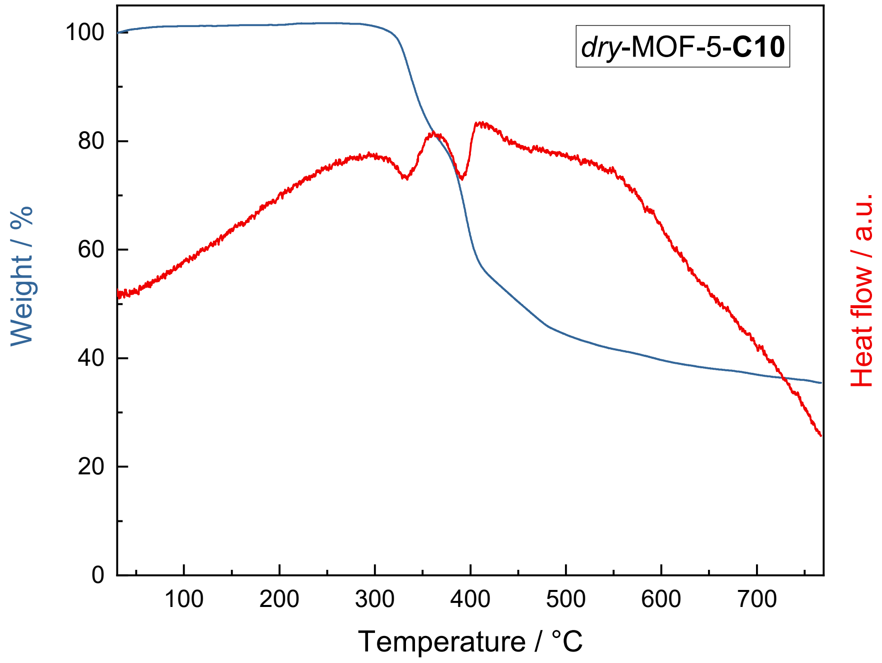

Supplementary Figure 12.7: TG-DSC data of dry-MOF-5-C10.

Supplementary Methods 12.5 - Variable temperature PXRD

Collection of PXRD data as a function of temperature was conducted at beamline BL9 at DELTA (Dortmunder Elektronenspeicherring-Anlage, Dortmund, Germany) with a monochromatic X-ray beam (λ = 0.620 Å) using a MAR345 image plate detector. The samples were finely ground, filled into 1.0 mm diameter quartz capillaries and placed on an Anton Paar DHS1100 hot stage and heated under a polyether ether ketone (PEEK) dome. The temperature calibration of the hot stage was performed by reference XRPD measurements of α-quartz.

A series of additional broad reflections from about 6.9° 2θ originate from the dome's material. Thus, only the reflections between 2° and 6.5° 2θ will be discussed here. Due to the limitations of the setup and the large width of the capillary, the reflections of dry-MOF-5-C9 are rather broad (See Supplementary Figure 12.8). Nevertheless, the rhombehedral crystal structure is clearly visible from the double reflections around 3.9° and 6.2° 2θ. Between 150 °C and 175 °C both double peaks merge into a single peak, which is a clear signature for the rhombohedral-to-cubic phase transition. The transition temperature is in excellent agreement with the TG-DSC data shown in Supplementary Figure 12.6.

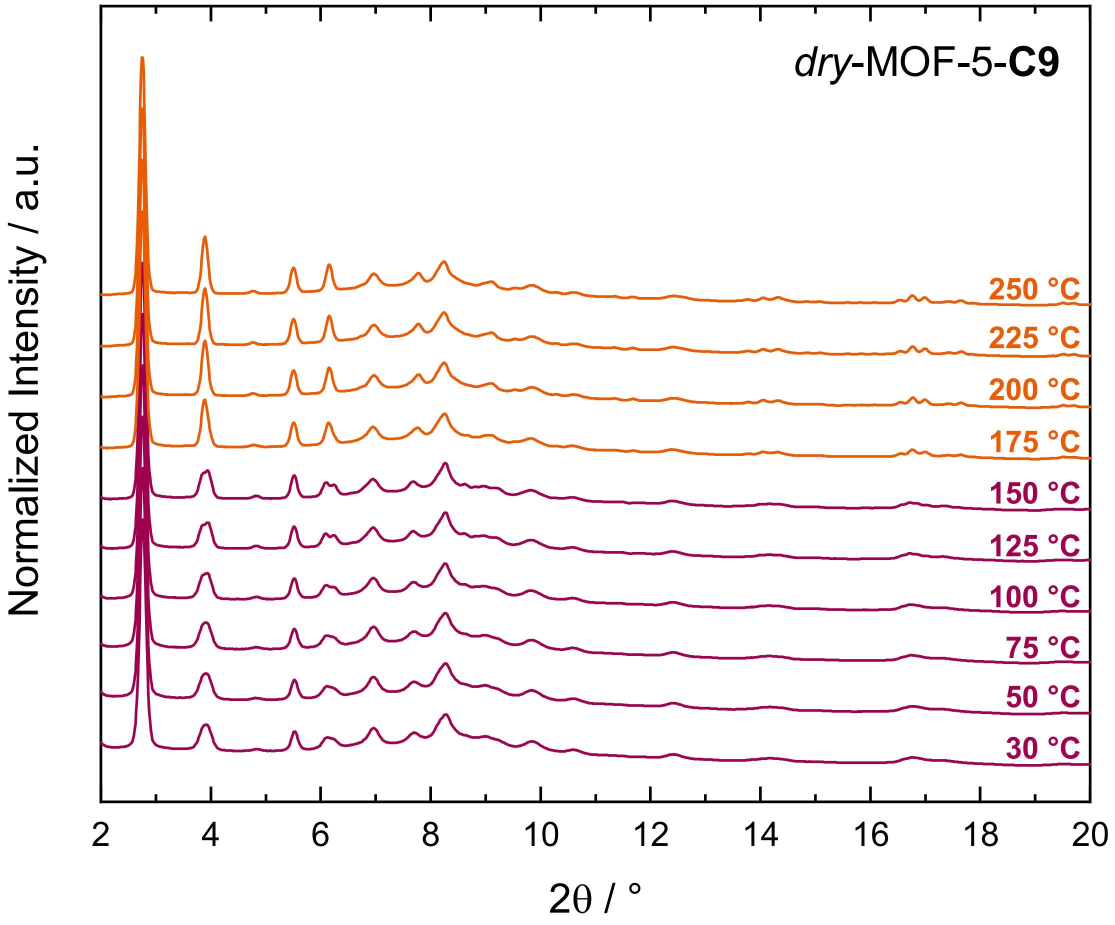

Supplementary Figure 12.8: Variable temperature PXRD pattern of dry-MOF-5-C9 recorded in the range from 30 to 250 °C.

Supplementary References

1. Henke, S., Schneemann, A., Wütscher, A. & Fischer, R. A. Directing the breathing behavior of pillared-layered metal-organic frameworks via a systematic library of functionalized linkers bearing flexible substituents. *J. Am. Chem. Soc.* **134**, 9464–9474 (2012).

2. Grunder, S. *et al.* Molecular gauge blocks for building on the nanoscale. *Chem. Eur. J.* **18**, 15632–15649 (2012).

3. Fletcher, Steven; Gunning, P. T. Mild, efficient and rapid O-debenzylation of ortho-substituted phenols with trifluoroacetic acid. *Tetrahedron Lett.* **49**, 4817–4819 (2008).

4. Zhu, N. X. *et al.* Self-Generation of Surface Roughness by Low-Surface-Energy Alkyl Chains for Highly Stable Superhydrophobic/Superoleophilic MOFs with Multiple Functionalities. *Angew. Chem. Int. Ed.* **58**, 17033–17040 (2019).

5. Henke, S., Schmid, R., Grunwaldt, J. D. & Fischer, R. A. Flexibility and sorption selectivity in rigid metal-organic frameworks: The impact of ether-functionalised linkers. *Chem. Eur. J.* **16**, 14296–14306 (2010).

6. Eddaoudi, M. *et al.* Systematic design of pore size and functionality in isoreticular MOFs and their application in methane storage. *Science* **295**, 469–472 (2002).

7. Ma, L., Jin, A., Xie, Z. & Lin, W. Freeze drying significantly increases permanent porosity and hydrogen uptake in 4,4-connected metal-organic frameworks. *Angew. Chem. Int. Ed.* **48**, 9905–9908 (2009).

8. Zhang, Y. B. *et al.* Introduction of functionality, selection of topology, and enhancement of gas adsorption in multivariate metal-organic framework-177. *J. Am. Chem. Soc.* **137**, 2641–2650 (2015).

9. Kaye, S. S., Dailly, A., Yaghi, O. M. & Long, J. R. Impact of preparation and handling on the hydrogen storage properties of Zn4O(1,4-benzenedicarboxylate)3 (MOF-5). *J. Am. Chem. Soc.* **129**, 14176–14177 (2007).

10. Coelho, A. A. TOPAS and TOPAS-Academic: An optimization program integrating computer algebra and crystallographic objects written in C++: An. *J. Appl. Cryst.* **51**, 210–218 (2018).

11. Thompson, P., Cox, D. E. & Hastings, J. B. Rietveld Refinement of Debye-Scherrer Synchrotron X-ray Data from Al203. *J. Appl. Cryst.* **20**, 79–83 (1987).

12. Dortmund Data Bank. Dortmund Data Bank (DDB). *Surface tension of hexane* (2020).

13. Debgupta, J., Kakade, B. A. & Pillai, V. K. Competitive wetting of acetonitrile and dichloromethane in comparison to that of water on functionalized carbon nanotube surfaces. *Phys. Chem. Chem. Phys.* **13**, 14668–14674 (2011).

14. Chae, H. K. *et al.* A route to high surface area, porosity and inclusion of large molecules in crystals. *Nature* **427**, 523–527 (2004).

15. Amirjalayer, S. & Schmid, R. Conformational isomerism in the isoreticular metal organic framework family: A force field investigation. *J. Phys. Chem. C* **112**, 14980–14987 (2008).

16. Spek, A. L. Single-crystal structure validation with the program PLATON. *J. Appl.*

Cryst. **36**, 7–13 (2003).

17. Li, H., Eddaoudi, M., O'Keeffe, M. & Yaghi, O. M. Design and synthesis of an exceptionally stable and highly porous metal-organic framework. *Nature* **402**, 276–279 (1999).

18. Yang, W. *et al.* Selective CO 2 uptake and inverse CO 2/C 2H 2 selectivity in a dynamic bifunctional metal-organic framework. *Chem. Sci.* **3**, 2993–2999 (2012).

19. Brunauer, S., Emmett, P. H. & Teller, E. Adsorption of Gases in Multimolecular Layers. *J. Am. Chem. Soc.* **60**, 309–319 (1938).

20. Rogge, S. M. J., Waroquier, M. & Van Speybroeck, V. Reliably Modeling the Mechanical Stability of Rigid and Flexible Metal-Organic Frameworks. *Acc. Chem. Res.* **51**, 138–148 (2018).

21. Rogge, S. M. J., Waroquier, M. & Van Speybroeck, V. Unraveling the thermodynamic criteria for size-dependent spontaneous phase separation in soft porous crystals. *Nat. Commun.* **10**, 4842 (2019).

22. Evans, J. D., Bocquet, L. & Coudert, F. X. Origins of Negative Gas Adsorption. *Chem* **1**, 873–886 (2016).

23. Keupp, J. & Schmid, R. Molecular Dynamics Simulations of the "Breathing" Phase Transformation of MOF Nanocrystallites. *Adv. Theory Simul.* **2**, 1900117 (2019).

24. Vervoorts, P. *et al.* Configurational Entropy Driven High-Pressure Behaviour of a Flexible Metal–Organic Framework (MOF). *Angew. Chem. Int. Ed.* **60**, 787–793 (2021).

25. Lock, N., Christensen, M., Kepert, C. J. & Iversen, B. B. Effect of gas pressure on negative thermal expansion in MOF-5. *Chem. Commun.* **49**, 789–791 (2013).

26. Lock, N. *et al.* Elucidating negative thermal expansion in MOF-5. *J. Phys. Chem. C* **114**, 16181–16186 (2010).

27. Rogge, S. M. J. *et al.* A Comparison of Barostats for the Mechanical Characterization of Metal-Organic Frameworks. *J. Chem. Theory Comput.* **11**, 5583–5597 (2015).
